# Fluorescent small molecule donors

**DOI:** 10.1039/d3cs00124e

**Published:** 2024-05-14

**Authors:** Guang Chen, Jing Yu, Luling Wu, Xinrui Ji, Jie Xu, Chao Wang, Siyue Ma, Qing Miao, Linlin Wang, Chen Wang, Simon E. Lewis, Yanfeng Yue, Zhe Sun, Yuxia Liu, Bo Tang, Tony D. James

**Affiliations:** a The Youth Innovation Team of Shaanxi Universities, Shaanxi Key Laboratory of Chemical Additives for Industry, College of Chemistry and Chemical Engineering, Shaanxi University of Science & Technology Xi’an 710021 China chenandguang@163.com liuyuxia2008@163.com; b Department of Chemistry, University of Bath Bath BA2 7AY UK t.d.james@bath.ac.uk; c College of Chemistry, Chemical Engineering and Materials Science, Shandong Normal University Jinan 250014 Shandong China tangb@sdnu.edu.cn; d Institute of Molecular Plus, Tianjin Key Laboratory of Molecular Optoelectronic Sciences, Tianjin University 92 Weijin Road Tianjin 300072 China zhesun@tju.edu.cn; e Department of Chemical Engineering and Waterloo Institute for Nanotechnology, University of Waterloo 200 University Avenue West Waterloo Ontario N2L 3G1 Canada; f Department of Chemistry, Delaware State University Dover DE 19901 USA yyue@desu.edu; g School of Chemistry and Chemical Engineering, Henan Normal University Xinxiang 453007 China

## Abstract

Small molecule donors (SMDs) play subtle roles in the signaling mechanism and disease treatments. While many excellent SMDs have been developed, dosage control, targeted delivery, spatiotemporal feedback, as well as the efficiency evaluation of small molecules are still key challenges. Accordingly, fluorescent small molecule donors (FSMDs) have emerged to meet these challenges. FSMDs enable controllable release and non-invasive real-time monitoring, providing significant advantages for drug development and clinical diagnosis. Integration of FSMDs with chemotherapeutic, photodynamic or photothermal properties can take full advantage of each mode to enhance therapeutic efficacy. Given the remarkable properties and the thriving development of FSMDs, we believe a review is needed to summarize the design, triggering strategies and tracking mechanisms of FSMDs. With this review, we compiled FSMDs for most small molecules (nitric oxide, carbon monoxide, hydrogen sulfide, sulfur dioxide, reactive oxygen species and formaldehyde), and discuss recent progress concerning their molecular design, structural classification, mechanisms of generation, triggered release, structure–activity relationships, and the fluorescence response mechanism. Firstly, from the large number of fluorescent small molecular donors available, we have organized the common structures for producing different types of small molecules, providing a general strategy for the development of FSMDs. Secondly, we have classified FSMDs in terms of the respective donor types and fluorophore structures. Thirdly, we discuss the mechanisms and factors associated with the controlled release of small molecules and the regulation of the fluorescence responses, from which universal guidelines for optical properties and structure rearrangement were established, mainly involving light-controlled, enzyme-activated, reactive oxygen species-triggered, biothiol-triggered, single-electron reduction, click chemistry, and other triggering mechanisms. Fourthly, representative applications of FSMDs for trackable release, and evaluation monitoring, as well as for visible *in vivo* treatment are outlined, to illustrate the potential of FSMDs in drug screening and precision medicine. Finally, we discuss the opportunities and remaining challenges for the development of FSMDs for practical and clinical applications, which we anticipate will stimulate the attention of researchers in the diverse fields of chemistry, pharmacology, chemical biology and clinical chemistry. With this review, we hope to impart new understanding thereby enabling the rapid development of the next generation of FSMDs.

## Abstract 2

Recent progress in the molecular design, structural classification, mechanisms of generation, triggered release, structure–activity relationships, and fluorescence response mechanism of fluorescent small molecule donors is discussed.

## Graphical abstract

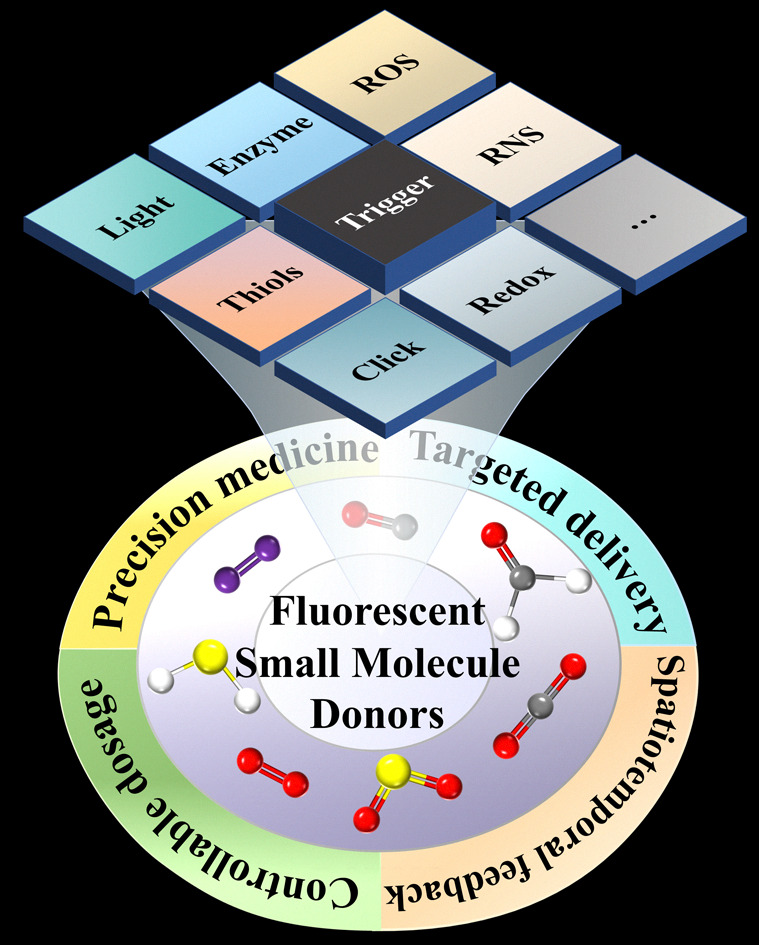

## 1. Introduction

At present, nitric oxide (NO), carbon monoxide (CO), hydrogen sulfide (H_2_S), sulfur dioxide (SO_2_), reactive oxygen species (ROS), hydrogen selenide (H_2_Se), carbon dioxide (CO_2_), and formaldehyde (FA) are considered to be important gaseous signaling molecules in regulating a variety of physiological and pathological processes.^1–10^ For example, NO is an effective inhibitor of platelet activation and aggregation, which can block thrombosis;^11–13^ CO can effectively induce vasodilation in an environment of oxidative stress;^14–16^ administration of H_2_S to the cardiovascular system directly activates the ATP-sensitive potassium channel, thereby promoting a decrease in arterial blood pressure;^17–20^ the synergistic effect of SO_2_ with doxorubicin (DOX) effectively alleviates multidrug resistance in cancer chemotherapy.^21,22^ Clearly, SMDs represent a promising approach to eradicate serious diseases. However, for the development of such donors, a controllable dosage, targeted delivery, spatiotemporal feedback, and precise medical treatment are still key challenges. Fortunately, FSMDs can realize not only the triggered release but also the real-time monitoring of active small molecules in organisms, thereby providing remarkable advantages to meet those challenges. For example, the NO-releasing platform with ciprofloxacin loading can eradicate *Pseudomonas aeruginosa* biofilms *in vitro* and accelerate wound healing *in vivo*.^23^ Metal-free CO donors based on structurally adjustable flavonols exhibit significant anti-inflammatory efficiency.^24^ While ROS-responsive, self-immolative, and fluorescent H_2_S donors can serve as theranostic agents for myocardial infarction and other ischemic diseases.^25^

To design FSMDs, the following basic characteristics need to be considered: (i) specific trigger: the donor can be activated by the specific environment to exclusively release the small molecule. (ii) Efficient release rate: during the rearrangement, intermediates are generated that release the small molecules. Therefore, a deep understanding of the reaction mechanisms is required. (iii) Penetration and targeting: donors need to penetrate biological barriers, and actively target regions of interest, such as the tumor microenvironment or subcellular organelles. (iv) Distinguishable fluorescence switches: during the release of small molecules, a continuous visible fluorescence response is required to enable real-time monitoring and evaluation. (v) Good biocompatibility, negligible cytotoxicity and definite by-products. Preferably, FSMDs with good biological compatibility will accelerate practical applicability. As such FSMDs contain the advantages of both SMDs and the fluorescent probes and can serve as a dynamic monitoring and supplier of active small molecules for drug screening and precision medicine.

This review covers the latest developments toward FSMDs that meet the following requirements. Based on available FSMDs, we collated the common structures for generating different types of small molecules, thereby, providing some generalizations for the design of FSMDs. The central structures include the *N*-nitroso-type of NO donors, CO donors based on 3-hydroxyflavone derivatives, thiocarbamate-based H_2_S donors with self-immolation ability, SO_2_ donors with 2,4-dinitrobenzene sulfonic acid as the core, and H_2_Se donors generated by modifying GYY4137, therefore providing a general template for the design of FSMDs. The interlacing mechanisms and factors controlling the release of small molecules and the regulation of fluorescence responses are discussed, from which universal strategies are summarized, mainly including light-controlled, enzyme-activated, reactive oxygen species-triggered, biothiol-triggered, single-electron reduction, and click chemistry. We outline the feedback regulation mechanisms between small molecule release and fluorescence response. When small molecules are released, the charge transfer mechanism in the donor will lead to significant fluorescence changes (fluorescence quenching, fluorescence recovery, ratiometric or emission shift). In most cases, a fluorescence response is also a prerequisite for the release of small molecules (for example, aza-BODIPY integrates light triggering and fluorescence response). Therefore, there is a positive feedback regulation mechanism between the release of the small molecule and fluorescence response. In addition, the factors affecting the release of small molecules from FSMDs are explored: the non-planar torsional conformation of the substituent with respect to the aromatic ring, the degree of electronic delocalization between the release unit and the fluorophore framework, *etc.*, which all influence the ability of FSMDs to release small molecules. Finally, we concentrate on the design strategies toward FSMD prodrugs and their applications for cancer treatment. Therefore, providing feasible solutions for the development of FSMDs with dose controllable, targeted release, spatiotemporal feedback, and precision medical properties, as well as pro-drug design strategies for the development of clinical drugs based on the controlled release of small molecules. Table 1 lists the simplified basic structures of FSMDs discussed in this review (the groups released are highlighted in red).

**Table 1:** Platforms for fluorescent small molecules donors

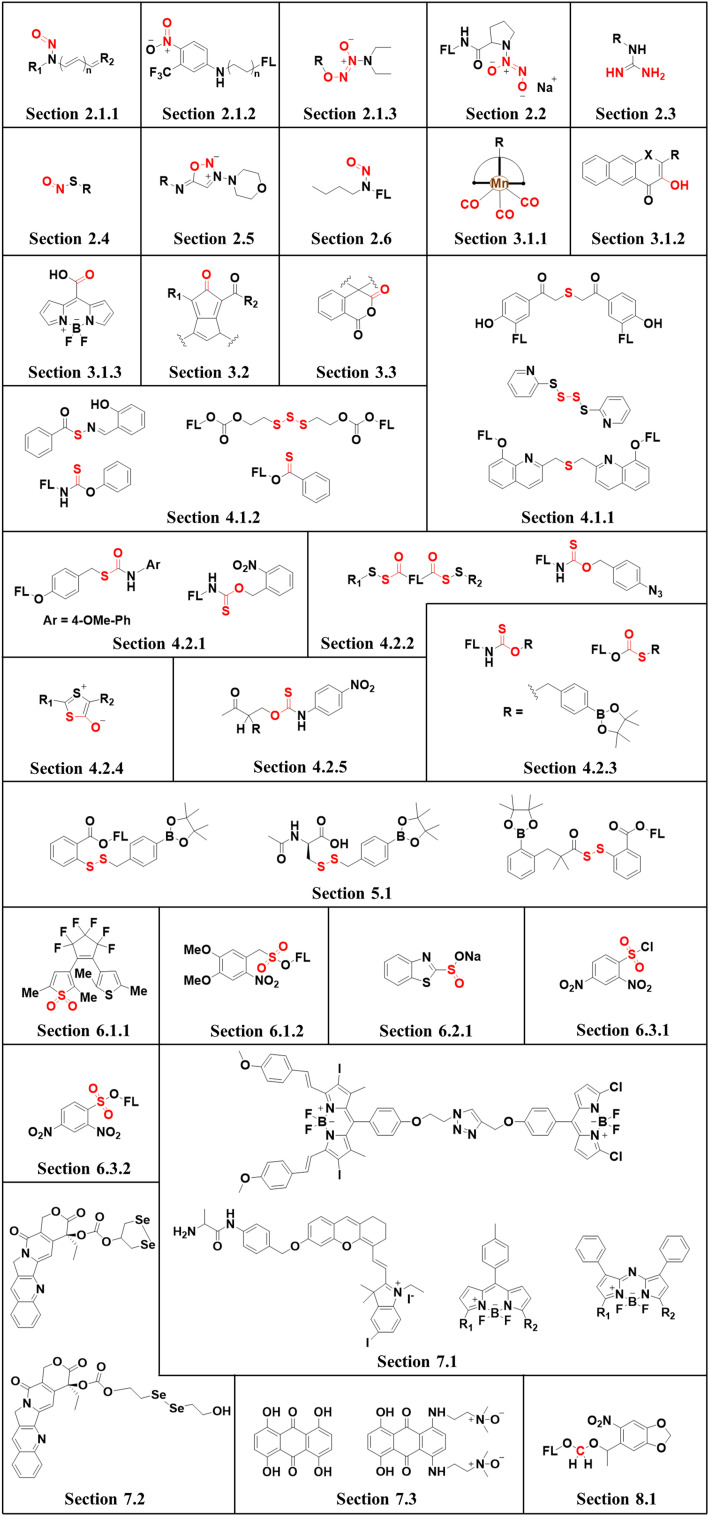

## 2. Nitric oxide

NO has long been recognized as a detrimental atmospheric pollutant originating from industrial processes and gasoline combustion.^26^ However, in 1998, research conducted by Robert F. Furchgott, Louis J. Ignarro, and Ferid Murad unveiled NO as an endogenous endothelial-derived vasodilator, earning them the Nobel Prize in Physiology or Medicine.^27^ Since then, intensive investigations into the biological and medical aspects of NO were initiated. NO exhibits various biological functions, involved in virtually all human physiological and pathological processes.^28^ It mediates vasodilation,^29^ confers cardioprotection,^12^ facilitates wound healing,^30^ treats osteoporosis,^31^ and regulates renal metabolism^2^ and neurogenesis.^32^ In addition, the concentration of NO closely correlates with cancer progression, low concentrations of NO promote cell proliferation while high concentrations of NO induce cell oxidative stress.^33^ As such, due to the diverse biological functions of NO, there has been significant interest in NO donors.

### 2.1. Photo controlled NO release

#### 2.1.1. *N*-Nitroso compounds

**Fig. 1:**
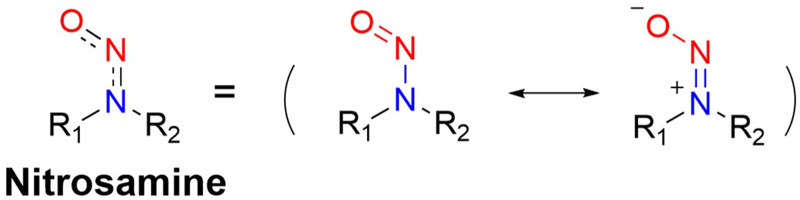
Resonance structure of *N*-nitrosamines.

**Fig. 2:**
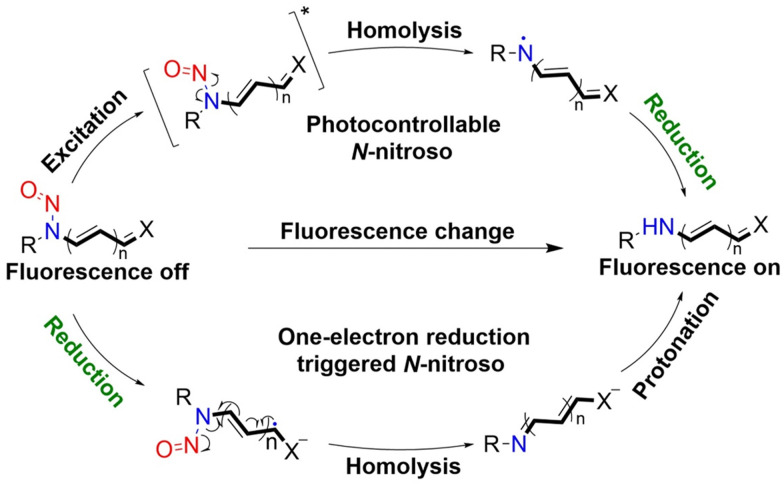
Light-controlled/one-electron reduction triggers the NO-release mechanism of *N*-nitroso push–pull dyes and their fluorescence changes (R = fluorophore X = alkyl, aryl, *etc.*).

It is well known that *N*-nitroso compounds can release NO and amino radicals under light irradiation *via* the radical decomposition reaction.^34,35^ Moreover, due to the conjugation effect between the lone pair electrons on the amino–N and the double bond of nitroso, there is a partial double bond between N–N, resulting in the H on the α-C being easily oxidized (Fig. 1). While if the α-C is attached to an electron-withdrawing group, it will cause the density of the electron cloud on α-C and N–NO to decrease, which enhances the stability of the free radicals. *N*-Nitroso can release NO *via* two mechanisms: under light irradiation, homolysis occurs to release a NO and amino radicals, which is subsequently reduced *via* hydrogen abstraction to yield the corresponding push–pull type dye for photo-calibration, *i.e.* the photo-triggered homolysis (Fig. 2, upper). Furthermore, when the chromophoric scaffold in this NO donor is a strong electron-withdrawing group, it can replace the photolysis pathway of *N*-nitroso, that is, NO release is by a reduction-triggered protonation cleavage pathway (R = chromophoric scaffold = a strong electron-withdrawing group)^36^ (Fig. 2, lower). As a result, the visible monitoring of NO release by spectral or microscopic methods can be achieved. In addition, tumor growth rate and treatment can be visually monitored.

**Fig. 3:**
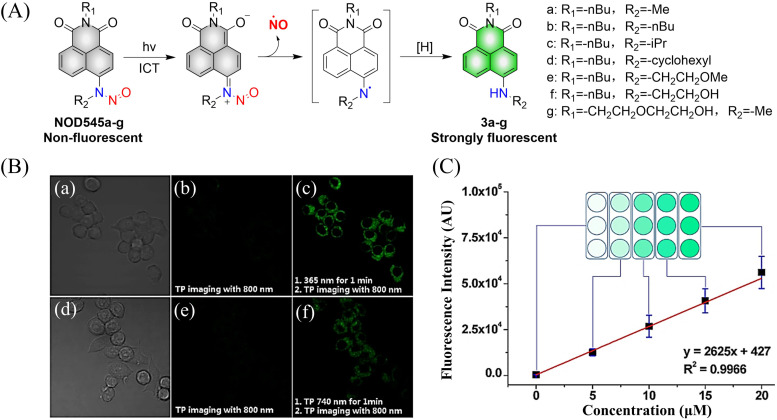
(A) The structure of NOD545a–g, the mechanism of NO release and the fluorescence changes. (B) RAW 264.7 cells incubated with NOD545f for two-photon fluorescence imaging. (C) HeLa cells incubated with different concentrations of 3f to investigate the relationship between fluorescence intensity and cell culture concentration. Parts (B) and (C) are reproduced from ref. 37 with the permission of the American Chemical Society, copyright 2016.

Considering the importance of *N*-nitroso compounds, researchers have used various fluorophores to develop FSMDs for the monitoring of NO release. The Yang group developed a series of NO donors (NOD545a–g), which was a donor that exhibited a large fluorescence response with the light triggered release of NO (Fig. 3(A)).^37^ Naphthalimide was selected as the fluorophore since its fluorescence quantum yield is high and its structure is easily modified. In addition, under 365 nm ultraviolet or 740 nm two-photon irradiation, NOD545a–g undergoes ICT, and the *N*-nitroso part transforms into an electron-rich N–H bond, thus releasing NO. At the same time, anilinyl radical by-products are produced, which are easily reduced *in situ*, generating 4-amino naphthalimide fluorophores (3a–g). As a result, the strong electron donor group at the 4-position contributes to strong fluorescence enhancement (up to 800-fold) and enables the real-time monitoring of NO release by spectral or microscopic methods (Fig. 3(B) and (C)).

**Fig. 4:**
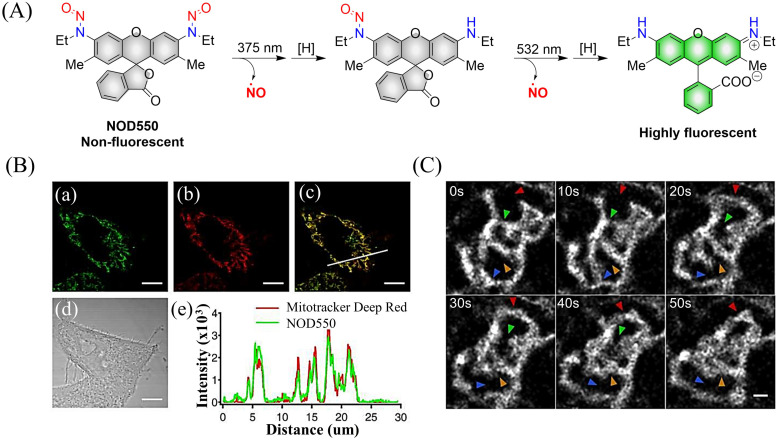
(A) The structure of NOD550, the mechanism of NO release and its fluorescence changes. (B) NOD550 and Mitotracker Deep Red were used for two-color localization imaging. (C) Super resolution monitoring of mitochondrial dynamics. Parts (B) and (C) are reproduced from ref. 39 with the permission of the American Chemical Society, copyright 2018.

Although the previously reported NOD545a–g donors enable monitoring of NO release, the naphthalimide fluorophores cannot realize targeted release. In order to break through this limitation, the Yang group developed the NO donors (NOD550) targeting the mitochondria using *N*-nitroso caged rhodamine fluorophores (single molecule localization super-resolution imaging^38^).^39^ The donor enabled the visual monitoring of mitochondrial morphology and dynamics while releasing NO. For example, under 375 nm and 532 nm light irradiation, NOD550 releases two NO molecules and one rhodamine fluorophore (Fig. 4(A)–(C)).

**Fig. 5:**
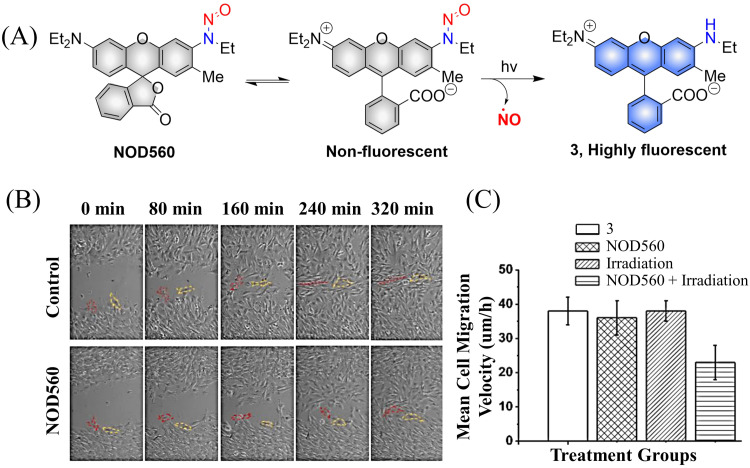
(A) The structure of NOD560, the mechanism of NO release and its fluorescence changes. (B) Migration of MSCs in wound models. (C) Average migration speed of MSCs under different conditions. Reproduced from ref. 40 with the permission of Elsevier Inc., copyright 2018.

It was determined that NOD560 can be effectively activated by light over a wavelength range from 375–561 nm. The releases of NO simultaneously generates the rhodamine fluorophore. The resultant bright fluorescence is turned on and enables the monitoring of the NO release (Fig. 5(A)).^40^ In addition, NOD560 exhibits excellent characteristics, it can inhibit migration of mouse mesenchymal stem cells (MSCs) (Fig. 5(B) and (C)). The main disadvantage is that the photolysis rate is about 2300 s, and the dynamics are relatively slow.

**Fig. 6:**
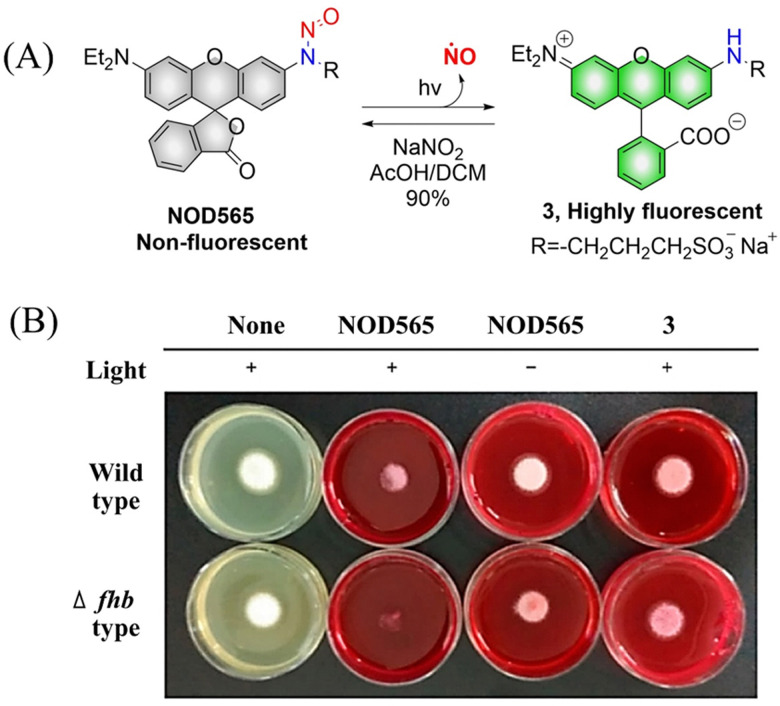
(A) The structure of NOD565, the mechanism of NO release and its fluorescence changes. (B) NOD565 inhibits fungal growth. Reproduced from ref. 41 with the permission of the American Chemical Society, copyright 2018.

NOD565 is a water-soluble, green light triggered and light calibrated NO donor, that can effectively release NO and rhodamine fluorophores (3) under 532 nm green light irradiation (Fig. 6(A)).^41^ Compared with the UV triggered NO donor previously discussed, NOD565 can be triggered to release NO by a longer-wavelength of 532 nm. In addition, *in vitro* studies have shown that NOD565 exhibits great potential for inhibiting platelet aggregation and fungal growth (Fig. 6(B)).

**Fig. 7:**
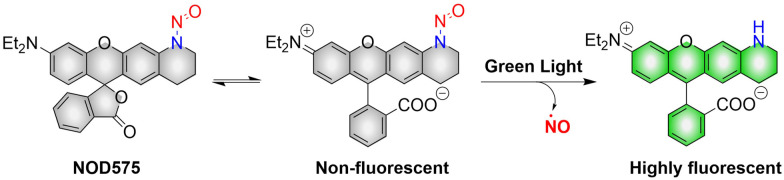
The structure of NOD575, the mechanism of NO release and its fluorescence changes.

NOD575 due to its special structure (the extended six membered ring structure makes the *N*-nitroso and the fluorophore framework coplanar, promoting the electron delocalization of the N atom to the fluorophore framework), NOD575 can rapidly release NO under 532 nm light irradiation (Fig. 7).^42^ Compared with NOD560, the kinetics of NO release from NOD575 were enhanced about 20-fold.

**Fig. 8:**
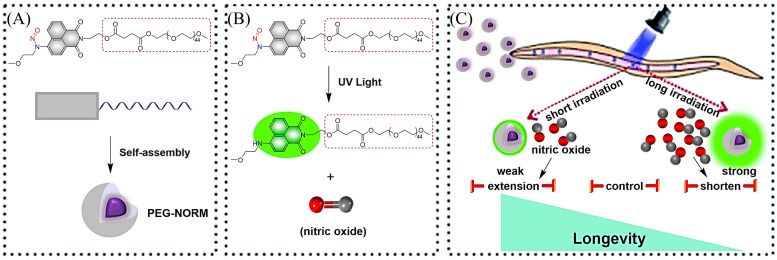
(A) Formation of PEG-NORM nanoparticles. (B) Proposed photodissociation mechanism of PEG-NORM. (C) Effect of PEG-NORM on longevity of *C. elegans*. Reproduced from ref. 47 with the permission of the Royal Society of Chemistry, copyright 2020.

It is well known that low concentrations of NO promote cell proliferation while high concentrations of NO induce cell oxidative stress.^43–46^ Therefore, it is essential to develop NO donors that can control the concentration and dose. The Zhang group introduced a polyethylene glycol (PEG) group and prepared a new type of NO donor nanoparticle (PEG-NORM) through self-assembly (Fig. 8(A)).^47^ PEG-NORM homolyzes upon photolysis to yield NO generating a fluorescence response, where the released NO concentration is dependent on the light intensity and duration. As such, this system can regulate biological processes more efficiently. For example, increasing the light intensity and time, the higher concentrations of NO can cause apoptosis of *C. elegans* germ cells and reduce the survival duration (Fig. 8(B) and (C)). Thus, the frontiers of NO biology have shifted from the elucidation of mechanisms to biomanipulation, laying the foundation for significant biological and medicinal progress for NO related systems.

**Fig. 9:**
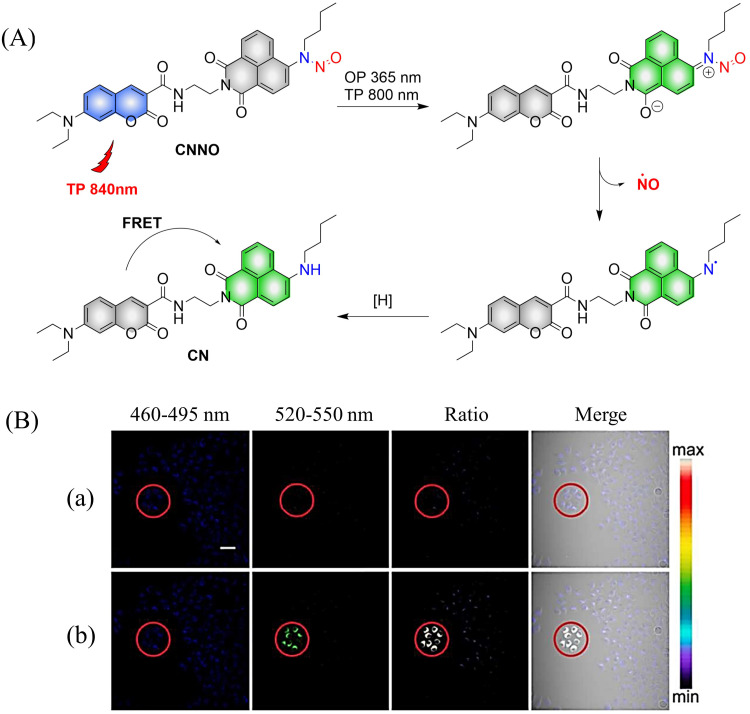
(A) The structure of CNNO, the mechanism of NO release and its fluorescence changes. (B) Two-photon fluorescence imaging of NO release by CNNO in HeLa cells. Cells were treated with 10 μM CNNO. (a) The dish was imaged without light irradiation. (b) The dish was irradiated inside the selected circle using a two-photon laser at 800 nm (20 mW) for 2 min before being imaged. Reproduced from ref. 48 with the permission of the Royal Society of Chemistry, copyright 2017.

Two-photon imaging has many advantages, such as high resolution, enhanced imaging depth, and low background signals. Therefore, it is very important for the research of diseases in living animals. The Tang group have developed a two-photon NO donor (CNNO) (Fig. 9(A)).^48^ Under 365 nm or 800 nm two-photon irradiation, CNNO releases NO and FRET is activated due to a red-shift in the absorption spectrum of the naphthalimide receptor, which then overlaps with the emission spectrum of the coumarin donor. This system can be used to monitor NO release using two-photon ratiometric fluorescence, and the fluorescence response is not influenced by complex biological environments (Fig. 9(B)). With these advantages, the authors found that NO released by CNNO in the aorta of mice can control vasodilation.

**Fig. 10:**
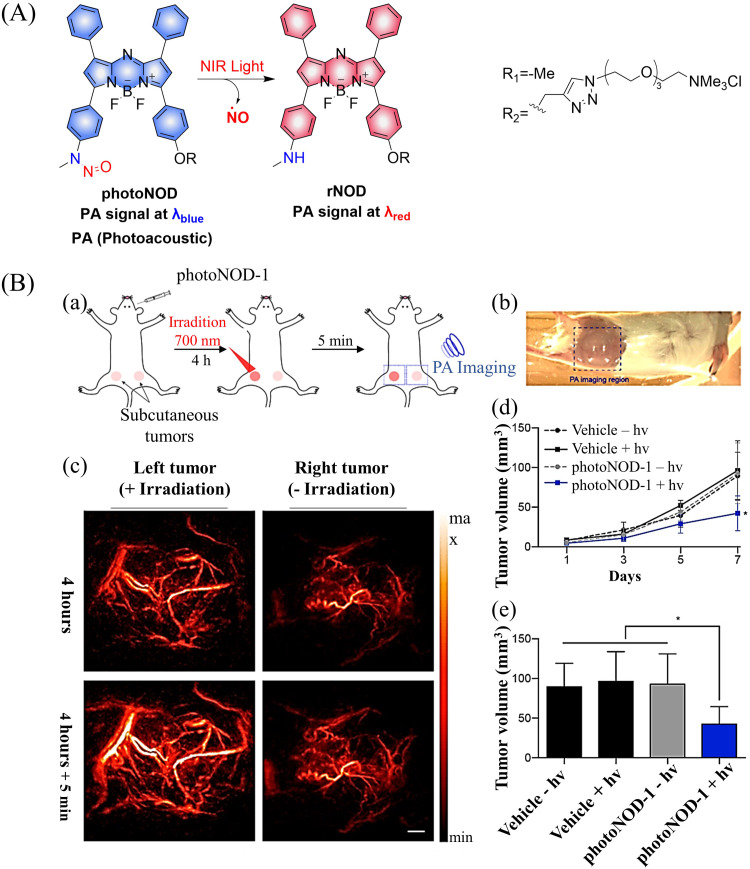
(A) The structure of photoNODs, the mechanism of NO release and its PA (photoacoustic) changes. (B) (a) Schematic illustration of photoNOD-1 administration, NO treatment, and PA monitoring. (b) Photograph of mouse imaged by a PA tomographer. Dashed line indicates region of PA imaging. (c) PA images (*λ*_PAred_) acquired before/after a 5 min period with/without irradiation (*λ*_PAblue_) 4 h following systemic administration of photoNOD-1 (1.2 mg kg^−1^, 150 μL, 20% DMSO in sterile saline). (d) and (e) Measured tumor volume under different treatment conditions. Reproduced from ref. 55 with the permission of the American Chemical Society, copyright 2018.

Photoacoustic (PA) tomography enables the imaging of biological tissues at centimeter depths using an NIR trigger and ultrasonic detection, providing accurate guidance for the treatment of cancer.^49–54^ When combined with small molecule donors, deep tissue gas therapy can be readily monitored. The Chan group reported a NO donor based on PA (photoNODs).^55^ Upon single-photon NIR irradiation, photoNOD-1 and photoNOD-2 release NO as well as rNOD-1 or rNOD-2, PA-active products that enable ratiometric monitoring of NO release. Significantly different PA signals enable the visual monitoring of NO release (Fig. 10(A)). In addition, photoNOD-1 can selectively influence tumor growth rates, providing a new concept for cancer treatment (Fig. 10(B)). Significantly, this design strategy could be extended to other analytes, thereby expanding the scope of synergistic NIR photorelease and PA imaging.

**Fig. 11:**
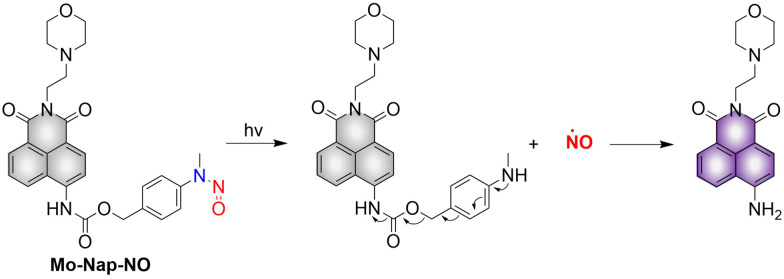
The structure of Mo-Nap-NO, the mechanism of NO release and its fluorescence changes.

Lysosomes are important organelles, which are not only the degradation centers of cells through autophagy, but also mediate endocytosis and the transfer of extracellular substances.^56,57^ The Gou research group have developed a NO donor that targets lysosomes for anticancer therapy (Mo-Nap-NO).^58^ Mo-Nap-NO uses the *N*-nitroso as the NO donor, 4-amino-1,8-naphthalimide as the fluorescent moiety, and morpholine as the lysosomal targeting unit. Under 460 nm light irradiation, Mo-Nap-NO undergoes intramolecular electron transfer, releasing NO and the fluorescence is turned on (Fig. 11). In addition, the localized release of NO can rupture the lysosome, resulting in the release of cathepsin D into the cytoplasm, thus activating Caspase-3 mediated apoptosis and inhibiting the development of cancer cells.

**Fig. 12:**
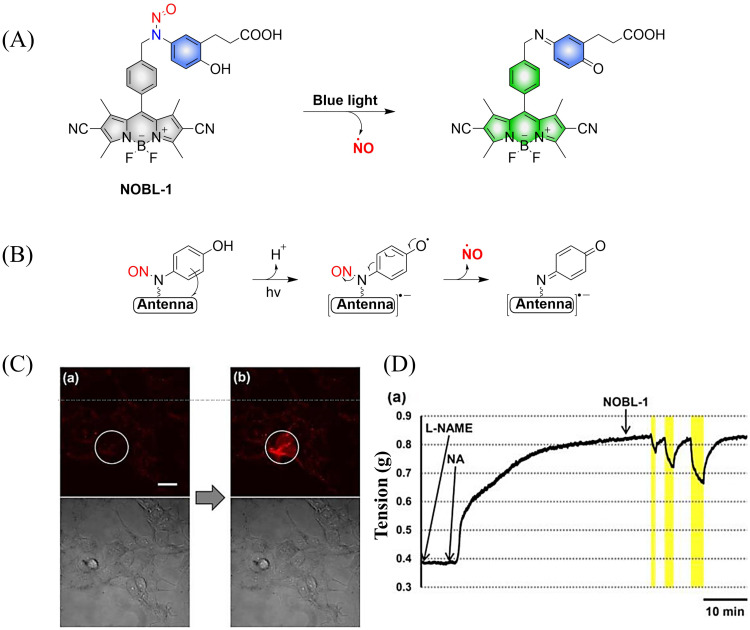
(A) and (B) The structure of NOBL-1, the mechanism of NO release and its fluorescence changes. (C) DAR-4M AM (a fluorogenic NO probe) monitored fluorescence imaging of NO released by NOBL-1 in HEK293 cells. (D) Blue-light activated NOBL-1 to release NO induced vasodilation changes in rats. Parts (C) and (D) are reproduced from ref. 59 with the permission of the American Chemical Society, copyright 2014.

To avoid the toxicity of ultraviolet light, Nakagawa and colleagues have developed a light controllable (*λ*_max_ ≈ 500 nm) NO donor (NOBL-1), which is composed of an *N*-nitroso part as NO release unit and BODIPY as fluorescent dye (antenna). Moreover, the structure of NOBL-1 also contains carboxyl groups that can increase the hydrophilicity, making NOBL-1 suitable for biological applications (Fig. 12(A)).^59^ NOBL-1 can release NO by photoinduced electron transfer (PeT). When BODIPY is excited, single electron transfer from the *N*-nitrosoaminophenol to the BODIPY occurs, thus forming a stable quinone with NO release (Fig. 12(B)). Based on the visual-monitoring of NO release, the authors confirmed that vasodilation of the rat aorta could be controlled by light-induced NO release from NOBL-1 (Fig. 12(C) and (D)).

**Fig. 13:**
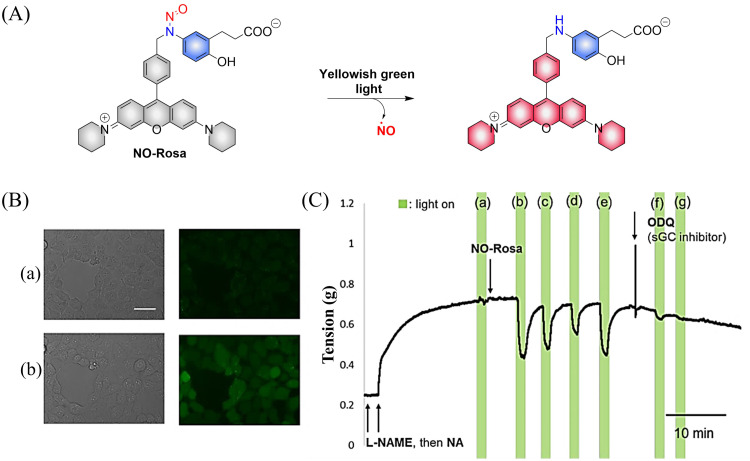
(A) The structure of NO-Rosa, the mechanism of NO release and its fluorescence changes. (B) DAR-4M AM (a fluorogenic NO probe) monitored fluorescence imaging of NO released by NO-Rosa in HEK293 cells. (C) NO-Rosa activated by yellowish-green-light to release NO and induce vasodilation changes in rats. Parts (B) and (C) are reproduced from ref. 60 with the permission of the Royal Society of Chemistry, copyright 2017.

Subsequently, the research group designed and synthesized a new yellowish-green-light controllable (*λ*_max_ ≈ 550 nm) NO donor (NO-Rosa) (Fig. 13(A)).^60^ Compared with the previous system this system uses longer wavelength excitation (yellowish-green-light), which displays good tissue penetration and less cytotoxicity. The structure of NO-Rosa is very similar to that of NOBL-1, except for the difference in the fluorophore part (BODIPY for NOBL-1 and rosamine for NO-Rosa), which leads to the absorption of yellowish-green-light. NO-Rosa also exhibited the advantages of time-controlled and visual-monitoring of rat aorta vasodilatation (Fig. 13(B) and (C)).

**Fig. 14:**
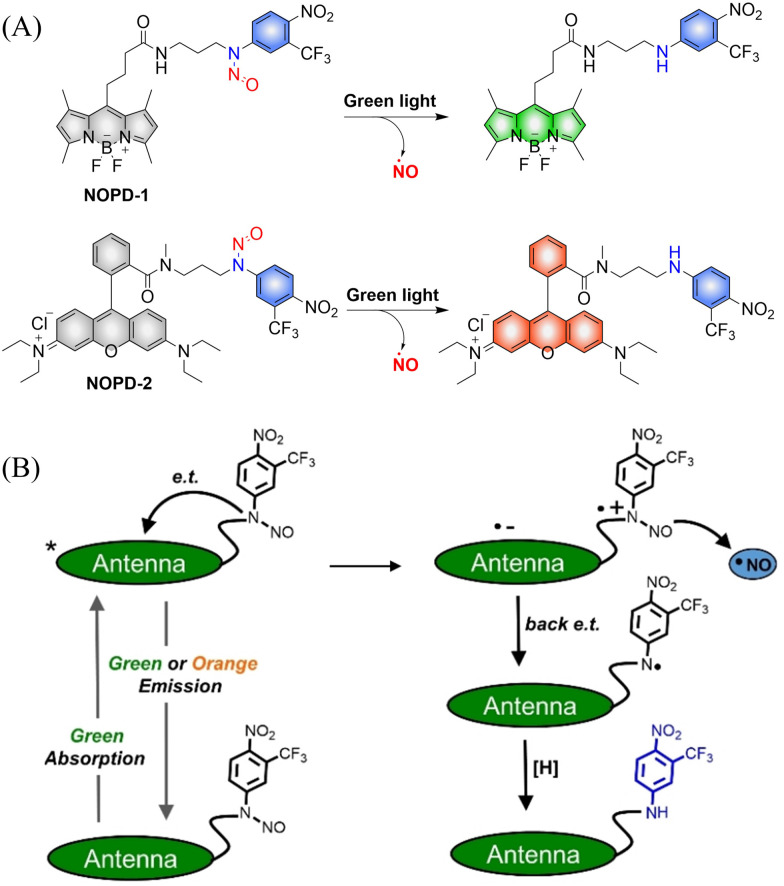
(A) The structure of NOPDs, and fluorescence changes. (B) Proposed photodissociation mechanism of NOPD-1/2. Reproduced from ref. 61 with the permission of Wiley-VCH Verlag GmbH & Co. KGaA, Weinheim, copyright 2019.

The Sortino research group reported two *N*-nitroso NO photo donors (NOPDs) based on nitrobenzene compounds in 2019.^61^ The authors used BODIPY and Rhodamine as the fluorophores, and both of them can release NO under irradiation with biocompatible green light. The systems exhibit green and orange-red fluorescence respectively, which is convenient for the localization and tracking of NO release (Fig. 14(A) and (B)).

**Fig. 15:**
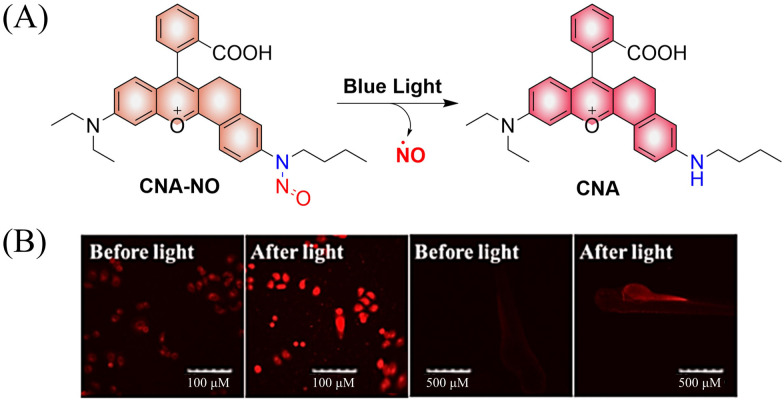
(A) The structure of CNA-NO, the mechanism of NO release and its fluorescence changes. (B) Comparison of fluorescence imaging of CNA-NO in living cells and zebrafish before and after illumination. Reproduced from ref. 62 with the permission of Elsevier B.V., copyright 2021.

To improve the fluorescence signal and quantum yield, Qian and colleagues, developed a water-soluble light-induced NO donor (CNA-NO) (Fig. 15(A)).^62^ In their design strategy, the introduction of cyclohexyl into CNA-NO can restrict the rotation of the benzene, decreasing the non-radiative energy loss and thereby enhancing the fluorescence signal and quantum yield of this donor. Under light irradiation of 465 nm, the fluorescence of CNA-NO increases (78-fold) and releases NO (Fig. 15(B)). The probe exhibits good biocompatibility which ensures that the donor can be used in living organisms.

**Fig. 16:**
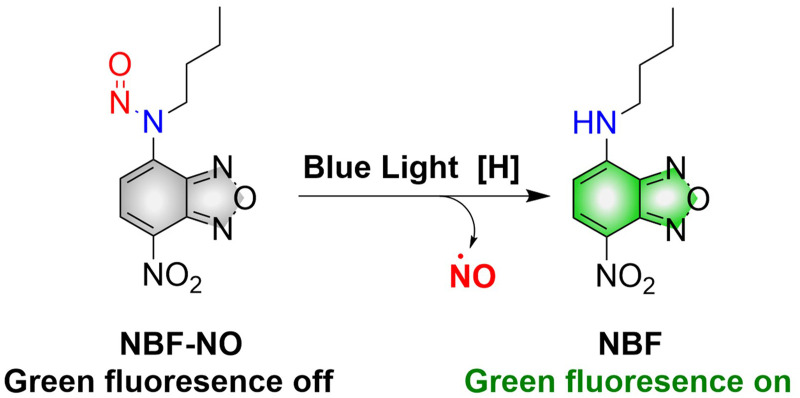
The structure of NBF-NO, the mechanism of NO release and fluorescence changes.

Although a large number of excellent donors have been developed, their synthesis is often complicated. Therefore, it is necessary to develop functional donors that are easy to prepare. Sortino, *et al.* reported a green fluorescent NO donor (NBF-NO).^63^ Under irradiation by 420 nm (*λ*_ex_ = 427 nm) light, NBF-NO releases NO and a stable green fluorescent product NBF (Fig. 16). In addition, the donor can also be effectively coated by thermally responsive nano-micelles, so that the photochemical and physical properties of the probe can be protected making it suitable for use in complex biological environments.

**Fig. 17:**
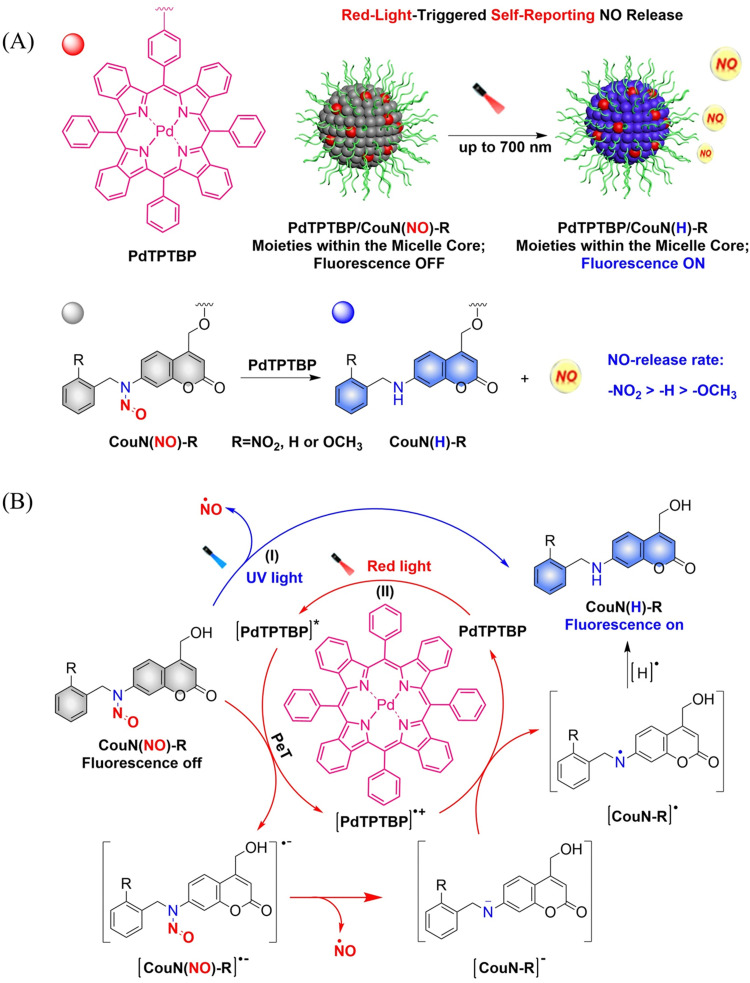
(A) Illustration of red-light-triggered self-reporting NO release. (B) The structure of CouN(NO)-R, the mechanism of NO release. (I) Under UV light irradiation, CouN(NO)-R is directly photolyzed to release NO. (II) Under red-light irradiation, photoredox catalysis occurs facilitated by photosensitizers to release NO.

In order to achieve good imaging characteristics, a red-light mediated photocatalytic NO donor (CouN(NO)-R, R = NO_2_/H/OCH_3_) was developed by the Hu group.^23^ PdTPTBP/CouN(NO)-R is composed of palladium(ii) tetraphenyltetrabenzoporphyrin (PdTPTBP) and *N*-nitrosoamine moieties (CouN(NO)-R). CouN(NO)-R provides quantification of NO concentrations (as a result of the absorbance changes caused by the quantitative conversion from CouN(NO)–NO_2_ to CouN(H)–NO_2_) and reports on NO release (*via* obvious fluorescence changes before and after reaction) through photoredox catalysis (Fig. 17(A)). CouN (NO) has two modes of releasing NO: (i) the donor is activated under 365 nm light irradiation (Coumarin has the maximum absorbance at 328 nm); (ii) in the presence of photosensitizer PdTPTBP, the donor is activated by 630/700 nm red-light (triplet sensitization indirectly activates receptors through a triplet–triplet energy transfer (TTET) process) (Fig. 17(B)). In addition, the authors also explored the effects of different substituents on the NO release rate. When the substituent is an electron withdrawing group, the kinetics of NO release was enhanced, that is, –NO_2_ > –H > –OCH_3_. In biomedical applications, this NO-releasing platform with ciprofloxacin loading can eradicate *Pseudomonas aeruginosa* biofilms *in vitro* and accelerate wound healing *in vivo*.

**Fig. 18:**
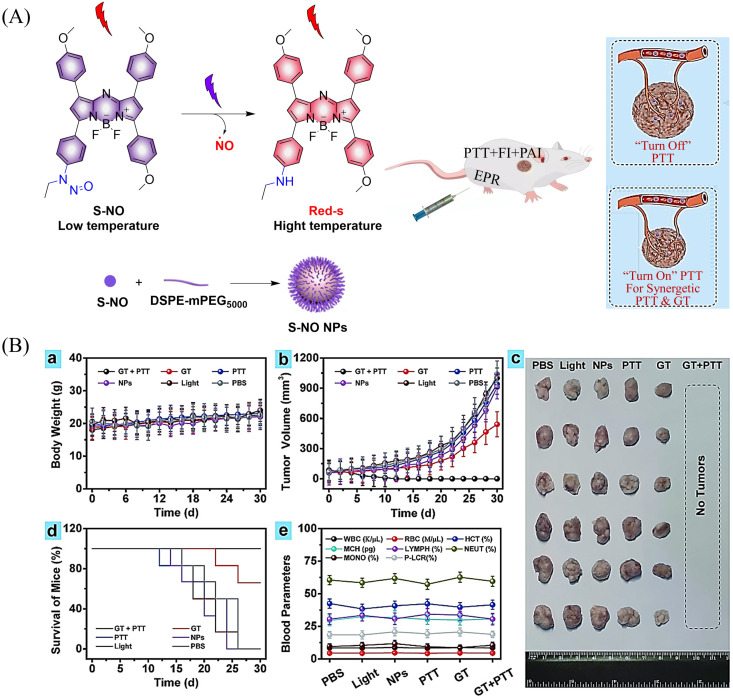
(A) The structure of S-NO, the mechanism of NO release and its fluorescence changes. (B) Assembly of S-NO NPs and synergistic tumor treatment and evaluation of GT and PTT produced by injection into mice. Reproduced from ref. 64 with the permission of Elsevier Ltd, copyright 2021.

Photothermal therapy (PTT), as a tumor treatment strategy, has the advantages of high selectivity, spatiotemporal control, and minimal side effects. Therefore, combining PTT with gas therapy can significantly improve the effect of tumor treatment. The Liu research group have reported a near-infrared photothermal agent (S-NO) for synergetic gaseous therapy (GT) and PTT, which is composed of aryl *N*-nitrosamine and functionalized aza-BODIPY (antenna).^64^ The reason for selecting aza-BODIPY as the fluorophore group is that its conversion from electron withdrawing group (–N–NO) to donating groups (–NH) will lead to significant red-shift and enhanced near-infrared absorption. To improve the water solubility of S-NO, nano particles (S-NONPs) were prepared by assembling with the polymer DSPE-mPEG5000 (Fig. 18(A)). Under NIR-light irradiation, S-NO NPs exhibited excellent NO release capacity and significantly enhanced heat generation. This material contributed to the successful inhibition of tumor growth by synergistic GT and PTT (Fig. 18(B)). This study provides guidance for the construction of dual-effect synergistic tumor treatment platforms.

#### 2.1.2. Nitrobenzene compounds

**Fig. 19:**
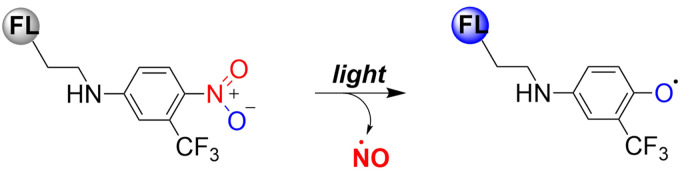
Mechanism of NO release from nitrobenzene compounds.

Previous reports have indicated that nitrobenzene derivatives exhibit a strong capacity to release NO under light activation. A non-planar torsional conformation of substituents relative to aromatic rings affects the ability of nitrobenzene to release NO. It is worth noting that the CF_3_ substituent induces distortion of the geometry of the nitro group under visible light irradiation, and enhances NO release by stabilizing the oxygen free radicals. Specifically, 4-nitro-3-(trifluoromethyl)aniline NO donors, release NO under the action of blue light following nitro-to-nitrite rearrangement (Fig. 19).^65–67^

**Fig. 20:**
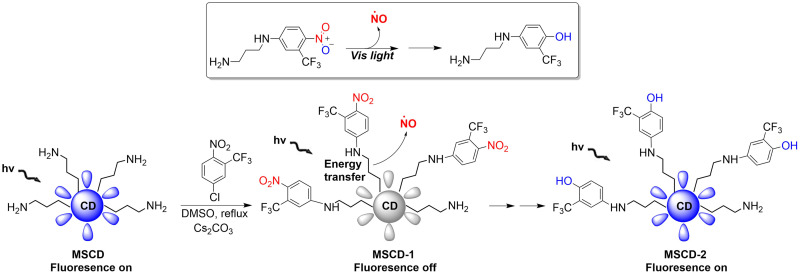
The structure of MSCD, the mechanism of NO release and its fluorescence changes.

Mesoporous silica nanoparticles (SNPs) are extremely effective solid carriers (easy surface functionalization, selective endocytosis by cancer cells, and the release of loaded drugs can effectively lead to apoptosis).^68–71^ The Sortino research group have reported a NO donor with light-controlled multivalent silica nanoparticles as the carrier (Fig. 20).^72^ The nitroaniline based donor was covalently linked to the surface amino group of fluorescent carbon dots by calcination to obtain the hybrid MSCD-1. The fluorescence in MSCD-1 is quenched by effective energy transfer. However, under visible light irradiation, MSCD-1 releases NO, inhibits energy transfer, and leads to fluorescence recovery of the MSCD scaffold. The fluorescence changes before and after reaction can be used to monitor the release of NO. This provides interesting prospects for precise spatiotemporal control of NO release and real-time quantitative biomedical research.

**Fig. 21:**
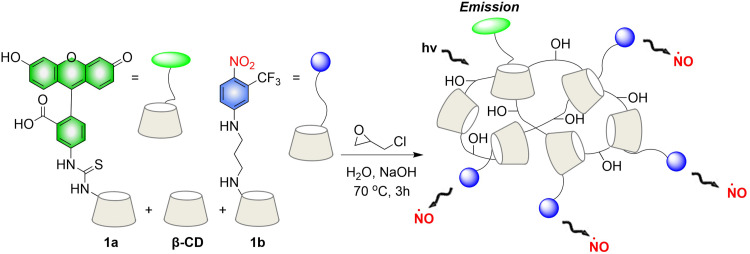
The structure of β-cyclodextrin polymers, the mechanism of NO release and the fluorescence changes.

Crosslinked β-cyclodextrin polymers have been shown to be able to encapsulate visible light activated prodrug molecules in a non-covalent manner and deliver them to cancer cells.^73–75^ But conventional non-covalent polymer carriers do dissociate upon dilution and are weak to environmental changes. To solve these problems, in 2019, the Sortino group reported a new water-soluble β-Cyclodextrin polymer. The polymer contains covalently bound fluorescein isothiocyanate (FITC), β-cyclodextrin and NO donor unit nitroaniline, the two chromophores are activated in parallel under the induction of visible light, resulting in green fluorescence emission suitable for imaging (Fig. 21).^76^ The polymer can be internalized in squamous cancer cells and induces cell mortality on NO photo-decaging.

#### 2.1.3. *N*-Diazeniumdiolate compounds

**Fig. 22:**
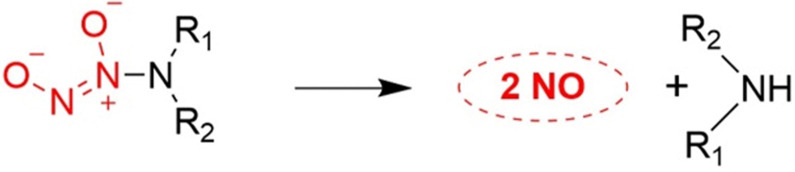
*N*-Diazeniumdiolate compounds release NO.

According to previous research reports, we know that ethylenediamine/NO decomposition can produce diethylamine and NO, which is a very important class of NO donors. Since the simple O^2^-alkylated *N*-diazeniumdiolates are stable, they hydrolyze slowly even in acidic solutions. This provides the design of O^2^-substituted *N*-diazeniumdiolates which would themselves react under a variety of conditions to regenerate unsubstituted diazeniumdiolate, thus initiating its dissociation to produce NO as described above (Fig. 22).^77^

**Fig. 23:**
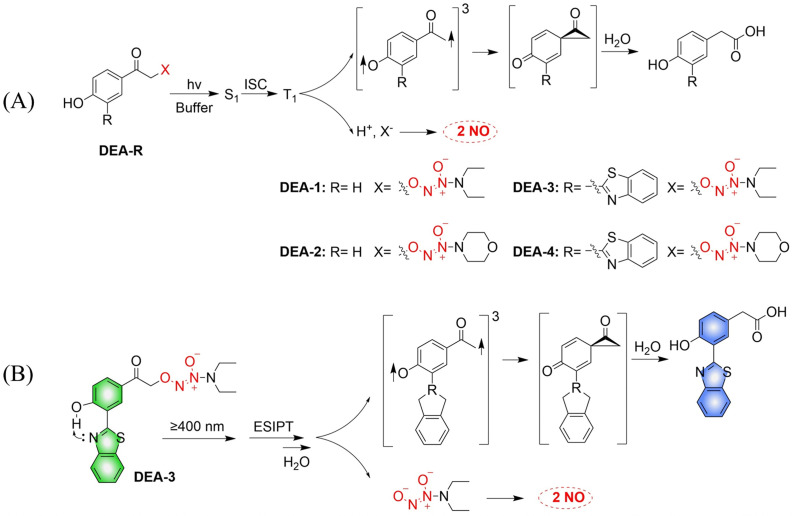
(A) The rational design of O^2^-(4-hydroxyphenacyl) diazeniumdiolates DEA-1 and DEA-2 as well as O^2^-(3-(benzothiazole-2-yl)-4-hydroxyphenacyl) diazeniumdiolates DEA-3 and DEA-4, together with the proposed mechanism underlying the photorelease of NO. (B) The structure of DEA-3, the mechanism of NO release and the fluorescence changes.

The Huang research group developed a series of NO donors (ethylenediamines).^78^ Since the photolysis wavelength of 4-hydroxyphenacyl is lower than 400 nm and DEA-1/2 are non-fluorescent, the author modified the system by introducing benzothiazol-2-yl in the position *ortho* to the hydroxyl on the benzene ring of DEA-1/2 to prepare DEA-3/4 (Fig. 23(A)). The advantages of this system are: on the one hand, DEA3/4 can undergo photolysis with irradiation of ≥410 nm visible-light; and on the other hand, excited-state intramolecular transfer (ESIPT), that is, the proton of the hydroxyl group transfers to the nitrogen atom of the benzothiazole, can help deprotonation of the hydroxyl group and accelerate the photodegradation rate of DEA3/4, thus resulting in the rapid release of NO. The DEA-3 process is accompanied by obvious fluorescence changes (from green to blue) (Fig. 23(B)).

### 2.2. Enzyme activated NO release

**Fig. 24:**
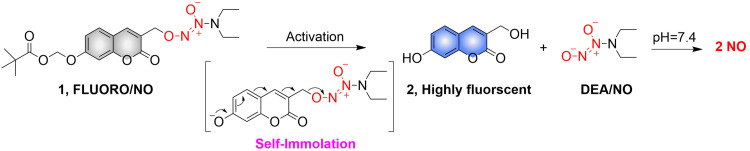
The structure of FLUORO/NO, the mechanism of NO release and its fluorescence changes.

Biocatalysis can provide the precise selectivity of enzymes to achieve the release of required drugs.^79–81^ The Chakrapani research group developed an esterase activated *N*-diazeniumdiolate NO donor (FLUORO/NO).^82^ When the hydroxyl group of coumarin is linked with appropriate activating groups the fluorescence of the FLUORO/NO is significantly diminished. Subsequently, with the activation of esterase, FLUORO/NO undergoes a self-immolation reaction, releasing DEA, thus significantly enhancing the fluorescence of coumarin (Fig. 24).

**Fig. 25:**
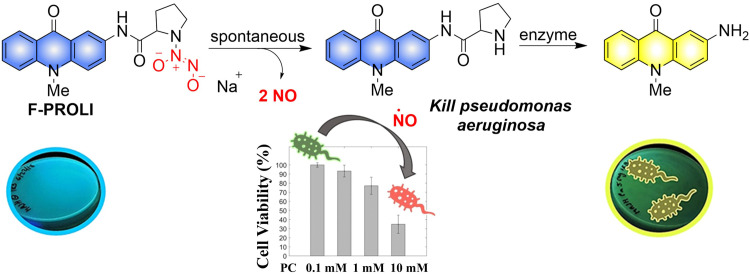
The structure of donor, the mechanism of NO release and fluorescence changes. Reproduced from ref. 83 with the permission of the Royal Society of Chemistry, copyright 2019.

Given the prevalence of multidrug resistant bacteria, improved detection and treatment methods are urgently required. NO is a known effective antibacterial agent, but because of its gaseous nature and high reactivity, it is difficult to integrate into a stable antibacterial system. Fortunately, the emergence of multifunctional materials has solved this problem. The Reynolds group have reported a small molecule dual function NO donor (F-PROLI, bacterial indicator and antibacterial agent).^83^ Under UV irradiation, proline based diazeniumdiolate salts spontaneously release NO to kill bacteria. Subsequently, the bacterial enzyme prolyl aminopeptidase in *Pseudomonas aeruginosa* will cleave the proline group from the fluorescent aminoacridone/proline compound, resulting in a fluorescence change from blue to yellow. This material (F-PROLI) can both detect and kill *Pseudomonas aeruginosa* a deadly and multi-drug resistant bacterial strain (Fig. 25).

### 2.3. Reactive oxygen species triggered NO release

**Fig. 26:**
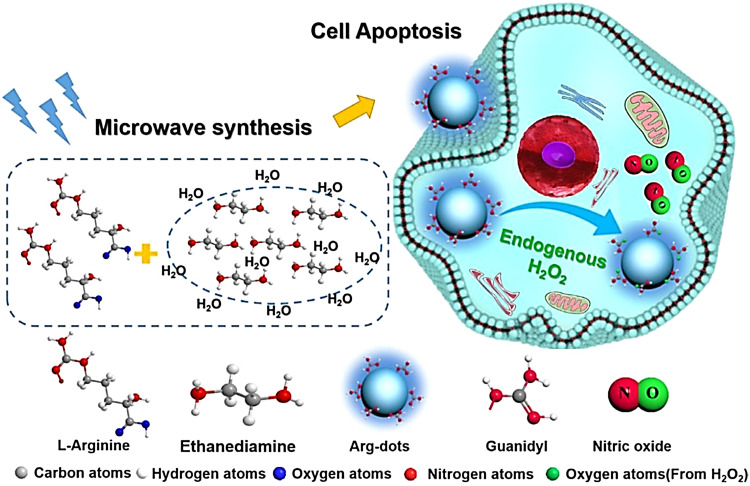
Schematic of the synthesis of Arg-dots and their NO release process in tumor cells. Reproduced from ref. 86 with the permission of Elsevier Ltd, copyright 2021.

Arginine is a natural endogenous NO donor, which can release a large amount of NO in cells through the catalysis of nitric oxide synthase.^29,84,85^ The Zhang group reported fluorescent carbon nanodots based on l-Arginine (Arg-dots), to enhance the treatment of cancer with NO gas. The nanoplatform contains a hydrogen peroxide donor to increase the hydrogen peroxide concentration at the tumor site, thus overcoming the low concentration hydrogen peroxide environment that restricts NO production from arginine (Fig. 26).^86^ After being endocytosed by cancer cells, the Arg-dots can generate a large amount of intratumoral NO to kill cancer cells. As such, without any additional external intervention (in the context of H_2_O_2_-enriched tumor microenvironments), Arg-dots autonomously release NO at a twofold higher rate when compared to equimolar l-Arg. When combined with traditional chemotherapeutic drugs (adriamycin), Arg-dots greatly improve the treatment effect toward cancer. In addition, the size of Arg dots is very small (about 2.5 nm in diameter), thus they can effectively penetrate deep tumors and provide enhanced antitumor activity. In addition, Arg-dots can be removed by kidney filtration, thereby reducing the burden on the human body in the process of cancer treatment.

### 2.4. Thiol triggered NO release

**Fig. 27:**
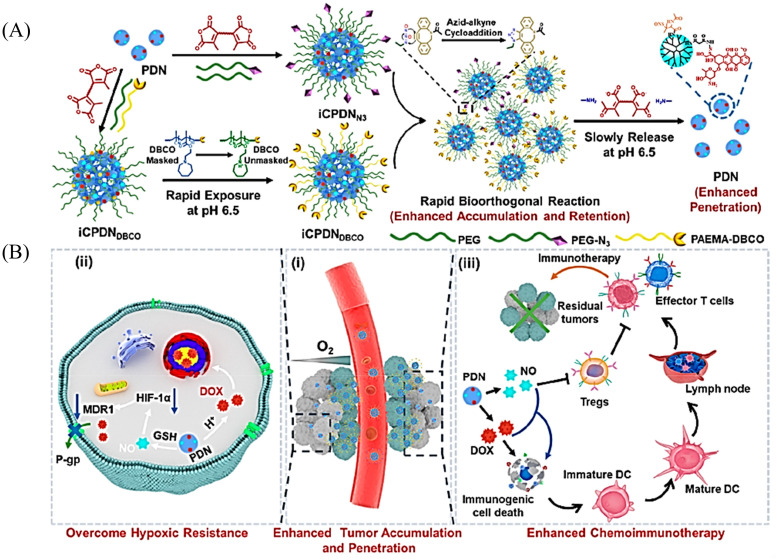
Schematic of *in situ* size transformation cluster nanosystem mediated by tumor acidity and bioorthogonal chemistry to overcome hypoxic resistance and enhance chemical immunotherapy. (A) Schematic diagram of preparation of iCPDN_N3_ and iCPDN_DBCO_, forming large particle size aggregates by efficient biological orthogonal click reaction under acidic environment, and then slowly releasing small particle size PDN. (B) (i) *In situ* size transformation cluster nanosystem enhanced tumor accumulation, retention, and penetration. (ii) NO overcomes hypoxia resistance by down-regulating HIF-1α level and enhances the chemotherapy effect of DOX. (iii) NO and DOX can induce stronger immunogenic cell death and activate anti-tumor immune responses. Reproduced from ref. 91 with the permission of the American Chemical Society, copyright 2022.

It has been reported that S-NO can be cleaved to release NO under specific triggering conditions (the mechanism of NO release is similar to that of *N*-nitroso compounds, Fig. 2).^87–90^ The Yuan group recently reported a tumor-acidity and bioorthogonal chemistry-mediated *in situ* size transforming cluster nanosystem (PAEMA-DOX/NO(PDN)) (Fig. 27(A)).^91^ The system uses PAEMA and high-efficiency bioorthogonal click chemistry to form large-scale aggregates in tumor tissues which enhances the accumulation in tumor tissues and the retention of DOX and NO. Subsequently, in the acidic microenvironment of the tumor, iCPDN(PAEMA-DOX/NO(PDN)) is cleaved, and the aggregates are slowly decomposed into ultra-small nanoparticles PDN with better tumor penetration. That is, DOX (pH responsive release) and NO (GSH responsive release) are delivered to hypoxic tumor tissues (Fig. 27(B)). Such a synergistic treatment of NO and DOX may be an effective method to combat hypoxia-induced chemical tolerance and enhance anti-tumor immune response.

### 2.5. Redox activated NO release

**Fig. 28:**
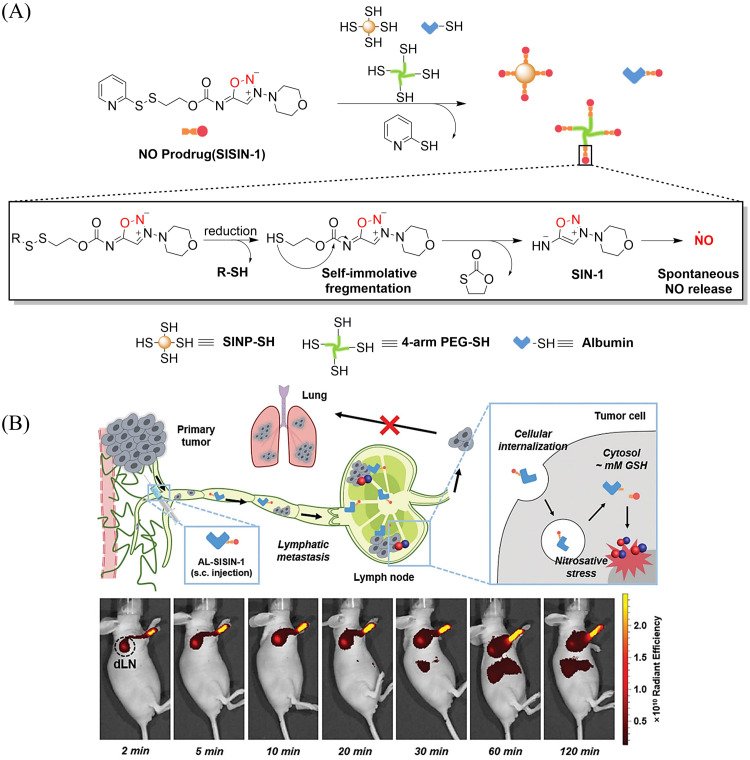
(A) The structure of SISIN-1 and mechanism of NO release (B) SISIN-1 combines with albumin to form AL-SISIN-1, which delivers drugs to tumor draining lymph nodes and inhibits cancer cell metastasis. Reproduced from ref. 92 with the permission of Wiley-VCH GmbH, copyright 2022.

Tumor-draining lymph nodes (TDLN) contain various types of immune cells, such as natural killer cells, T cells, antigen-presenting cells and B cells, which play a key role in tumor metastasis. Therefore, lymphatic targeted anti metastasis therapy is particularly important. The Kim group developed a redox triggered NO prodrug (SISIN-1) that can target TDLE (Fig. 28(A)).^92^ Since albumin has the ability to enhance lymphatic drainage and endocytosis in cancer cells,^93,94^ AL-SISIN-1 formed by binding SISIN-1 to albumin is able to efficiently deliver NO to TDLE, thereby inhibiting tumor metastasis. The disulfide bond of SISIN-1 is reduced to a free thiol that initiates intramolecular nucleophilic attack and cleaves the carbamate linked to SIN-1, thereby releasing free SIN-1. Under physiological conditions, free SIN-1 released by SISIN-1 will spontaneously decompose and release NO. The blue shift in absorption of SISIN-1 can be used as a marker for monitoring NO release (Fig. 28(B)). This new prodrug contributes to the development of on-demand NO delivery systems for anti-metastasis and other treatments.

### 2.6. One-electron reduction NO release

**Fig. 29:**
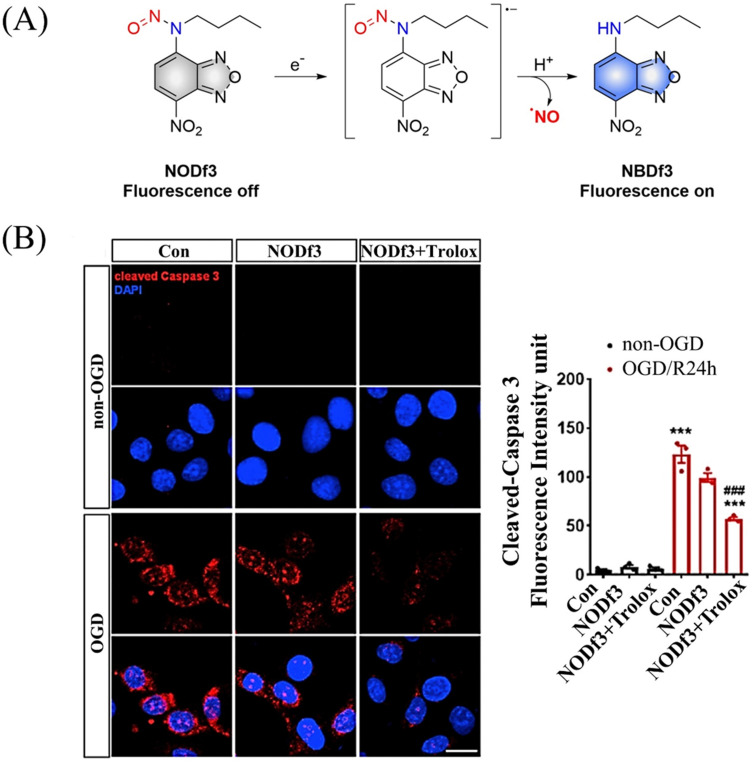
(A) The structure of NODf3, the mechanism of NO release and its fluorescence changes. (B) NODf3 combined with Trolox protects HUVECs from OGD-induced apoptosis. Reproduced from ref. 36 with the permission of Elsevier Inc., copyright 2021.

Under light, photoexcitation, *N*-nitroso undergoes photolysis to release NO, and its structure changes from an electron withdrawing group –NNO to electron-rich –NH. However, when the fluorophore framework is a strong electron withdrawing group, it can replace the photolysis pathway of *N*-nitroso, that is, to release NO through a reduction-triggered protonation cleavage pathway. The Yang research group reported a series of one-electron reduction triggered NO donors (NODf3) (Fig. 29(A)).^36^ NODf3 releases NO under the trigger of one-electron reduction. Since this triggering mechanism does not depend on the reduction ability of thiols, it does not affect the redox level of cells. In addition, it was found that NODf3 has good biocompatibility and efficient reduction rate and exhibits therapeutic and cytoprotective effects in oxygen and glucose deprivation (OGD) models (Fig. 29(B)).

## 3. Carbon monoxide

Carbon monoxide (CO) is a colorless, odorless inert gas that has the longest lifespan among the three main known gaseous signaling molecules (NO, CO, and H_2_S).^95^ However, despite its ability to produce acute toxicity when bound in significant quantities to hemoglobin, resulting in rapid fatality,^96^ CO also plays a vital role in medical treatments.^16^ This includes inducing vasodilation,^15^ mitigating acute kidney injury,^97^ protecting neurotransmitters,^98^ displaying anti-inflammatory and antioxidative properties.^99^ Furthermore, CO can disrupt cytochrome *c* oxidase in mitochondria, hindering oxidative phosphorylation, effectively inhibiting the growth and proliferation of cancer cells.^10,100^

### 3.1. Photo controlled CO release

#### 3.1.1. Metal–carbonyl complexes

**Fig. 30:**
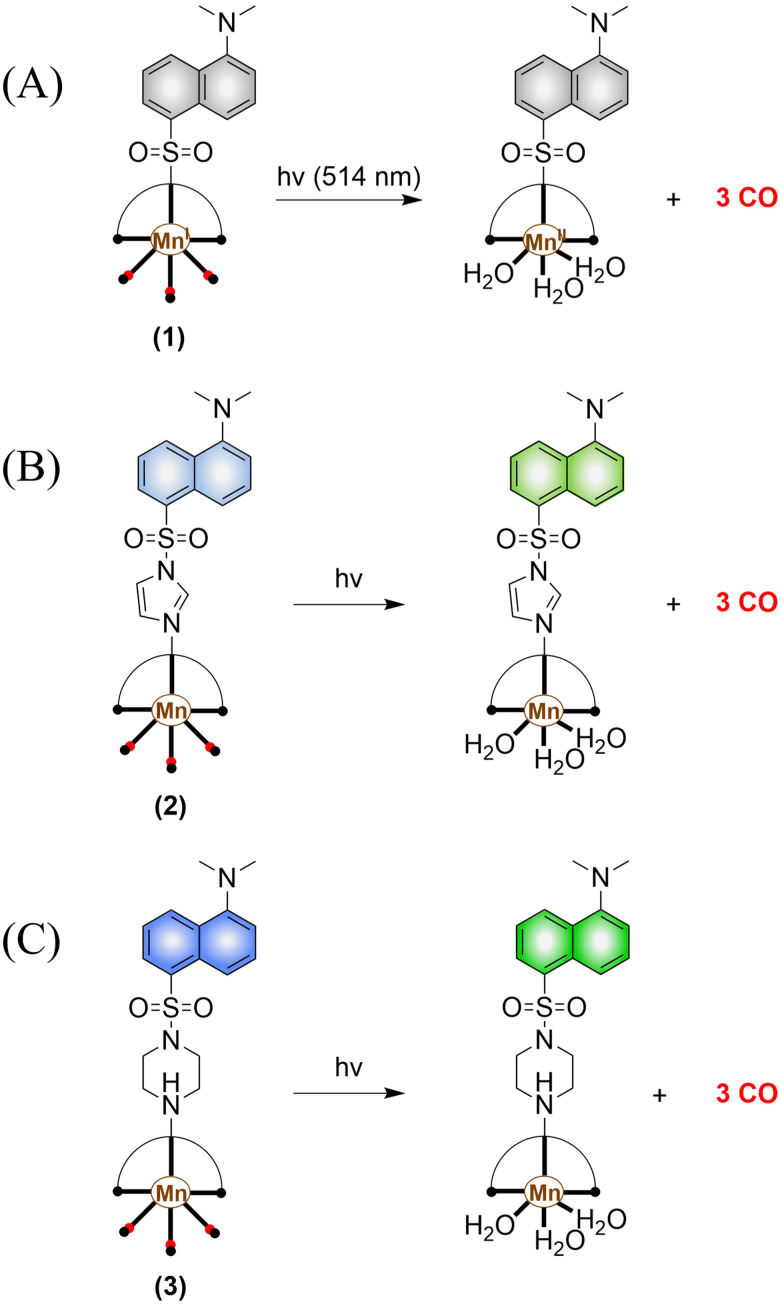
(A)–(C) The structure of Mn-carbonyl complexs, the mechanism of CO release and fluorescence changes.

The most widely used CO-releasing molecules (CORMs) are metal carbonyl compounds, including ruthenium-based carbonyl complexes and manganese-based carbonyl complexes. All these donors spontaneously release CO through ligand exchange in an aqueous environment, but can not monitor the CO release in cellular environments. The Schiller group reported a CO donor with fluorescence activity [Mn(CO)_3_(L)](CF_3_SO_3_) (L = ligand 5-(dimethylamino)-*N*,*N*-bis(pyridine-2-ylmethyl) naphthalene-1-sulfonamide) (Fig. 30(A)).^101^ [Mn(CO)_3_(L)](CF_3_SO_3_) behaves as a logic gate *via* co-registering the inputs of light irradiation and peroxide into concomitant outputs of fluorescence and CO, thus achieving visual tracking of CO release. While the direct binding of the N atom to the Mn(i) center in this photoCORM results in almost complete quenching of the fluorescence, thus making it difficult to track and locate the pro-drug in cells. To track the cellular absorption of the prodrug, the Mascharak research group designed a photoactive manganese carbonyl complex derived from dansylimidazole (Imdansyl, a fluorescent ligand dansylimidazole), namely, [Mn(Imdansyl)(CO)_3_(phen)](CF_3_SO_3_) (Fig. 30(B)). The imidazole linker in [Mn(Imdansyl)(CO)_3_(phen)](CF_3_SO_3_) separates the fluorophore from the Mn(i) center, which results in moderate luminescence. Thus, visual tracking of prodrug uptake by cells was realized.^102^ As such if the fluorescent ligand is not directly attached to the Mn center of the photoCORM (as shown in Fig. 30(B) and (C)), then the fluorescence of the remote fluorophore will not be quenched and the photoCORM can be tracked within cellular targets. To achieve optimal results, the authors also developed [Mn(CO)_3_(phen)(Pipdansyl)](CF_3_SO_3_), with piperazine as the linker. Piperazine can coordinate to the Mn core, and maintains a bright fluorophore (Fig. 30(C)).^103^ As expected, complex 3 exhibits bright fluorescence positioning before and after CO release, thus enabling more accurate monitoring of CO release.

**Fig. 31:**
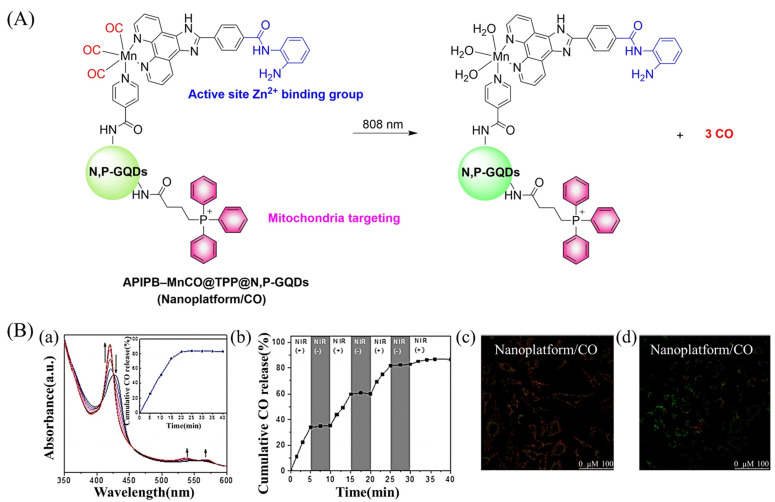
(A) The structure of APIPB–MnCO@TPP@N,P-GQDs, the mechanism of CO release and its fluorescence changes. (B) (a) The absorbance change of hemoglobin in the presence of Nanoplatform/CO irradiated by 808 nm laser (800 mW cm^−2^), with the inset showing the CO release profiles. (b) The NIR controllability of Nanoplatform/CO for CO release upon the on/off switching of an 808 nm laser. Fluorescence detection of mouse mitochondrial membrane potential, Nanoplatform/CO treated HeLa cells (c) under dark environment and (d) under 808 nm laser irradiation. Reproduced from ref. 109 with the permission of Elsevier Inc., copyright 2022.

Since mitochondria regulate a variety of important functions in cell physiology, there is a clinical requirement for mitochondria-targeted therapy.^104–108^ The Liu research group have developed a multifunctional nano-platform for the mitochondria-targeted therapy of cancer (APIPB-MnCO@TPP@N,P-GQDs).^109^ This platform contains the covalently attached histone deacetylase inhibitors, CO donor MnBr(CO)_3_, and mitochondria-targeting groups (triphenylphosphine, TPP) to N,P-doped GQDs fluorescent carriers. Under 808 nm NIR light irradiation, this platform undergoes charge-transfer transitions and releases CO and generates significantly enhanced fluorescence (Fig. 31(A) and (B)). As expected, the combination of GT and histone deacetylase inhibition improves the anticancer activity.

**Fig. 32:**
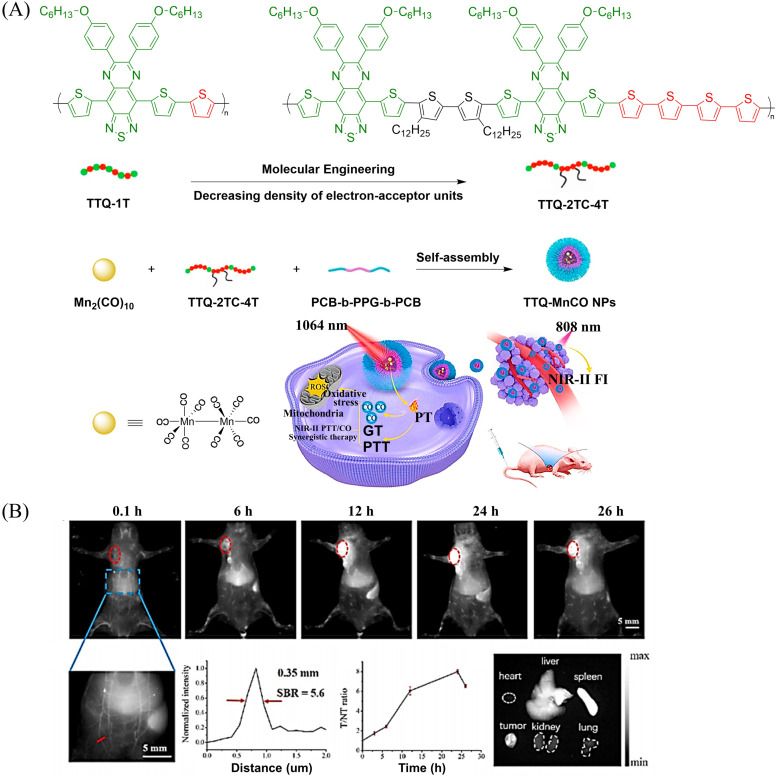
(A) Illustration of red-light-triggered self-reporting CO release from micellar nanoparticles containing TTQ-2TC-4T, PCB-*b*-PPG-*b*-PCB and CO-releasing Mn_2_(CO)_10_ moieties within the cores. (B) Infrared photothermal imaging of MCF-7 tumor mice triggered by 808 nm light and its therapeutic effect on cancer. Reproduced from ref. 110 with the permission of Elsevier Ltd, copyright 2022.

Other donors with excellent performance have also been reported. The Fan research group have designed nanoparticles (TTQ-MnCO NPs) with efficient NIR-II FI and NIR-II PTT/GT.^110^ Since weaker electron withdrawing groups are beneficial to reduce non-radiative attenuation and increase the NIR-II fluorescence intensity, the authors engineered the conjugated polymer by reducing the density of withdrawing groups (TTQ) on the main chain of the conjugated polymer. Moreover, a long alkyl side chain modified dithiophene (2TC) was introduced as an electron donor group, to improve the solubility in organic solvents. TTQ-2TC-4T and thermal responsive CO donor (Mn_2_(CO)_10_) were loaded into an amphiphilic block copolymer (PCB-*b*-PPG-*b*-PCB) to obtain TTQ-MnCO NPs (Fig. 32(A)). Under 808 nm NIR laser light, TTQ-MnCO NPs turned on the NIR-II FI. Under the irradiation of 1064 nm laser, TTQ-MnCO NPs could generate heat to trigger the effective production of toxic CO, realizing the synergistic treatment by NIR-II PTT/GT, which represents an innovative development in synergistic cancer therapy (Fig. 32(B)). This study provides a new method for optimizing the fluorescence and photothermal properties of NIR-II conjugated polymers.

#### 3.1.2. 3-Hydroxyflavone and 3-hydroxyquinolone derivatives

**Fig. 33:**
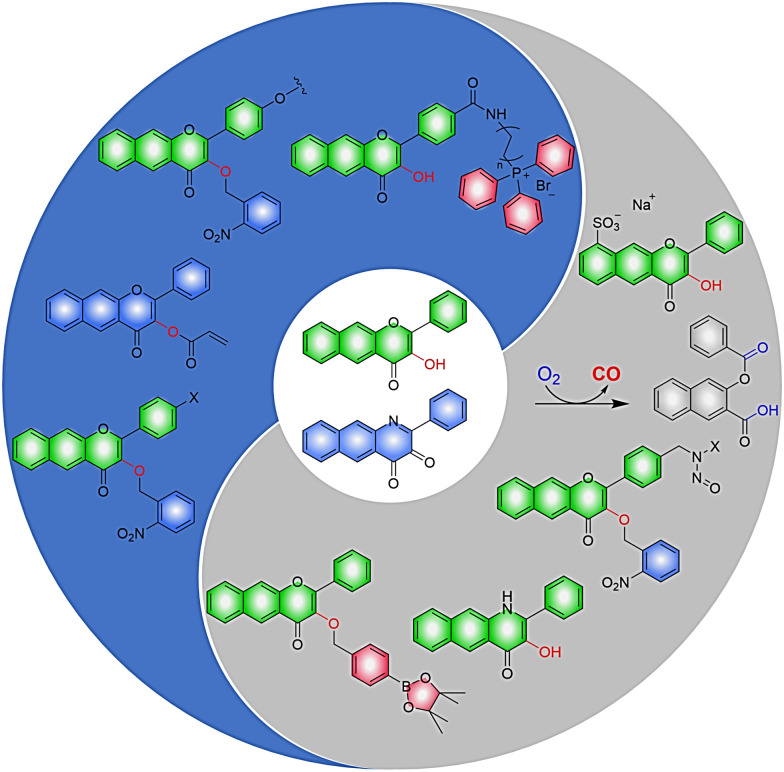
CORMs based on 3-hydroxyflavone and 3-hydroxyquinolone.

As a typical class of ESIPT molecule,^111^ structurally adjustable 3-hydroxyflavone and 3-hydroxyquinolone derivatives can not only release CO under visible light irradiation,^112–114^ but can also be used to develop probes for biological thiols.^115,116^ Inspired by this concept, Berreau, Tang, Hu *et al.* have developed a series of metal-free CO donors (CORMs) based on structurally adjustable flavonols and quinolones (Fig. 33). With this donor the aromatic framework has been extended by adding an additional ring, resulting in the absorption of visible-light.^117–123^ Increasing research has indicated that the advantages of CORMs are: (i) sensing of the cellular environment before the release of CO, thereby regulating the redox state. (ii) Evaluation of the effects of cytosol and mitochondria on the oxidative stress of cells. (iii) Exhibit high affinity with protein carriers and can deliver a specific amount of CO. (iv) The effect of intracellular/extracellular CO production on disease treatment can be evaluated, setting a precedent for intracellular and extracellular combined therapy. (v) Provide strong evidence for CO induced vasodilation. (vi) Negligible cytotoxicity and high anti-inflammatory effect. (vii) light controlled co-release of NO and CO to achieve synergistic treatment of cancer. (viii) Fluorescence changes provide visual monitoring of CO release. Consequently, such fluorescent donors of CO have become the focus of both biological tracking and drug delivery.

**Fig. 34:**
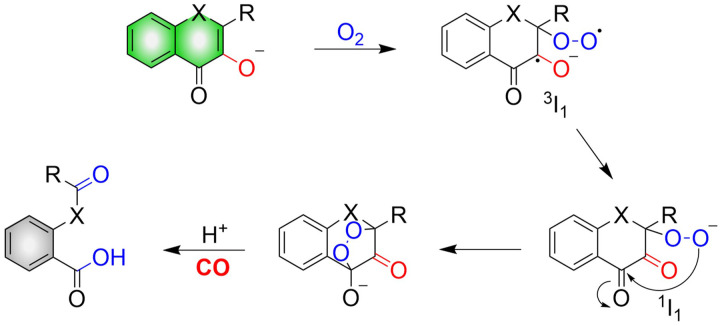
Mechanism of CO release from flavonol compounds.

The mechanism of CO release from such donors requires: firstly, in the presence of both light and O_2_, the hydroxyl group of flavonols undergoes a one-electron transfer to O_2_, producing a triplet superoxide ^3^I_1_. Subsequently, an internal electron transfer (ICT) leads to the production of the closed shell-layer singlet oxygen ^1^I_1_. Finally, β-C is attacked by the terminal oxygen of the superoxide to form an endoperoxide to release CO (Fig. 34).^114^

**Fig. 35:**
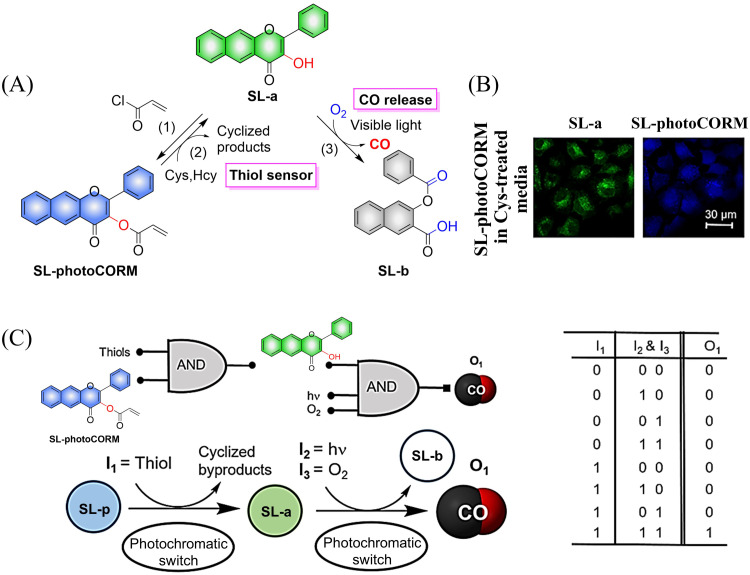
(A) The structure of SL-photoCORM (SL-p), the mechanism of CO release and its fluorescence changes. (B) SL-p in A549 cells is converted into the confocal image of SL-a under the action of mercaptan. (C) The process behave like an AND logic gate. Parts (B) and (C) are reproduced from ref. 119 with the permission of the American Chemical Society, copyright 2017.

Representative work is as follows: inspired by the work of Liu *et al.*,^115^ the Berreau group expanded the 3-hydroxy-flavonol structure by adding an acryloyl appendage to the 3-OH site (reaction 1), thus developing a fluorescent compound (SL-photoCORM) that combines thiol-detection with light-controlled CO release.^119^ In cells, SL-photoCORM firstly undergoes a Michael addition reaction of the acroyl group with Cys/Hcy resulting in enhanced fluorescence and monitoring of the cell redox state. The system then releases CO when triggered by visible light and O_2_ (Fig. 35(A) and (B)). The process follows the “AND” logic gate (Fig. 35(C)). SL-photoCORM combines the detection of redox biomarkers with the release of small molecules, realizing the dual function of a single probe.

**Fig. 36:**
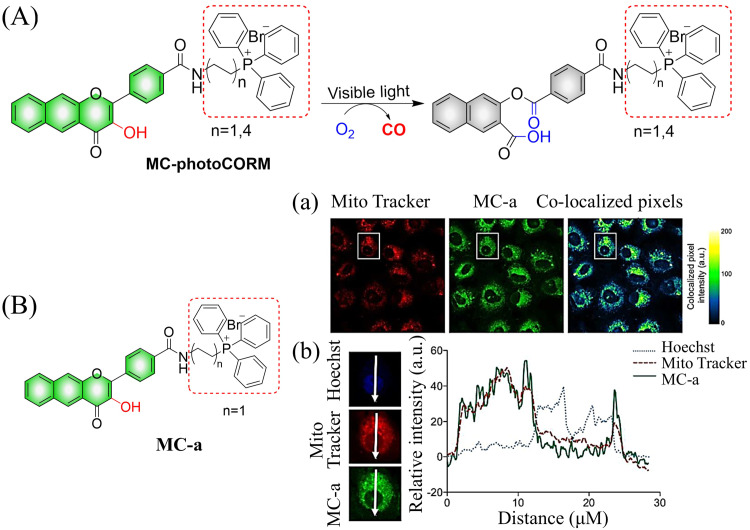
(A) The structure of MC-photoCORM, the mechanism of CO release and its fluorescence changes. (B) MC-a (*n* = 1) and MTR intensity distribution strongly indicates that MC-a is located in the mitochondria. Reproduced from ref. 120 with the permission of the American Chemical Society, copyright 2018.

The same group reported another CO donor (MC-photoCORM) for mitochondrial-targeted release of CO (Fig. 36(A)).^120^ Owing to the mitochondrial targeting function of triphenylphosphine and flavonol, this donor can easily be absorbed by cells and release CO *in situ* (Fig. 36(B)).

**Fig. 37:**
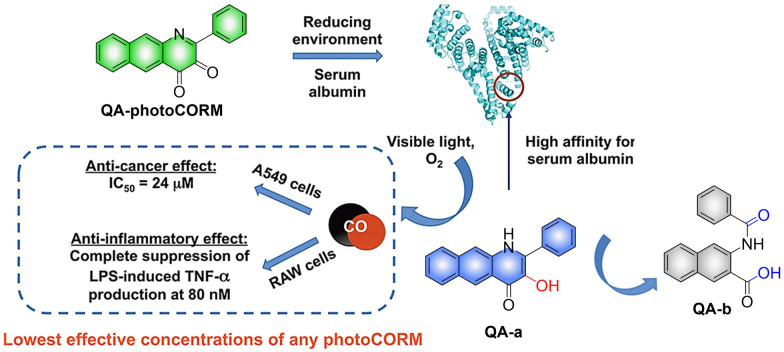
The structure of QA-photoCORM, the mechanism of CO release and its fluorescence changes. Reproduced from ref. 121 with permission of the American Chemical Society, copyright 2018.

In addition to 3-hydroxyflavone, fluorescent donors based on quinolone have been widely developed for CO release. For example, the Berreau group have reported a 3-hydroxybenzo[*g*]quinolone framework CO donor (QA-photoCORM).^121^ QA-photoCORM can be tracked prior to CO release using fluorescence microscopy and produces a nontoxic byproducts following CO release. In addition, the strong noncovalent affinity of QA-photoCORM to albumin enables use of an albumin: QA-photoCORM complex for targeted CO delivery to cancer cells. The cytotoxicity IC_50_ value generated by this method is one of the lowest reported values for delivering CO to cancer cells through photoCORM to date (Fig. 37). QA-photoCORM complex is also the first CO delivery system to produce significant anti-inflammatory effects at nanomolar photoCORM concentrations.

**Fig. 38:**
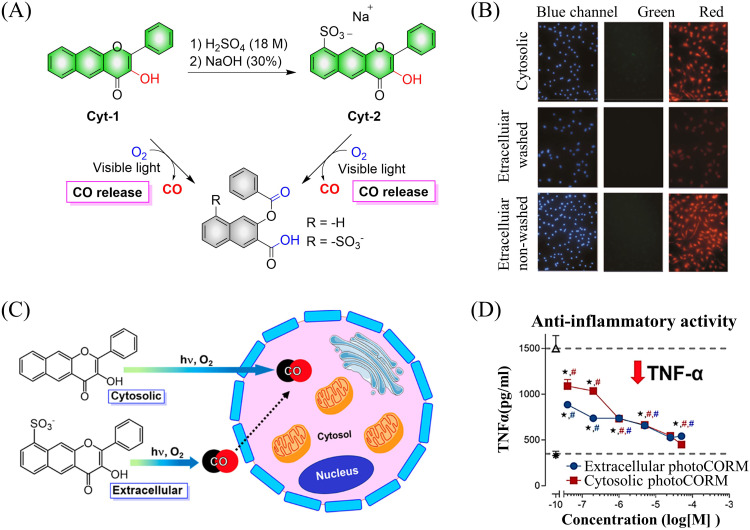
(A) The structure of cytosolic/extracellular-photoCORM, the mechanism of CO release and its fluorescence changes. (B) Fluorescence detection of CO release from Cyt-1 and Cyt-2 (50 μM) using a Nile red-based CO sensor (1-Ac) in RAW 264.7 cells. Green channel: detection of fluorescence emission by cytosolic/extracellular. Red channel: detection of CO sensor. Size of bar = 40 μm. (C) and (D) extracellular administration shows significant anti-inflammatory effect. Reproduced from ref. 123 with the permission of the American Chemical Society, copyright 2019.

Subsequently, the Berreau group evaluated the impact of intracellular and extracellular CO production on disease treatment.^123^ The research indicated that the introduction of sulfonate groups to the donors changed the ability of cells to absorb the donors, and did not have any effect on the optical properties of the 3-hydroxyflavone derivatives (Fig. 38(A) and (B)). In addition, extracellular administration also generated significant anti-inflammatory efficiency (Fig. 38(C) and (D)).

**Fig. 39:**
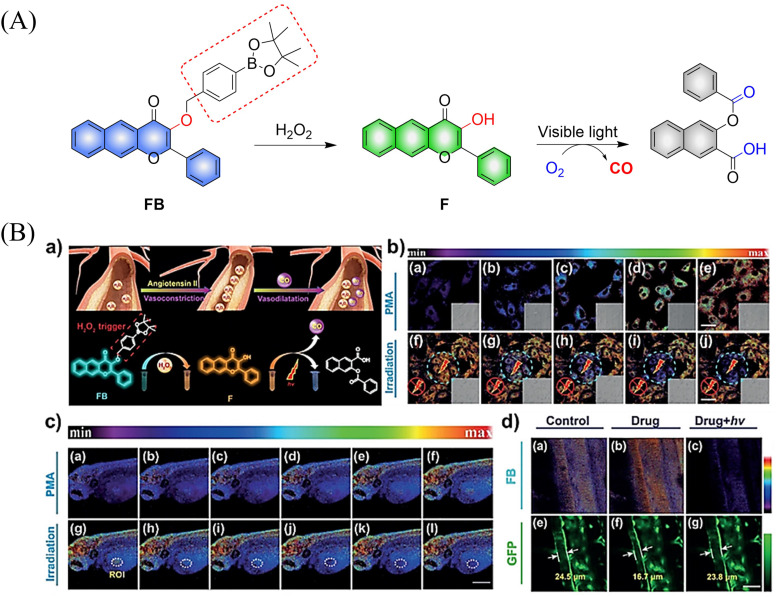
(A) The structure of FB, the mechanism of CO release and its fluorescence changes. (B) (a) The structure and sensing mechanism of FB toward H_2_O_2_-activated and CO photo-releaser. (b) The H_2_O_2_ mapping and CO releasing *in vitro*. (c) The H_2_O_2_ mapping and CO-release *in vivo*. (d) The vasodilatation effect of CO and angiotensin type 2 induced H_2_O_2_ fluctuation. Reproduced from ref. 124 with the permission of Wiley-VCH Verlag GmbH & Co. KGaA, Weinheim, copyright 2018.

Using a similar mechanism, the Tang research group developed a two-photon H_2_O_2_ activated CO donor (FB).^124^ FB is composed of a structurally extended 3-hydroxyflavone (F) combined with a boronate ester group that specifically recognizes ROS. Under the action of ROS, FB generates F which undergoes ESIPT, subsequently F releases CO under light irradiation (Fig. 39(A)). In addition, FB also provides evidence for oxidative stress related to H_2_O_2_ after administration of angiotensin type 2. Therefore, the donor is suitable for the early warning of oxidative stress and light-controlled release of CO (Fig. 39(B)).

**Fig. 40:**
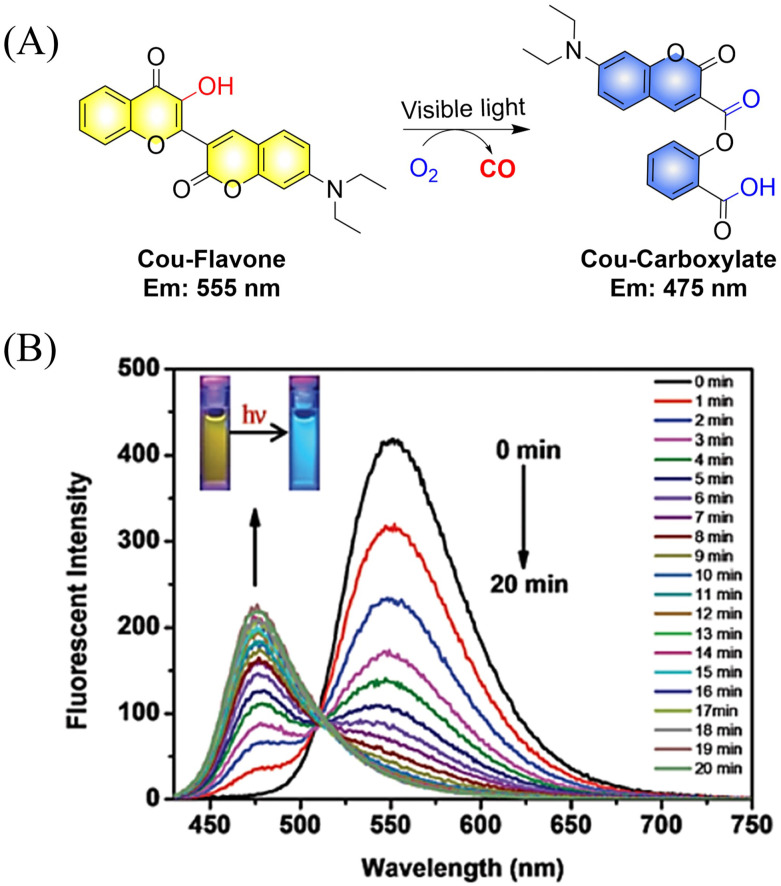
(A) The structure of Cou-Flavone, the mechanism of CO release and its fluorescence changes. (B) The capacity of Cou-Flavone for CO-release was investigated in HEPES buffer (10 mM, pH 7.4, with 10% DMSO, v/v) at room temperature under light irradiation (460 nm, 7 W cm^2^). Typical ratiometric fluorescence changes were observed before and after irradiation. Reproduced from ref. 125 with the permission of the Royal Society of Chemistry, copyright 2019.

The Feng group have made great effort to develop CO donors.^125^ For example, the group reported a visible-light triggered ratiometric CO donor (Cou-Flavone) based on the coumarin-flavonol structure (Fig. 40(A)). Under visible-light irradiation, Cou-Flavone can not only release CO, but also exhibits obvious ratiometric fluorescence changes (Fig. 40(B)). Cou-Flavone can be used to monitor the release of CO by changes in the emission spectra of two fluorophores, therefore making the results more reliable and accurate.

**Fig. 41:**
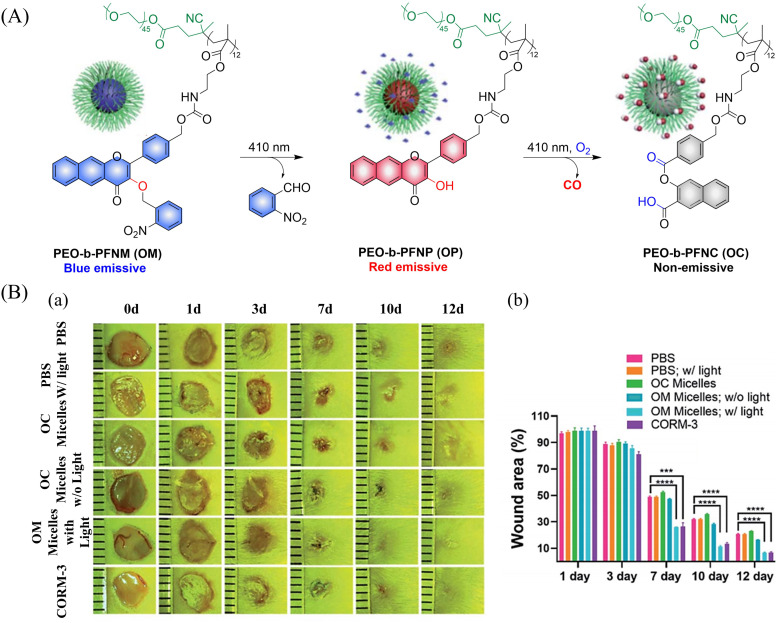
(A) The structure of PEO-*b*-PFNM, the mechanism of CO release and fluorescence changes. (B) Comparison of the treatment effect of PBS, OM and OC micelles on skin wounds. Reproduced from ref. 130 with the permission of the Royal Society of Chemistry, copyright 2020.

In addition CO exhibits excellent use and a therapeutic anti-inflammatory,^126^ skin trauma,^127^ immune imbalance^128^ and oxidative stress^129^ treatment. The group of Hu proposed a direct polymerization strategy for CO donors based on 3-hydroxyflavone (3-HF) derivatives.^130^ CO-releasing polymers were self-assembled into micelles (PEO-*b*-PFNM) using PEG, which improved the water solubility of the extended 3-HF (Fig. 41(A)). Under 410 nm visible-light irradiation, the *o*-nitrobenzyl group was cleaved, and the characteristics of 3-HF derivative were restored. Then in the presence of O_2_, PEO-*b*-PFNM when irradiated with 410 nm light, the 3-HF derivative released CO. The whole process exhibits fluorescence changes from blue to red and then to colorless, facilitating the monitoring of CO release *in vivo* and *in vitro*. Benefiting from these excellent properties, this donor exhibited excellent therapeutic anti-inflammatory and skin wound healing effects (Fig. 41(B)).

**Fig. 42:**
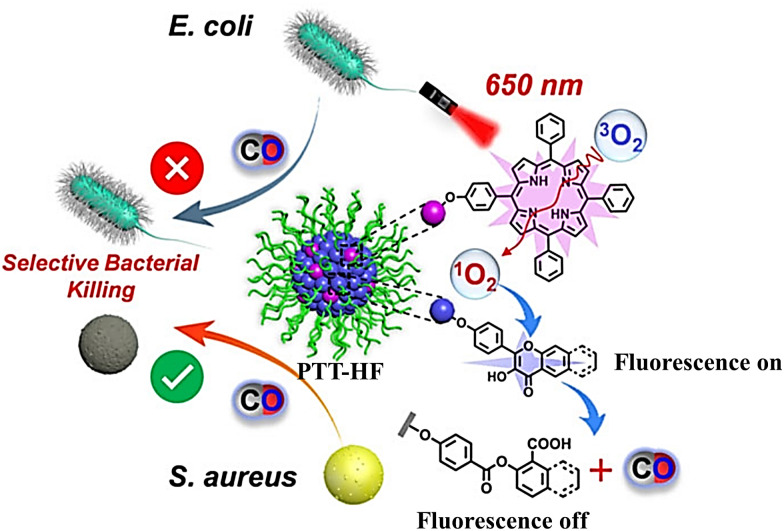
The structure of PTT-HF, the mechanism of CO release and its fluorescence changes. Reproduced from ref. 24 with the permission of Wiley-VCH GmbH, copyright 2021.

Subsequently, the same group have developed metal-free CO release micelle, *via* a photooxidation mechanism to activate flavonol conjugated derivatives to release CO.^24^ Under 650 nm light excitation, tetraphenylporphyrin photosensitizer converts ^3^O_2_ into ^1^O_2_. Then, ^1^O_2_ oxidizes the conjugated derivatives of flavonol, thereby triggering the release of CO. In addition, the micelles exhibit specific antibacterial efficacy toward methicillin-resistant *S. aureus* (MRSA), but not *Escherichia coli*, so they can eradicate MRSA pathogens and accelerate the healing of MRSA infected wounds (Fig. 42).

**Fig. 43:**
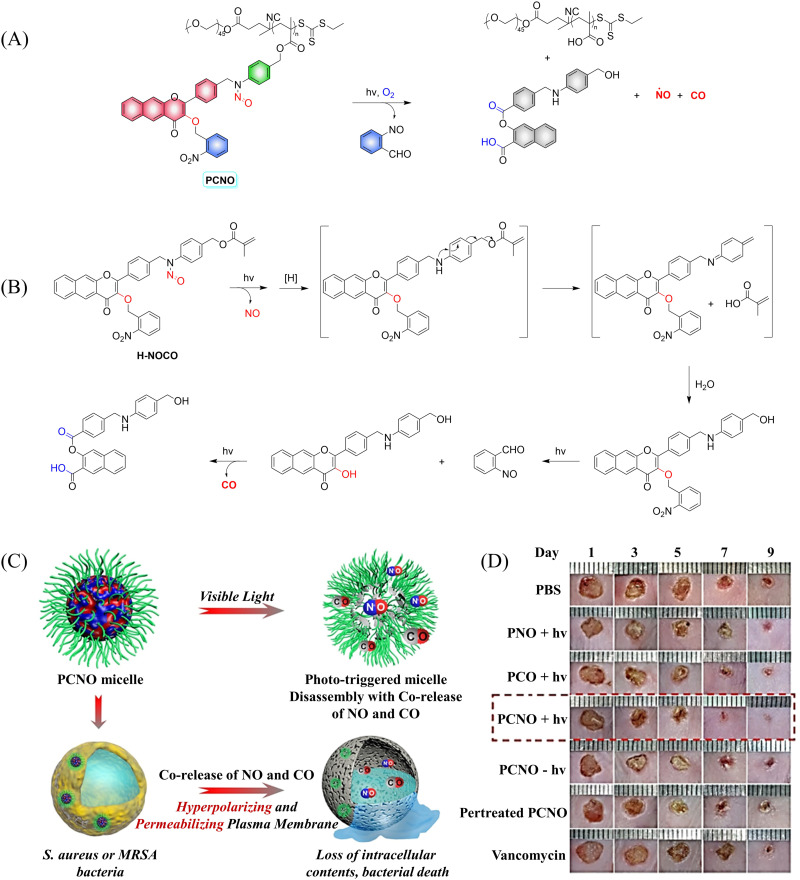
(A) The structure of PCNO, the mechanism of CO and NO release and its fluorescence changes. (B) Proposed degradation mechanism of the NO/CO-releasing H-NOCO under 410 nm light irradiation. (C) The schematic diagram of the synergistic release of NO and CO in visible-light mediated PCNO micelles can play a synergistic bactericidal role on Gram-positive bacteria through hyperpolarization and penetration of bacterial plasma membrane, leading to the loss of intracellular substances and bacterial death. (D) Evaluation of therapeutic effect on mice infected with MRSA. Parts (C) and (D) are reproduced from ref. 132 with the permission of Wiley-VCH GmbH, copyright 2021.

In addition to single-molecule donors, two-molecule donors have also attracted great interest.^131^ Recently, the Hu group developed a micelle (PCON) that releases NO and CO optically (Fig. 43(A) and (B)).^132^ The photodegradation process was investigated using H-NOCO, which released NO under 410 nm light irradiation, and the *o*-nitrobenzene group was cleaved to restore the 3-HF derivative. While the fluorescence at 603 nm of compound H-NOCO was significantly enhanced under irradiation for 50 s, the fluorescence emission decreased and CO was released with further illumination. The significant change in fluorescence intensity enabled the monitoring of the release of small molecular gases in the body. In addition, the synergistic therapy of NO and CO exhibits strong antibacterial effect towards Gram-positive bacteria, and its therapeutic ability toward MRSA infection in a mouse skin trauma model was better than vancomycin (Fig. 43(C) and (D)). This donor provides a new concept for orchestrating NO and CO signaling molecules for synergistic treatment of MRSA infections.

**Fig. 44:**
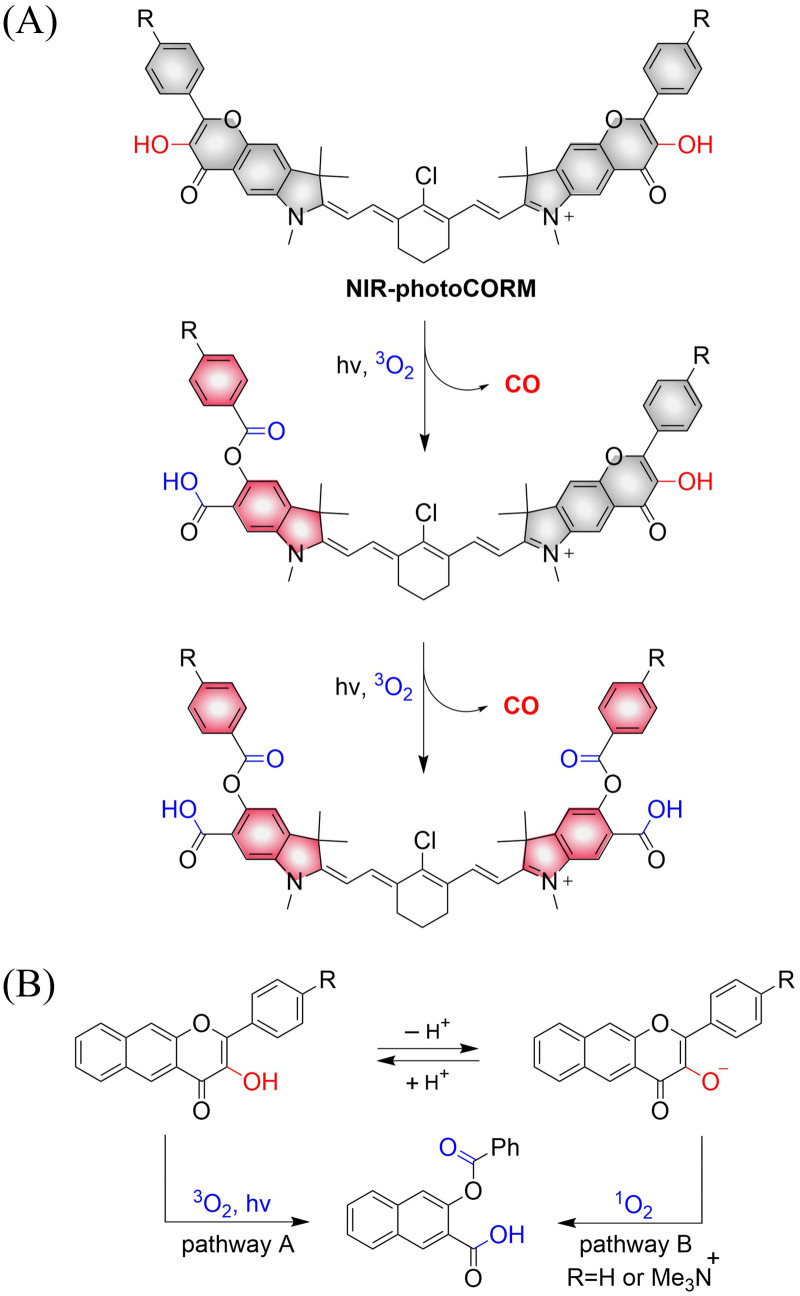
(A) The structure of NIR-photoCORM, the mechanism of CO release and its fluorescence changes. (B) Two mechanistic pathways for CO release from NIR-photoCORM.

Using similar core units, Klań extended the traditional visible-light absorption of flavonol compounds and developed a photochromic CO donor (NIR-photoCORM).^133^ NIR-photoCORM was obtained by fusing two flavonol components that release CO with NIR cyanine dyes. NIR-photoCORM upon activation with NIR light (820 nm) liberates two molecules of CO and exhibits excellent uncaging cross-sections (Fig. 44(A)). The UV/visible absorption spectra in methanol exhibits a typical strong cyanine absorption band, the emission intensity is equivalent to that of indocyanine green, enabling CO release and tracking *in vivo*. With NIR-photoCORM, the authors state that light-controlled release of CO can occur through the reaction of triplet excited state of the 3-hydroxyflavone with ground state oxygen (pathway A) or through reaction of the conjugate base with singlet oxygen (pathway B) (Fig. 44(B)).

#### 3.1.3. BODIPY carboxyl compounds

**Fig. 45:**
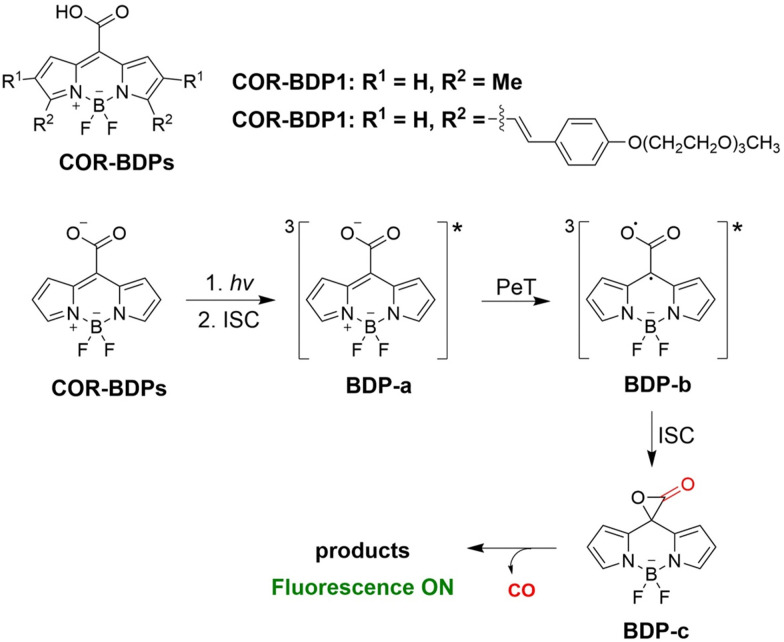
The structure of COR-BDPs, the mechanism of CO release and fluorescence changes.

From previous research most CO donors contain a flavonol moiety for releasing CO. However, relying solely on these structures to release CO is too limited. To avoid structure dependence, the Klań research group have developed transition-metal-free CO donors (COR-BDPs) based on the BODIPY chromophore.^134^ Under 300–730 nm NIR visible-light irradiation COR-BDPs can release CO, this system is suitable to penetrate biological tissues and improve the release rate of CO. It was confirmed that the carboxyl functional group was the source of the CO. The mechanism of this transformation involved light activation of COR-BDPs which results in strong fluorescence, weak intersystem crossing results in the generation of triplet BDP-a; then the carboxylic acid functional group of triplet BDP-a undergoes photoinduced electron transfer (PeT), forming an oxyallyl type triplet double radical BDP-b; subequently, intersystem crossing leads to the formation of a singlet ground-state and α-lactone BDP-c; finally, α-lactone BDP-c is cleaved to produce CO (Fig. 45). The BODIPY molecular scaffold allows for the use of simple structural modifications to alter the physical and chemical properties of COR-BDPs, providing the foundations for the development of advanced and improved CORM molecules.

### 3.2. CO release using bioorthogonal click chemistry

**Fig. 46:**
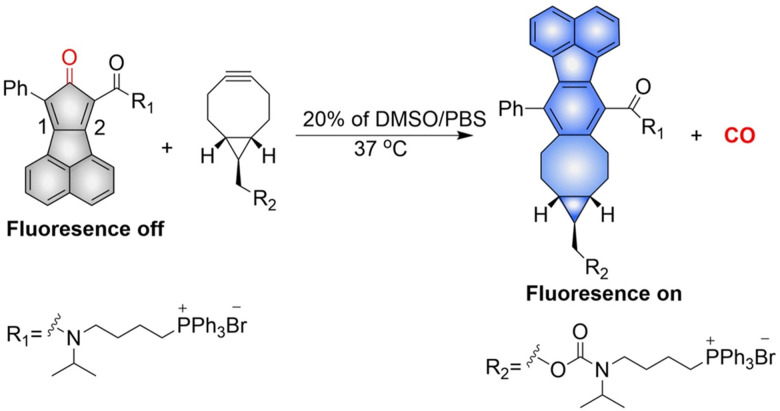
The structure of pro-drug, the mechanism of CO release and its fluorescence changes.

The Wang group have reported a method of bioorthogonal and controlled activation of prodrugs.^135^ Their system requires the enrichment of prodrugs in the mitochondria, when the concentration reaches a certain level, a click reaction is triggered which enables the localized delivery of CO (Fig. 46). Cyclopentadienones (diene in a Diels–Alder reaction) and cycloalkynes (dienophile) combined with TPP result in mitochondrial-targeting. While, incorporation of a naphthalene group to the 1, 2 position of the cyclopentadiene results in a fluorescence change after the click reaction occurs. When cyclopentadienones and alkynes are enriched in the mitochondria (mitochondrial-targeting), the Diels–Alder reaction triggers release of CO and fluorescence enhancement. This platform provides guidance on the targeted delivery of drugs to specific locations.

**Fig. 47:**
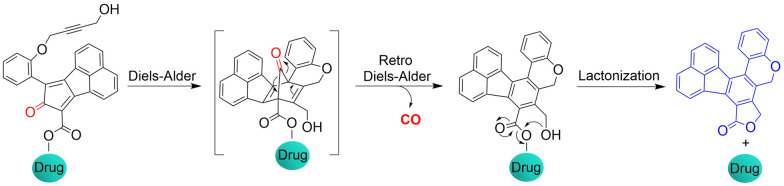
Structure of the Pro-drug, and mechanism of CO release resulting in fluorescence changes.

The Wang group also reported a strategy using intramolecular inverse electron demand Diels–Alder click reactions to release CO (Fig. 47).^136^ Significantly, the pro-drug is activated under near-physiological conditions, and the norbornene ketone intermediate formed undergoes a self-immolation reaction to release CO. The as-formed aromatic ring can effectively release carrier drugs after isomerization, accompanied by a blue fluorescence response. This cascade reaction enables synergistic treatment of CO with other drugs, such as the combined treatment of CO and antibiotic metronidazole, resulting in enhanced antibacterial efficiency.

### 3.3. ONOO-triggered CO release

**Fig. 48:**
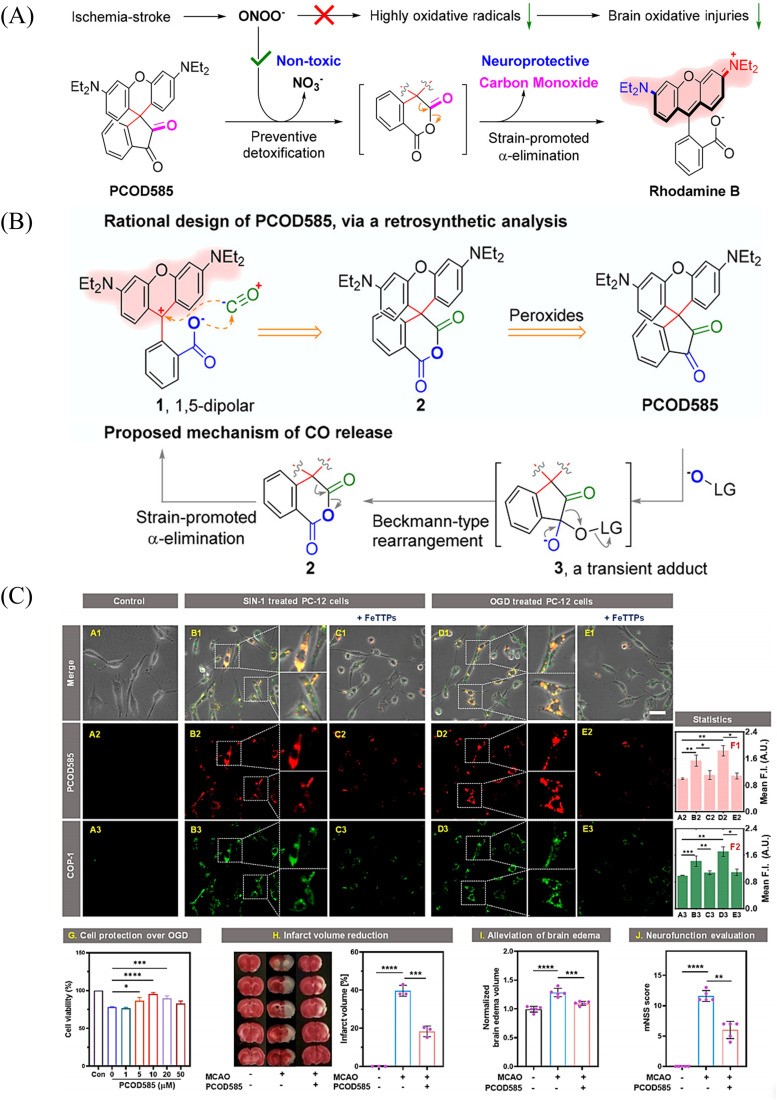
(A) and (B) Rational design of PCOD585 and its proposed mechanism of action. LG = leaving group. (C) PCOD585 was tested for *in vitro* applications in PC12 cells and EA.hy 926 cells. Reproduced from ref. 141 with the permission of the American Chemical Society, copyright 2022.

An increase in ONOO^−^ levels results in ischemia-reperfusion injury,^137,138^ while CO is neuroprotective.^139,140^ Therefore, the Yang group developed a ONOO^−^ triggered CO donor (PCOD585).^141^ Significantly, PCOD585 produces therapeutic CO whilst consuming ONOO^−^, thus realizing a multi-functional mode of action (Fig. 48(A)). PCOD585 generates ring-opened rhodamine while releasing CO (an anhydride can be produced by peroxide mediated oxidation of the 1,2-dicarbonyl, which decomposes to release CO) under ONOO^−^ activation the fluorescence turns on enabling visual-monitoring of free-radical scavenging and CO release *in vivo* (Fig. 48(B)). In addition, in the process of cerebral-reperfusion injury, PCOD585 can penetrate the blood–brain barrier, reduce the infarct volume and decreased brain edema to achieve therapeutic effect. It also exhibits a protective effect on PC-12 cells injured by OGD (Fig. 48(C)). Therefore the dual action (therapeutic CO while consuming ONOO^−^) of PCOD585 results in a potential therapeutic for the treatment of ischemic stroke.

## 4. Hydrogen sulfide

A number of H_2_S donors with fluorescence activity have been used in clinical research. Such donors combine various triggering mechanisms with self-reporting fluorescence markers to visualize and quantify H_2_S release. These donors can be divided into two categories in terms of the release mode: directly and indirectly produced H_2_S. The latter adopts a completely different strategy, which indirectly produces H_2_S from carbonyl sulfide (COS).

### 4.1. Direct production of H_2_S donor

H_2_S is inherently toxic, and historically, research has primarily focused on its toxicity, with limited attention given to its physiological and pathological functions.^142^ It was not until 1996, with Abe and Kimura's discovery that mammals produce H_2_S gas endogenously and use it as a neuromodulator, did the understanding of the roles of H_2_S begin to shift.^143^ Further research has shown that H_2_S is an important gaseous signaling molecule, following NO and CO. H_2_S can regulate vascular remodeling,^144^ mediate neurotransmission,^145^ lower blood pressure,^146^ and regulate insulin release.^147^ In myocardial ischemia-reperfusion injury, H_2_S exhibits a strong cardioprotective effect.^148^ Additionally, H_2_S plays a crucial role in the treatment of stroke, various cancers, and liver diseases.^149,150^

#### 4.1.1. Photo controlled H_2_S release

**Fig. 49:**
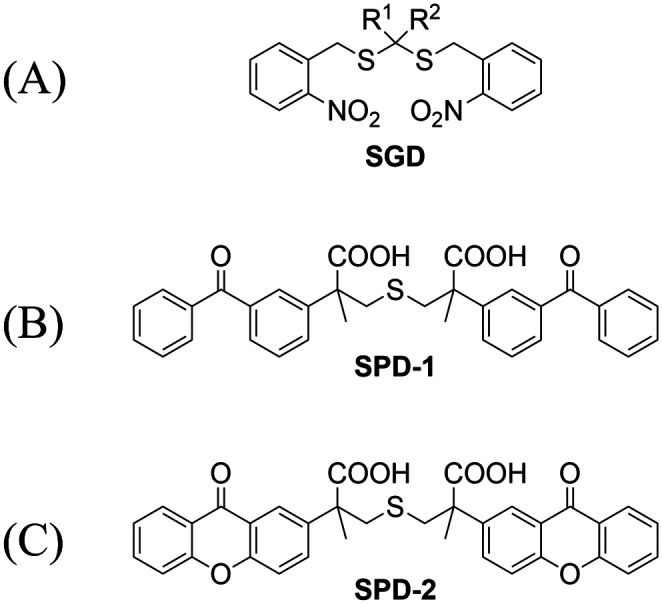
The structures of SGD (A), SPD-1 (B), SPD-2 (C).

Significant interest surrounds research into the release of small molecules by photolysis, since a high degree of control over the location, time, and dose can be provided.^151–156^ At present, many platforms based on the photocontrol of small molecules to produce H_2_S have been developed, however most of them are activated by UV light. The Xian group have reported the photolysis of SGD which is based on a *gem*-dithiol structure to release H_2_S (Fig. 49(A)).^157^ However, it is difficult to control the rate of H_2_S release from such donors, since the formation of H_2_S depends on the hydrolysis of *gem*-dithiols in an aqueous medium.^158^ The group of Nakagawa have reported a photocontrollable H_2_S donor based on a ketoprofenate photocage (SPD-1) (Fig. 49(B)).^159^ Although the donor has many excellent properties compared with *gem*-dithiol donors (good water solubility, generation of benign byproducts and higher release rate than *o*-nitrobenzyl). It can not be used for living cell applications, since many microscopes use UVA (330–380 nm), which is not a suitable light source for activating SPD-1. In order to solve this problem, the same research group developed a new light induced xanthones H_2_S donor suitable for living cell applications (SPD-2) (Fig. 49(C)).^160^ Compared with the previous photo activation conditions for *gem*-dithiols and ketoprofenate donors, this method provides excellent control by 325–385 nm irradiation.

**Fig. 50:**
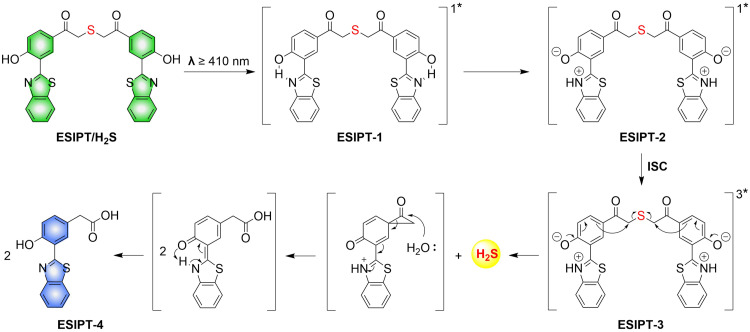
The structure of ESIPT/H_2_S, and the mechanism of H_2_S release and fluorescence changes.

To solve the problems associated with UV light activation, a new H_2_S donor was designed (ESIPT/H_2_S) by the Singh group.^161^ Under ≥410 nm light irradiation, ESIPT/H_2_S is excited to its singlet state ESIPT-1, which triggers a fast proton transfer reaction from the hydroxyl to benzothiazole generating intermediate ESIPT-2. Then, intermediate ESIPT-2 undergoes effective intersystem crossing (ISC) and enters its triplet excited state ESIPT-3. Intermediate ESIPT-3 then rearranges into carboxylic acid derivative ESIPT-4 by a photo-Favorskii reaction, with the release of H_2_S (Fig. 50). Compared with traditional H_2_S donors, this donor exhibits good biocompatibility, cellular internalisation, and less cytotoxicity, which lays the foundation for effective use in biomedical applications.

**Fig. 51:**
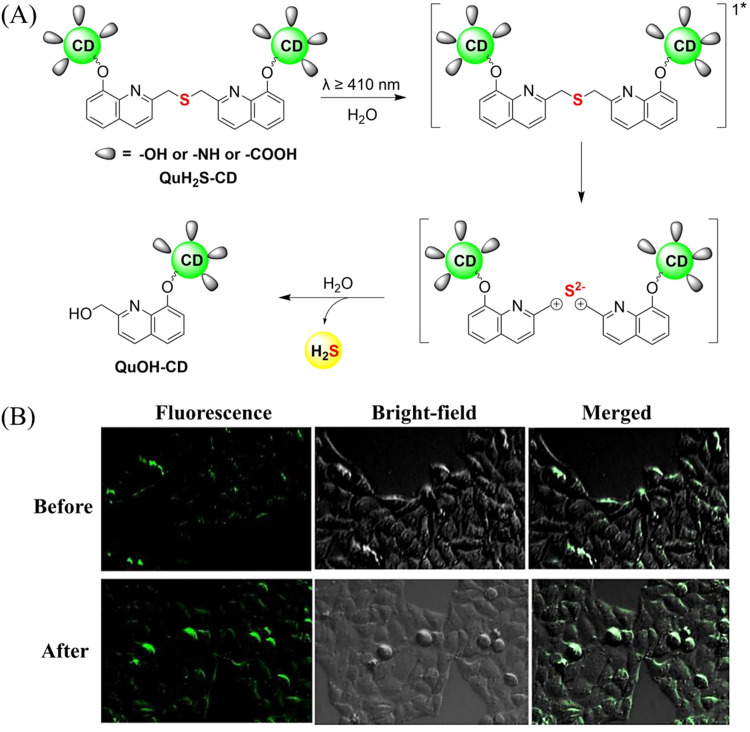
(A) Proposed Photodissociation mechanism of QuH_2_S-CDs. (B) The ability of cellular uptake of QuH_2_S-CD was observed by confocal microscope imaging. Release of H_2_S from QuH_2_S-CDs was monitored before and after irradiation for 50 min with light (*λ* ≥ 410 nm). Reproduced from ref. 162 with the permission of the Royal Society of Chemistry, copyright 2020.

The same group also developed a visible light responsive H_2_S nano-carrier (QuH_2_S-CD). The carrier is composed of fluorescent carbon dots and quinoline, and exhibits good biocompatibility, large fluorescence changes and efficient release of H_2_S (Fig. 51(A) and (B)).^162^ Under visible light irradiation, quinoline is excited into a singlet state. Subsequent carbon–sulfur bond breakage, results in formation of quinoline carbon cations and sulfide anions. Finally, H_2_S is generated by quenching with water. This QuH_2_S-CD system illustrates the importance of nano-carriers for biomedical applications.

**Fig. 52:**
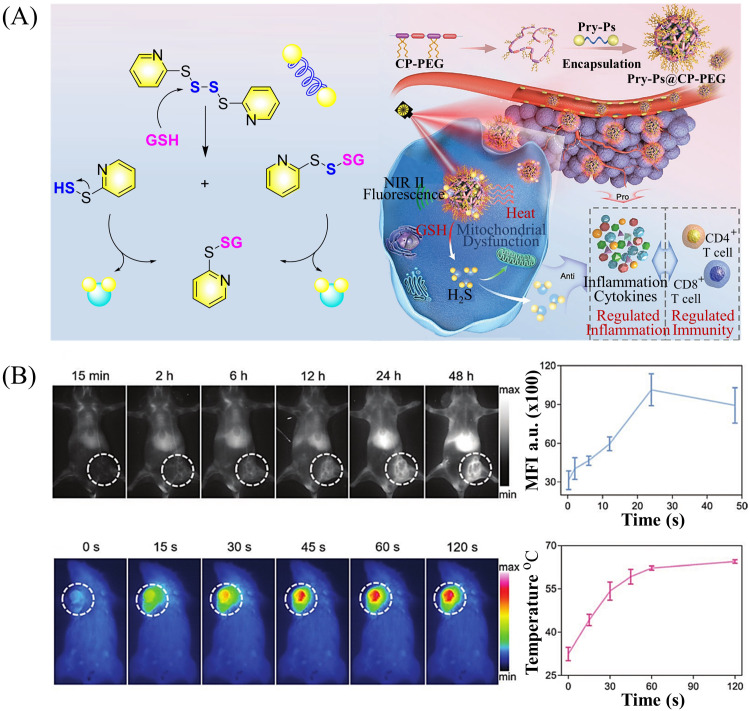
(A) The structure of Pry-Ps, the mechanism of H_2_S release. Schematic illustration of the NIR-II fluorescence tracked Pry-Ps@CP-PEG. (B) Pry-Ps@CP-PEG fluorescence and photothermal imaging in mice. Parts (A) and (B) are reproduced from ref. 168 with the permission of Wiley-VCH GmbH, copyright 2021.

Multiple-action combined therapy is a new concept for cancer treatment.^163–167^ The Dai group have developed a nano-regulator for the synergistic treatment by GT and PTT (Pry-Ps@CP-PEG).^168^ In this nanosystem, a hydrophobic polysulfide H_2_S donor (Pry-Ps) was encapsulated using amphiphilic conjugated polymers (CP-PEG), resulting in Pry-Ps@CP-PEG with good biocompatibility. Under 808 nm laser irradiation, tumor-rich GSH can trigger Pry-Ps to release H_2_S *via* the cleavage of an S–S bond, resulting in mitochondrial dysfunction and a strong anti-inflammatory effect (Fig. 52(A)). The *in vivo* NIR-II fluorescence and efficient photothermal conversion of Pry-Ps@CP-PEG was also determined (Fig. 52(B)). In addition, H_2_S-regulated PTT is conducive to adaptive immune response, and has shown excellent efficacy in the treatment of skin trauma.

#### 4.1.2. Thiol triggered H_2_S release

**Fig. 53:**
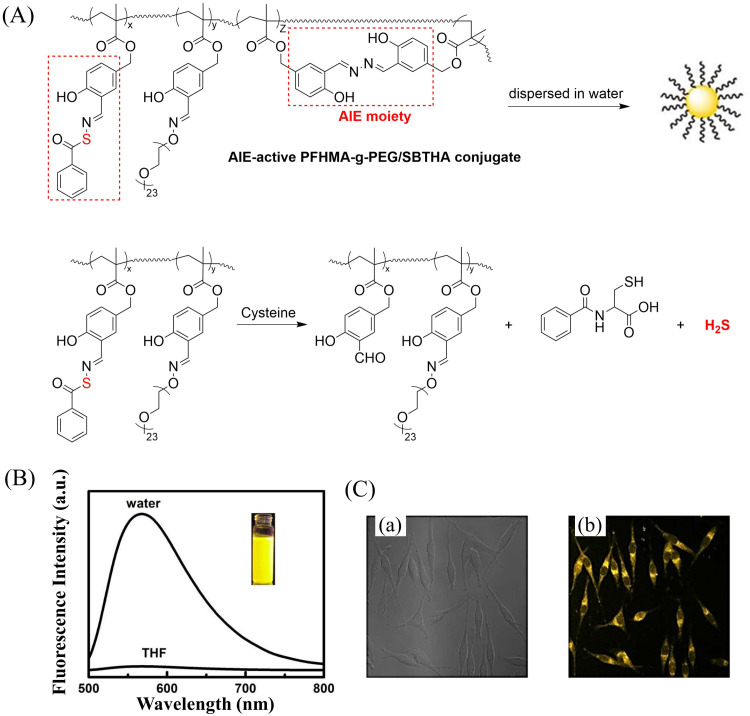
(A) The structure of AIE-active PFHMA-*g*-PEG/SBTHA, the mechanism of H_2_S release. (B) Fluorescence spectrum of AIE-active PFHMA-*g*-PEG/SBTHA in water or THF (*λ*_ex_ = 390 nm, *λ*_em_ = 570 nm). Inset: Fluorescence image in water under 365 nm UV light. (C) CLSM images of L929 cells after incubation with 50 μg mL^−1^ of AIE-active PFHMA-*g*-PEG/SBTHA conjugate (*i.e.* salicylaldazine moieties). (a) Bright field, (b) excited with a 390 nm laser. Reproduced from ref. 172 with the permission of the Royal Society of Chemistry, copyright 2018.

Bio-thiol triggered H_2_S donors do not require additional triggering conditions, resulting in good compatibility with biological systems.^169–171^ The Lu group have developed a bio-thiol triggered AIE-active polymeric H_2_S donor.^172^ Firstly, a polymer PFHMA was prepared by the reversible addition–fragmentation chain transfer polymerisation of 3-formyl-4-hydroxybenzyl methacrylate (FHMA). Then, the PFHMA was conjugated with PEG_23_-ONH_2_, hydrazine and *s*-benzoyl thiohydroxylamine (SBTHA) respectively to obtain the PFHMA-*g*-PEG/SBTHA conjugate with AIE activity. The conjugate contains *S*-aroylthiooxime (SATO) units, which can slowly release H_2_S in response to Cys or GSH. Significantly. the H_2_S release rate can be controlled by changing the substituents of the SATO (Fig. 53(A)). In addition, the polymer exhibits good water solubility (inherent to the PEG), and AIE enables the release of H_2_S in living cells to be monitored by fluorescence imaging (Fig. 53(B) and (C)).

**Fig. 54:**
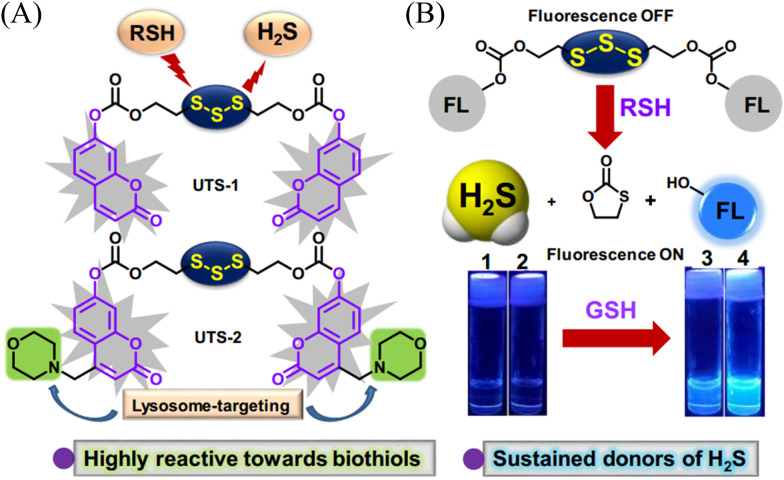
(A) The structure of UTS-1 and UTS-2. (B) The mechanism of H_2_S release and its fluorescence changes. Images of UTS-1 (20 μM, vial 1, 2) and UTS-1 (20 μM) with GSH (100 μM, vial 3; 200 μM, vial 4) in DMSO after 15 min. Reproduced from ref. 173 with the permission of the Royal Society of Chemistry, copyright 2020.

Organic trisulfide (self-immolative fluorogenic donors) triggered by bio-thiols can be used to effectively monitor intracellular H_2_S and lysosomal transport of H_2_S, laying the foundation for the development of H_2_S mediated disease treatment. The Bhabak group have developed a bio-thiol triggered H_2_S donor (UTS-1 and UTS-2), the core group of the donor comes from organic trisulfide.^173^ When activated by bio-thiols, UTS-1 and UTS-2 release H_2_S in a controlled manner (Fig. 54(A)), and generate a significant fluorescence response suitable for monitoring intracellular H_2_S release in real-time (Fig. 54(B)). In addition, both the donors have good compatibility with water and cell media and UTS-2 exhibits organelle specificity.

**Fig. 55:**
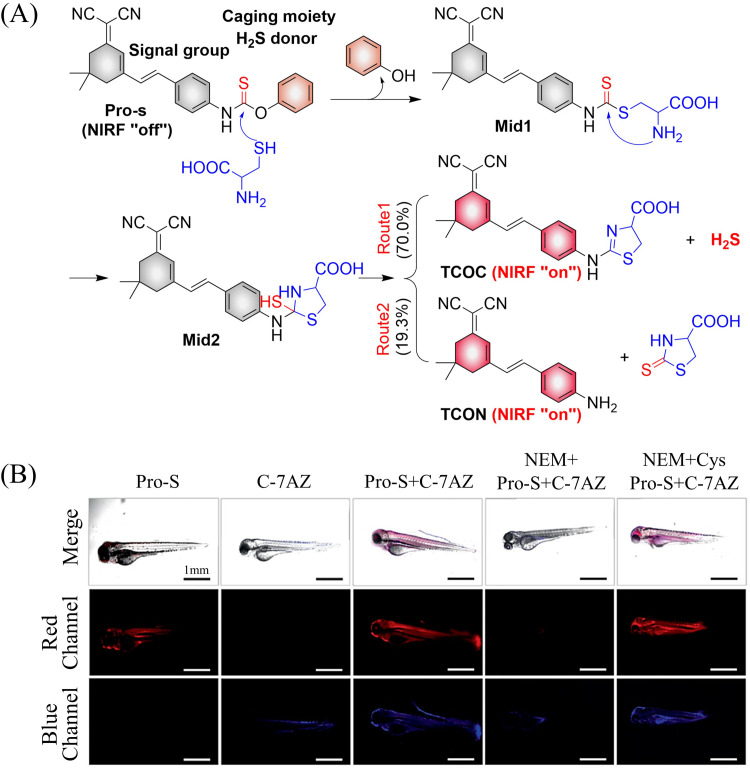
(A) The structure of Pro-s, the mechanism of H_2_S release and its fluorescence changes. (B) Pro-s releases H_2_S confocal fluorescence imaging in zebrafish. Reproduced from ref. 175 with the permission of the American Chemical Society, copyright 2021.

It is well known that H_2_S has a protective effect for cells due to its anti-inflammatory and anti-apoptotic activity.^174^ Therefore, the development and research toward H_2_S donors is crucial to understand the potential mechanism of inflammation and optimize treatment interventions. The Zhang group developed a Cys triggered NIR fluorescent H_2_S donor (Pro-s).^175^ Because Pro-s contains a strong electron withdrawing carbon-sulfur double bond, the electron donating ability of the amino group decreases, resulting in low fluorescence output. The sulfhydryl of Cys selectively attacks the Pro-s carbon–sulfur double bond to produce intermediate Mid1. Then intramolecular cyclization occurs to form intermediate Mid 2. Intermediate Mid2 further cyclizes to produce TCOC and H_2_S, while TCON and *N*-acetylcysteine are also produced (Fig. 55(A)). Pro-s exhibits strong NIR fluorescence enhancement (70-fold), controlled H_2_S release (30 minutes), high H_2_S release efficiency (62%) and good live cell compatibility. Enabling the visual monitoring of H_2_S release in cells and zebrafish (Fig. 55(B)), in addition the anti-inflammatory effect of Pro-s has been confirmed in macrophages (RAW 264.7).

**Fig. 56:**
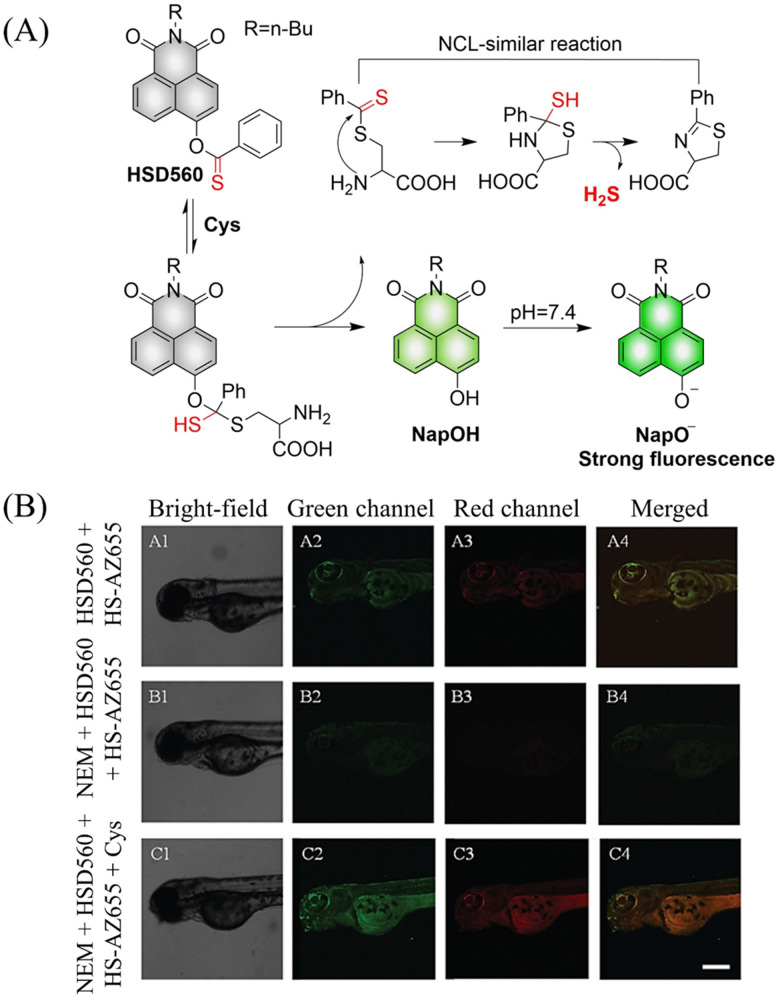
(A) The structure of HSD560, the mechanism of H_2_S release and its fluorescence changes. (B) HSD560 released H_2_S confocal fluorescence imaging in zebrafish. Reproduced from ref. 176 with the permission of the Royal Society of Chemistry, copyright 2021.

In 2021, the Zhang group developed a Cys triggered H_2_S donor (HSD560).^176^ The fluorescence of HSD560 is quenched by the thionoester group. When the thionoester is exposed to an enriched Cys environment, HSD560 will undergo a non-enzymatic native chemical ligation (NCL) process to release H_2_S. In biological systems, the generated NapOH is further deprotonated to generate NapO^−^, thereby restoring the ICT effect (Fig. 56(A)). The generated green fluorescence enables the visual monitoring of H_2_S release in zebrafish (Fig. 56(B)). HSD560 could reduce the levels of NO and Prostaglandin E2, achieving anti-inflammatory effect. Compared to COS/H_2_S donors, the H_2_S release of HSD560 is non-enzymatic. Therefore, this donor may be a promising molecular tool for the delivery and physiological research of H_2_S in complex biological environments.

### 4.2. Indirectly produced H_2_S

**Fig. 57:**
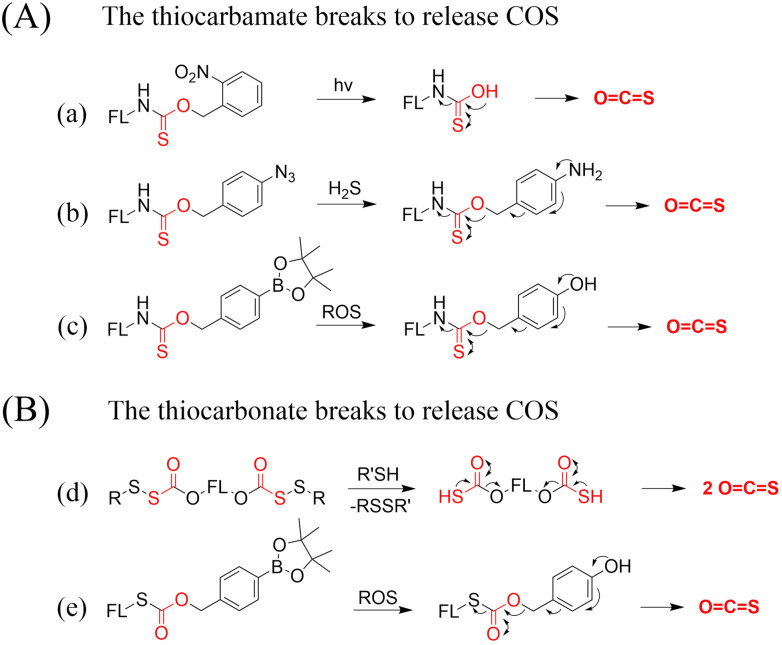
Under different triggering conditions, the thiocarbamate (A)/thiocarbonate (B) breaks to release COS.

We now cover previously reported indirect H_2_S donors. Where, following a series of reactions, these donors release hydrolyzable COS, and then COS is rapidly hydrolyzed to H_2_S by the ubiquitous carbonic anhydrase (CA). There are two possible ways for such donors to release COS: through thiocarbamate/thiocarbonate cleavage to generate COS. The specific mechanisms are shown in Fig. 57. In addition, the donor can release COS and a fluorophore, thus providing an optical response. The strategy of using reactive intermediates to produce COS for subsequent H_2_S release is now a widely used method for constructing H_2_S donors.^177–180^

We have elucidated the biological significance of H_2_S by employing self-immolative thiocarbamate as COS donors with fluorescence activity. For example, *o*-nitrobenzyl-substituted thiocarbamate undergoes photoinduced nitro group transformation to yield 2-nitrobenzaldehyde and thiocarbamate intermediates, the thiocarbamate intermediates then decompose to generate COS and the corresponding fluorescent moieties (Fig. 57(a)).^181^ An azido benzene-substituted thiocarbamate, under H_2_S attack, undergoes 1,6-elimination to produce thiocarbamate intermediates that further decompose to release COS (azido compound serving as the H_2_S recognition site) (Fig. 57(b)).^182^ Analogously, an arylboronic acid ester-substituted thiocarbamates, under ROS conditions, also undergoes 1,6-elimination to ultimately yield COS (Fig. 57(c)).^183^ Examples of systems where a thiocarbonate breaks to release COS, include the example where cellular thiols (Cys and GSH) activate sulfenyl thiocarbonates through thiol-mediated disulfide reduction to release COS, which is quickly converted to H_2_S by CA (Fig. 57(d)).^184^ In addition, arylboronic acid ester-substituted thiocarbonate, under ROS treatment, can undergoes 1,6-elimination to yield COS (Fig. 57(e)).^185^ The highly modular approach outlined above for thiocarbamate/thiocarbonate cleavage offers a COS-based H_2_S donor library, which can be activated through specific stimuli to achieve H_2_S release that can be visualized.

#### 4.2.1. Photo controlled H_2_S release

**Fig. 58:**
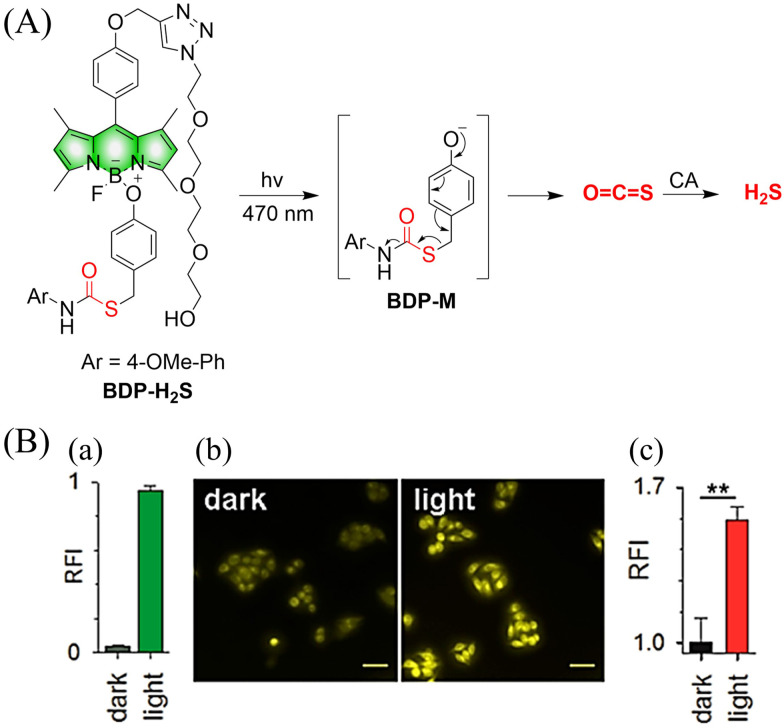
(A) Proposed decomposition mechanism of BDP-H_2_S. Under 470 nm light irradiation, the B–O bond of BDP-H_2_S is broken to produce intermediate BDP-M. Then intermediate BDP-M undergoes 1,6-elimination to generate COS, which is rapidly hydrolyzed to H_2_S in the presence of CA. (B) Fluorescence intensity comparison of BDP-H_2_S before and after light triggering (*λ*_ex_ = 470 nm, *λ*_em_ = 540 nm). Reproduced from ref. 187 with the permission of the American Chemical Society, copyright 2017.

Photo-controlled drug release has completely changed the structure of biomedical research, since it enables interventions on diseased cells in a non-invasive manner.^186^ Chakrapani and his group developed a visible light triggered H_2_S donor (BDP-H_2_S).^187^ Under 470 nm visible light irradiation, BDP-H_2_S releases COS through thiol bond cleavage of the thiocarbamate, and then COS is rapidly hydrolyzed to H_2_S in the presence of CA (Fig. 58(A)). The fluorescence signal of HeLa cells treated with BDP-H_2_S was significantly enhanced (Fig. 58(B)).

**Fig. 59:**
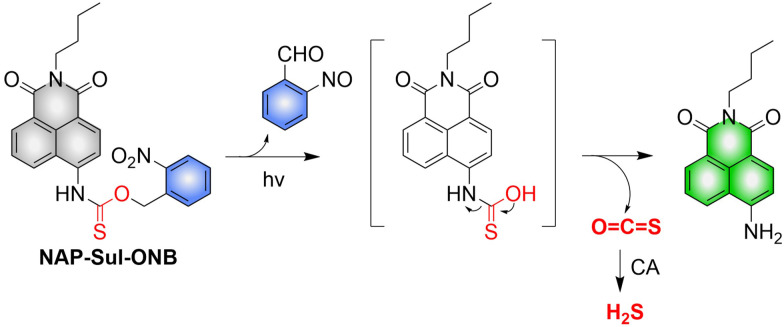
Proposed photodissociation mechanism of Nap-Sul-ONB. Under 365 nm light activation, the photolysis group (*o*-nitrobenzyl) undergoes a self-elimination reaction to release COS, and then COS is rapidly hydrolyzed to H_2_S in the presence of CA.

The introduction of photo-active protective group (nitrobenzyl) into a compounds structure can enable the precise activation of functional molecules by light. The Gou group developed an light-controlled H_2_S donor (NAP-Sul-ONB) derived from naphthalimide (Fig. 59).^181^ NAP-Sul-ONB was generated by connecting an amine substituted 1,8-naphthalimide and *o*-nitrobenzyl using a thiocarbamate. The thioamide of the amine on the 1,8-naphthalimide alters the excitation/emission wavelengths. However, once the amine is exposed, the photophysical properties are restored. In addition, under stimulation with 365 nm light, Nap-Sul-ONB can not only release H_2_S, but also reduces the ROS levels of phorbol-12-myristate-13-acetate treated cells and improves the survival rate of the cells.

#### 4.2.2. Thiols trigger H_2_S release

**Fig. 60:**
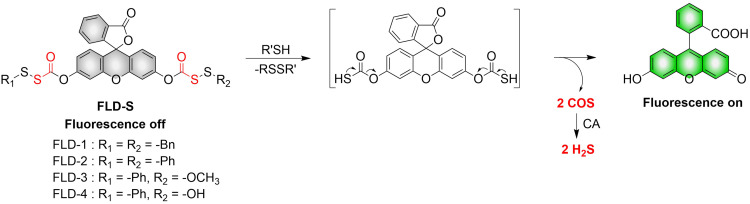
Proposed decomposition mechanism of FLD-S. Under thiol triggered, FLD-S undergoes a cascade reaction to release COS, and then COS is rapidly hydrolyzed to H_2_S in the presence of CA.

To improve the amount of the released H_2_S, the Pluth group reported the synthesis of sulfenyl thiocarbonates as novel fluorescent COS/H_2_S donors (FLD-S) (Fig. 60).^184^ With this design strategy, the amount of H_2_S released can be controlled by the choice of fluorophore. This means that one molecule of H_2_S is released in a single substitution and two molecules of hydrogen sulfide are released by a double substitution. Thus, H_2_S can be released on-demand. Triggered by GSH/Cys, FLD-S releases COS and fluorescein through thiol bond cleavage of sulfenyl thiocarbonates. Subsequently, COS is quickly hydrolyzed to H_2_S under the catalysis of CA, realizing the real-time monitoring of H_2_S release in complex environments. In addition, the anti-inflammatory activity of H_2_S was also indicated since FLD-1 exhibited a dose-dependent inhibition of LPS-induced NO formation.

**Fig. 61:**
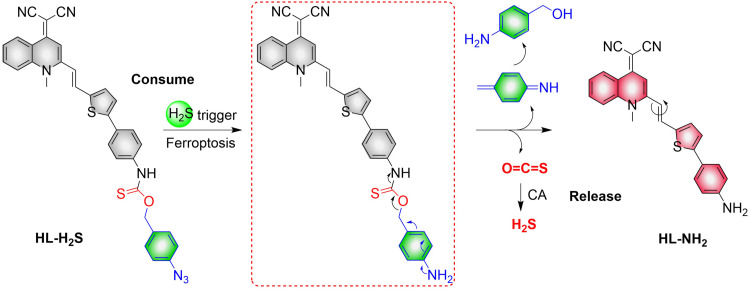
Proposed decomposition mechanism of HL-H_2_S. Triggered by H_2_S, the azide group of HL-H_2_S is reduced, thus undergoes self-immolation to generate COS, and then COS is rapidly hydrolyzed to H_2_S in the presence of CA.

According to previous reports, cystathionine-β-synthase (CBS) and cystathionine-γ-lyase (CSE) can produce endogenous H_2_S through Cys desulfurization.^188–190^ Because the ferroptosis protein inhibits CBS/CSE, a reduction of H_2_S occurs and the redox balance in cells is broken and the process of iron cell death is aggravated. Therefore, H_2_S, as a representative reducing substance, can be used to monitor the ferroptosis process.^191,192^ As such, in 2022, a multi-functional NIR-H_2_S fluorescent probe (HL-H_2_S) was developed by the Qiang group (Fig. 61).^182^ In the imaging process, HL-H_2_S consumes H_2_S and undergoes 1,6-elimination to release COS, then COS is rapidly hydrolyzed to H_2_S under the catalysis of CA (H_2_S has strong reduction properties, which can reduce the electron-withdrawing azido group to an electron-donating amino group). In addition, the malonitrile structure in HL-H_2_S is responsive to viscosity. This strategy can maintain the redox state of cells without aggravating the process of ferroptosis. The dual action mechanism realizes the visual monitoring in high-fidelity of the ferroptosis process.

#### 4.2.3. Reactive oxygen species triggered H_2_S release

**Fig. 62:**
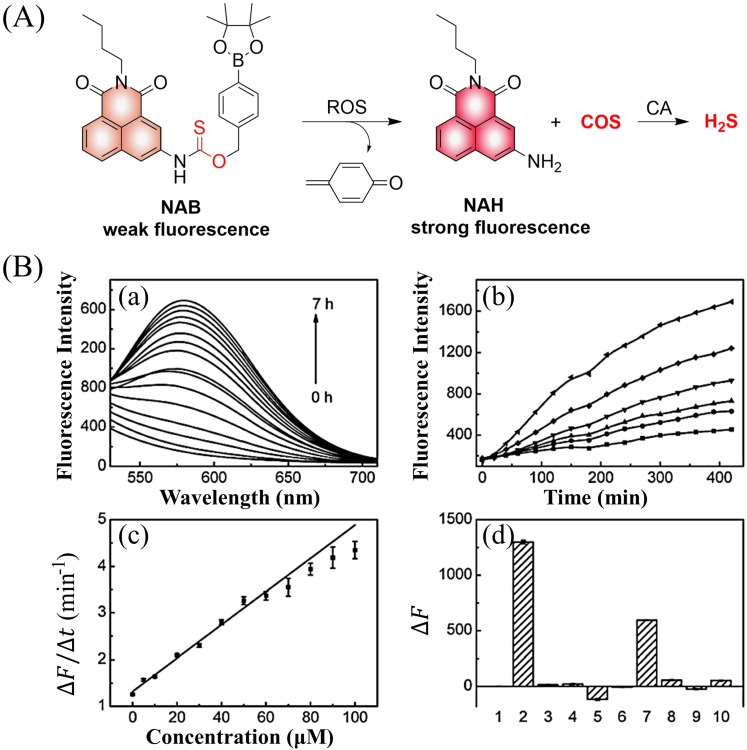
(A) Proposed decomposition mechanism of NAB. (B) (a) Fluorescence spectra of NAB (10 μM) with H_2_O_2_ (100 μM) at various times in phosphate buffer (20 mM, pH 7.4). (b) Fluorescence kinetic curves of NAB (10 μM) with different H_2_O_2_ concentrations. (c) Linear relationship between fluorescence intensity and H_2_O_2_ concentrations. (d) Fluorescence changes of NAB (10 μM) in the presence of different ROS species. (1) NAB only; (2) H_2_O_2_ (100 μM); (3) NO (100 μM); (4) ClO^−^ (100 μM); (5) ˙OH (100 μM); (6) ^1^O_2_ (100 μM); (7) O_2_^−^ (100 μM); (8) TBHP (100 μM); (9) TBO˙ (100 μM); (10) ONOO^−^ (10 μM). (*λ*_ex_ = 405 nm, *λ*_em_ = 577 nm). Reproduced from ref. 183 with the permission of the Royal Society of Chemistry, copyright 2019.

It is well known that excessive ROS in the body results in oxidative stress, which is the basis of disease development. Therefore, it is important to develop a donor that can not only remove excess ROS in the body but is easy to prepare and provides spatiotemporal control and a high release rate. For example, NAB, HSD545, HSD-R, HSD-B and Zl47, all these donors contain a phenylboronic acid pinacol ester as the ROS recognition site, scavenging ROS and producing H_2_S simultaneously. The group of Ma combined arylboronic acid ester with a fluorophore 3-amino-*N*-butyl-1,8-naphthalimide using a thiocarbamate, and developed the first ROS triggered fluorescent H_2_S donor (NAB) with self-reporting ability (Fig. 62(A)).^183^ When activated by ROS, NAB releases COS through a self-immolation reaction. Subsequently, COS is rapidly hydrolyzed to H_2_S under catalysis by CA, and the fluorescence is turned on to enable the real-time monitoring of H_2_S release and transportation in biological systems (Fig. 62(B)). These aspects of NAB resulted in a protective effect toward RAW 264.7 cells during oxidative stress.

**Fig. 63:**
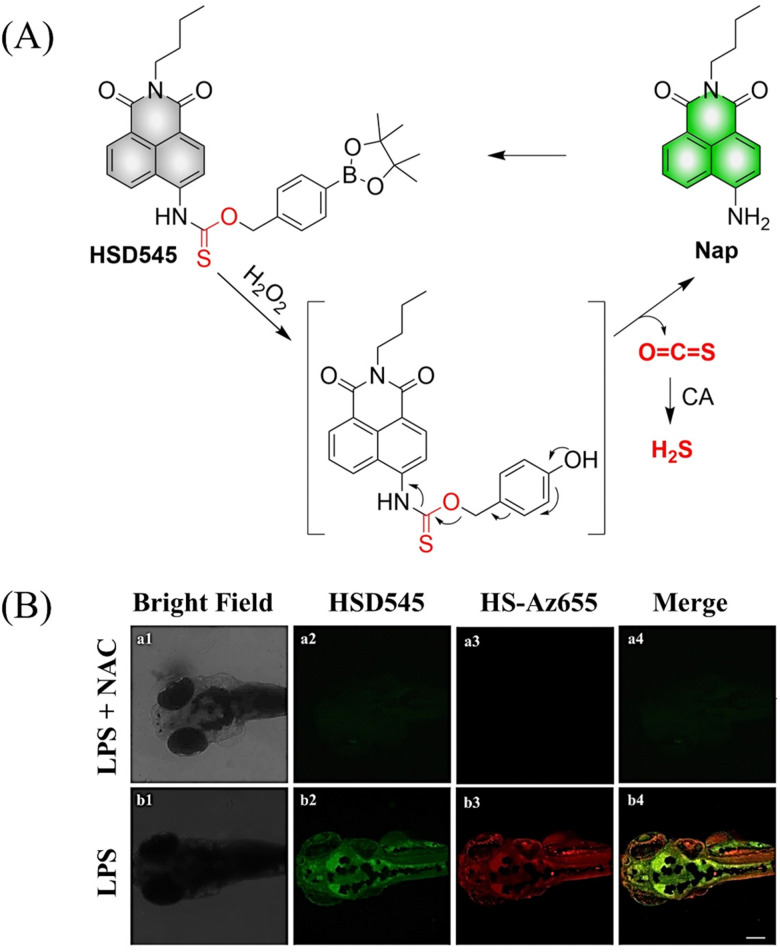
(A) Proposed decomposition mechanism of HSD545 releasing H_2_S. (B) Fluorescence imaging of H_2_S released by HSD545 in zebrafish. Reproduced from ref. 193 with the permission of Elsevier B.V., copyright 2021.

The Zhang group developed a ROS triggered H_2_S donor (HSD545) (Fig. 63(A)).^193^ Triggered by H_2_O_2_, HSD545 undergoes a 1,6-elimination reaction to release COS, and then COS is rapidly hydrolyzed to H_2_S in the presence of CA, and a bright fluorescence response is also generated, thus realizing real-time monitoring of H_2_S release *in vivo* and *in vitro* (Fig. 63(B)). This donor exhibits low cytotoxicity and strong cytoprotection against ROS induced oxidative stress. Compared with previous NAB donors, changing the position of the trigger does not significantly affect the system.

**Fig. 64:**
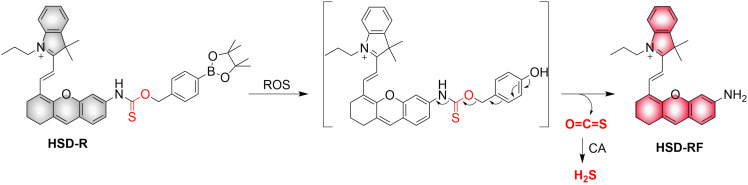
Proposed decomposition mechanism of HSD-R.

While significant advances have been made in the targeted release of H_2_S, a detailed understanding of the intracellular redox mechanisms under certain physiological and pathological conditions remains elusive, especially a detailed exploration of H_2_S levels in the pathological models of myocardial infarction. The Zhang group reported a H_2_S donor (HSD-R) that exhibits a fluorescence response under ROS stimulation (Fig. 64).^25^ HSD-R can target the mitochondria and respond to overexpressed ROS. After self-immolation the COS moiety is released, and red fluorescence is generated. During treatment, HSD-R significantly promotes the reconstruction of cardiac structure and function in a rat model of myocardial infarction and achieved cardiac protection by inhibiting proapoptotic genes (Bid, Apaf-1 and p53). As such, this ROS-responsive, self-immolative, and fluorescent H_2_S donor can serve as a new theranostic agent for myocardial infarction and other ischemic diseases.

**Fig. 65:**
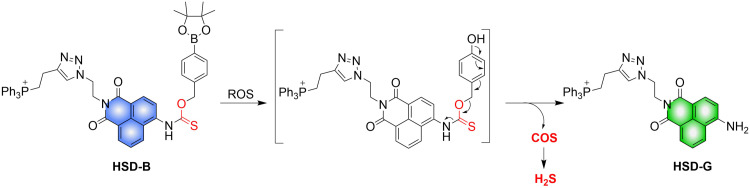
Proposed decomposition mechanism of HSD-B releasing H_2_S.

Similarly, the He group reported a new ratiometric fluorescent H_2_S donor (HSD-B) (Fig. 65).^194^ In the presence of ROS, the pinacol phenylboronate reacts and releases COS. Subsequently, COS is rapidly hydrolyzed to H_2_S under the catalysis of CA. At the same time, thiocarbamate substituted naphthalimide is converted to amine substituted naphthalimide HSD-G, and the fluorescence changes from blue to green. The advantages of this system are: (i) scavenging ROS and producing H_2_S simultaneously, realizing a dual mechanism (scavenging ROS and producing H_2_S simultaneously) of action in cells; (ii) obvious fluorescence changes, providing a simple monitoring method for the visualization and quantification of H_2_S release; (iii) by targeting the mitochondria, delivery of H_2_S is localized. In addition, the system exhibits a protective effect for myocardial ischemia reperfusion injury in a cell model.

**Fig. 66:**
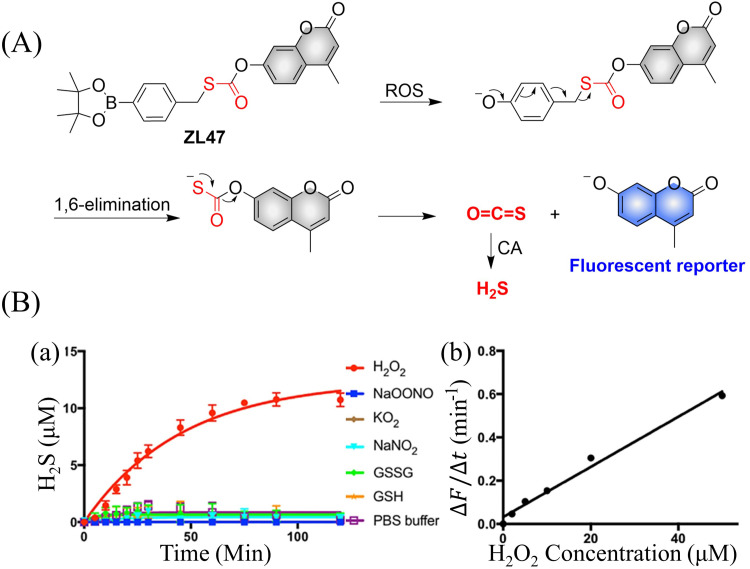
(A) Proposed decomposition mechanism of ZL47. (B) (a) Time-dependent H_2_S release from ZL47. (b) Linear relationship between fluorescence intensity and H_2_O_2_ (*λ*_ex_ = 365 nm, *λ*_em_ = 448 nm). Reproduced from ref. 185 with the permission of Elsevier Ltd, copyright 2021.

The Lukesh III group linked an arylboronic acid ester to 7-hydroxy-4-methylcoumarin using a thiocarbonate alkyl ester bond to prepare a H_2_S donor (Zl47) (Fig. 66(A)).^185^ Under the stimulation of ROS, the mechanism of H_2_S release by Zl47 is consistent with that of similar donors. Therefore, Zl47 was suitable for cell imaging and alleviating oxidative stress in living cells and exhibits potential as a therapeutic agent for diseases related to ROS overexpression (Fig. 66(B)).

#### 4.2.4. H_2_S release using bioorthogonal click chemistry

**Fig. 67:**
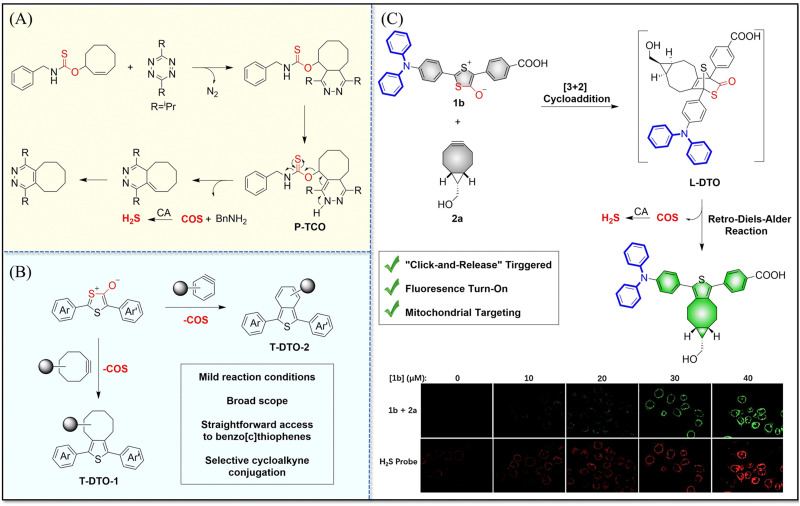
(A) and (B) P-TCO,T-DTOs structure, COS release mechanism and fluorescence changes. (C) L-DTO structure, COS release mechanism and fluorescence changes. Reproduced from ref. 201 with the permission of Wiley-VCH GmbH, copyright 2021.

Biorthogonal “click and release” has many potential applications in a biological environment.^195–198^ The Pluth group developed a COS/H_2_S donor (P-TCO) activated by a bio-orthogonal reaction (Fig. 67(A)).^199^ The reaction utilizes the inverse electron demand Diels–Alder click reaction between thiocarbamate functionalized *trans*-cyclooctene (TCO) and tetrazine to generate COS which is rapidly hydrolyzed by CA to release H_2_S. The system represents a nice biorthogonal COS/H_2_S donation strategy. The Taran group used the cycloaddition reaction between 1,3-dithiolium-4-olates (DTOs) and cyclooctyne or phenylacetylene derivatives (T-DTOs), thus providing a versatile platform to access benzo[*c*]thiophenes and dithiophene-diphenylene structures and COS in an unprecedented divergent fashion (Fig. 67(B)).^200^ While the Liang group developed a DTO compound, which can react with a strained alkyne *via* the 1,3-dipolar cycloaddition and the retro-Diels–Alder reaction to generate carbonyl sulfide (COS) as the precursor of H_2_S, and a thiophene derivative with turn-on fluorescence (L-DTO) (Fig. 67(C)).^201^ Moreover, the introduction of diphenylamino enables mitochondrial targeting and fluorescence tracking of living cells. In addition, the donor alleviates the loss of mitochondrial membrane potential of H9C2 cells under oxidative stress, providing a novel strategy for GT.

#### 4.2.5. Carbonyl sulfide (COS)/hydrogen sulfide (H_2_S) release

**Fig. 68:**
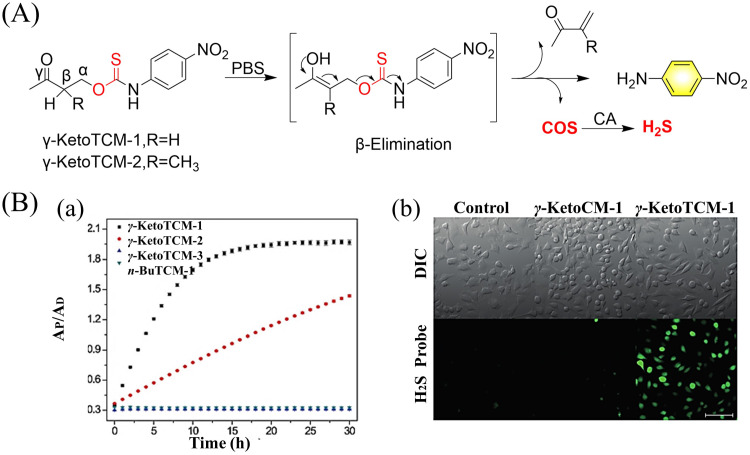
(A) γ-KetoTCM-1-2 structure, H_2_S release mechanism and fluorescence changes. (B) (a) Formation of *p*-nitroaniline (PNA) after compound activation. (b) H_2_S Delivery from γ-KetoTCM-1 in HeLa cells. HeLa cells were treated with a cell-trappable H_2_S fluorescent probe SF7-AM (5 μM) for 30 min, washed, and incubated with FBS-free DMEM only (left), with 100 μM γ-KetoCM-1 (middle), or with γ-KetoTCM-1 (right) for 2 h. Cells were then washed and imaged in PBS. Scale bar: 100 μM. Reproduced from ref. 202 with the permission of Wiley-VCH Verlag GmbH & Co. KGaA, Weinheim, copyright 2018.

The Pluth group have developed a series of γ-keto thiocarbamate compounds for GT (Fig. 68(A) and (B)).^202^ γ-KetoTCMs uses a cascade reaction to produce COS and subsequent release of H_2_S. When the donor is activated, the *p*-nitroaniline released provides an optical response which correlates with the release of H_2_S.

## 5. Disulfides

### 5.1. ROS triggered RSSR release

**Fig. 69:**
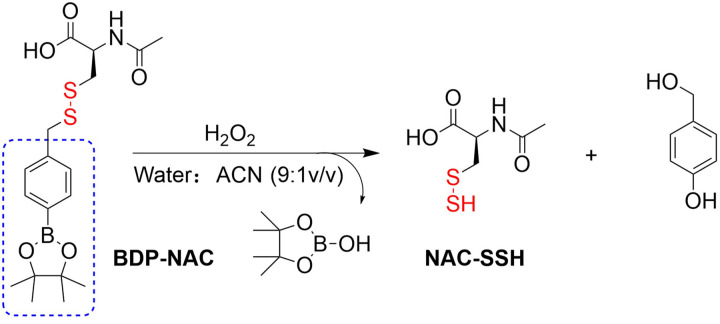
The structure of DOP-NAC, the mechanism of RSSH release.

Persulfides (RSSH) are considered to be an important signaling unit in sulfur atom-mediated oxidation reduction processes. However, the direct study of persulfides is difficult. Therefore, it is necessary to develop stable RSSH donors, which can help provide a deeper understanding of RSSR. The Matson group developed a ROS triggered RSSH donor (BOP-NCA) (Fig. 69).^203^*N*-Acetylcysteine was linked to a boronic acid ester *via* a disulfide bond, to obtain BOP-NAC. Subsequently, action of H_2_O_2_ on BOP-NAC triggers the releases of NAC-SSH and 4-hydroxybenzyl alcohol. In addition, the as-generated NAC-SSH moiety can protect cells against oxidative stress and can help maintain redox homeostasis better than the normal H_2_S donors Na_2_S and GYY4137.

**Fig. 70:**
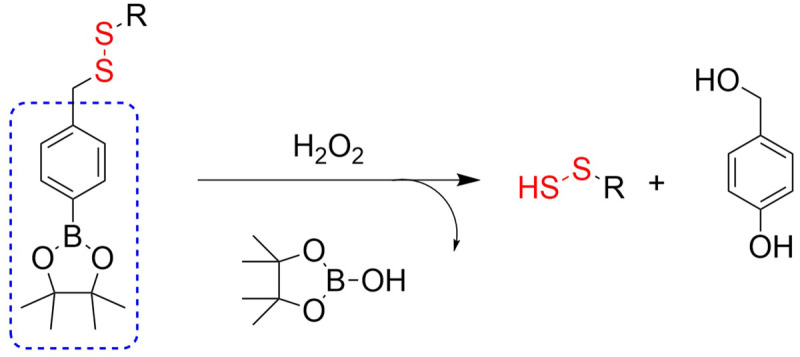
ROS triggered RSSR release.

**Fig. 71:**
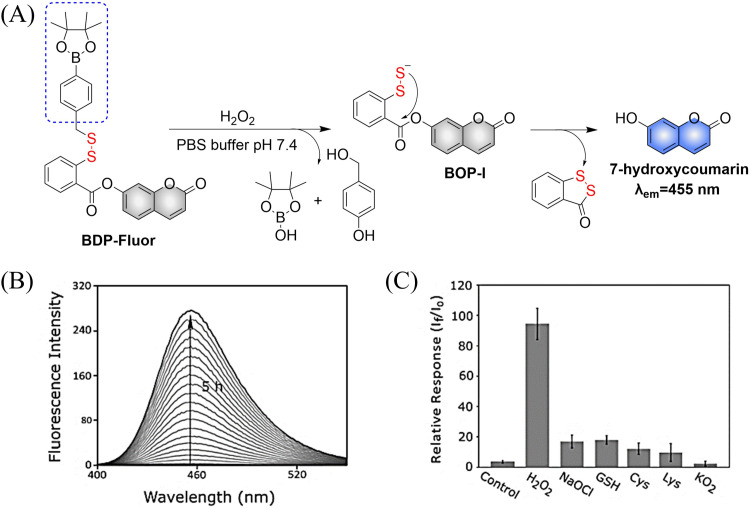
(A) The structure of BOP-fluor, the mechanism of RSSR release and its fluorescence changes. (B) Triggered by excessive H_2_O_2_, BOP fluor releases 7-hydroxycoumarin within 5 hours, resulting in a 100-fold fluorescence change. (C) The relative response of BDP fluor (3.3 μM) to each potential trigger (330 μM) or control (no trigger added). Reproduced from ref. 203 with the permission of John Wiley-VCH Verlag GmbH & Co. KGaA, Weinheim, copyright 2018.

Based on this design (Fig. 70) the disulfide terminal group was replaced with a fluorophore to generate the RSSR donor (BDP-fluor) (Fig. 71(A)–(C)). Intermediate BOP-1 generated under the action of H_2_O_2_ rapidly cyclizes to obtain the five membered benzodithiolone and releases 7-hydroxycoumarin enabling the visual monitoring of RSSR release.

**Fig. 72:**
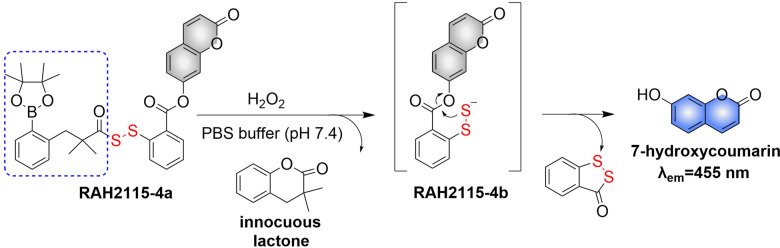
The structure of probe, the mechanism of RSSR release and its fluorescence changes.

Following on from the research of Xian^204^ and Matson,^203^ the Lukesh group developed a series of highly modular RSSR fluorescent donors (RAH2115-4a) (Fig. 72).^205^ Here we only focus on the RAH2115-4a donor that can produce RSSR under the trigger of hydrogen peroxide. Significantly the *gem*-dimethyl ensures the generation of acyl disulfide intermediates by RAH2115-4a donor, the exposed sulfur atom at the end of the disulfide bond can generate RSSR by nucleophilic attack on the ester group. Firstly, the donor is triggered by H_2_O_2_ to generate a non-toxic by-product; secondly, the acyl disulfide intermediate RAH2115-4b generates a five membered cyclic benzodithiolone by a rapid cyclization reaction. This reaction results in the release of 7-hydroxycoumarin which turns on the fluorescence output, the fluorescence intensity is proportional to the concentration of RSSR, providing a visual way to monitor the release of RSSR. This highly modular design has the potential to monitor any one of the numerous diseases associated with the uncontrolled production of ROS, such as cancer, inflammation, and cardiovascular disease.

## 6. Sulfur dioxide

### 6.1. Photo controlled SO_2_ release

#### 6.1.1. Diaryl ethene derivatives

**Fig. 73:**
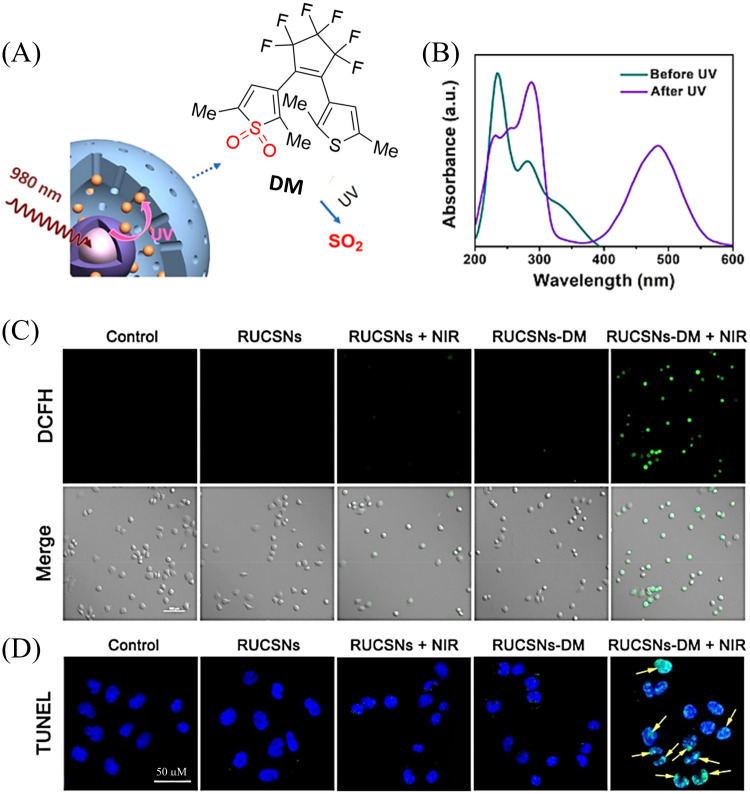
(A) Schematic illustration of NIR light-triggered SO_2_ generation from RUCSNs-DM. (B) UV-vis absorption spectra of DM before and after UV irradiation (365 nm). (C) Confocal imaging of intracellular ROS levels in HeLa cells after treatment with PBS (control), RUCSNs, RUCSNs + NIR (980 nm laser irradiation), RUCSNs-DM, RUCSNs-DM + NIR (980 nm laser irradiation) (D) Intracellular TUNEL staining in HeLa cells after treatment with different formulations (blue fluorescence: DAPI, green fluorescence: TUNEL). The yellow arrows indicated the overlap of blue fluorescence from DAPI and the green fluorescence from TUNEL, suggesting DNA fragmentation in the nucleus. Parts (A–D) are reproduced from ref. 208 with the permission of the American Chemical Society, copyright 2019.

Various SO_2_ donors have been engineered using molecules or nanoplatforms for therapeutic applications. With enhanced permeability and target specificity, they can effectively accumulate in pathological tissue (tumor acidic microenvironment) to enhance SO_2_ delivery.^206,207^ The Yang group developed NIR-light-triggered nanoparticles (RUCSNs) (Fig. 73(A)).^208^ The diaryl ethylene derivative DM is the source of SO_2_, since under UV irradiation, DM can efficiently release SO_2_ through C–S bond breaking or molecular dimerization (Fig. 73(B)).^209^ DM was encapsulated in the pores and internal cavities of the RUCSNs to form self-assembled nanoparticles RUCSNs-DM. RUCSNs-DM have high loading capacity and no obvious leakage, and can convert NIR into UV, thereby activating the donor to release SO_2_. Under the stimulation of a 980 nm laser, RUCSNs-DM can produce SO_2_, resulting in an increase of the intracellular ROS levels, causing DNA damage in the nucleus, and leading to cell apoptosis. In addition, the luminescence intensity of UCNPs with core–shell structure is about 20-fold that of nuclear UCNPs, which enables the visual-monitoring of intracellular SO_2_ release (Fig. 73(C) and (D)). This NIR-light-triggered SO_2_ therapy may provide an effective strategy for promoting the further development of synergistic cancer treatment platforms.

#### 6.1.2. 4,5-Dimethoxy-2-nitrobenzyl sulfonate

**Fig. 74:**
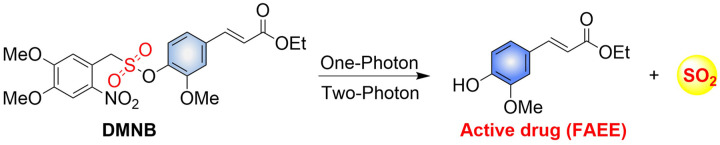
DMNB structure, mechanism of SO_2_ release, and fluorescence changes.

**Fig. 75:**
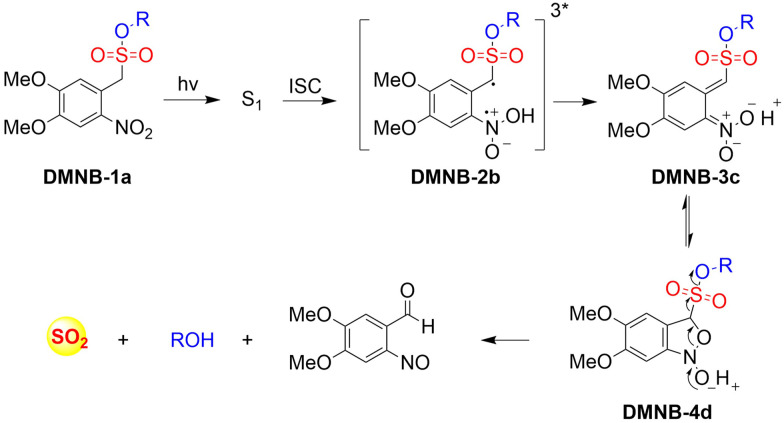
Proposed photodissociation mechanism of sulfonate DMNB-1a.

Molecules containing a sulfonyl functionality, such as sulfonyl chlorides or sulfonamides, have received extensive attention in the construction of SO_2_ donors (Fig. 70).^210^ The Singh group developed a single/two-photon activated SO_2_ donor (DMNB) (Fig. 74).^211^ The SO_2_ donor is directly connected to the photo trigger 4,5-dimethoxy-2-nitrobenzyl and the active drug *i.e.* ferulic acid ethyl ester (FAEE). Under light activation DMNB releases SO_2_ and a free hydroxyl (unmasked drug) (significant blue fluorescence is observed) through C–S bond breakage, realizing the dual release of gaseous transmitters and anticancer drugs, resulting in combination therapy and real-time monitoring of SO_2_ release in cells. According to previous reports, the authors proposed a photo triggering mechanism of sulfonate DMNB-1a (Fig. 75).^152^ First, under light activation, DMNB-1a is excited to a singlet state. Subsequently, it undergoes rapid ISC and transitions from singlet to triplet. In the triplet excited state, free radicals are generated at the benzyl and nitro positions of DMNB-2b to form *aci*-nitro DMNB-3c. Finally, a stable five-membered ring DMNB-4d was cleaved to produce 4,5-dimethoxy-2-nitrosobenzaldehyde, SO_2_ and hydroxyl containing therapeutic.

### 6.2. ROS triggered SO_2_ release

#### 6.2.1 Benzothiazole sulfinate

**Fig. 76:**
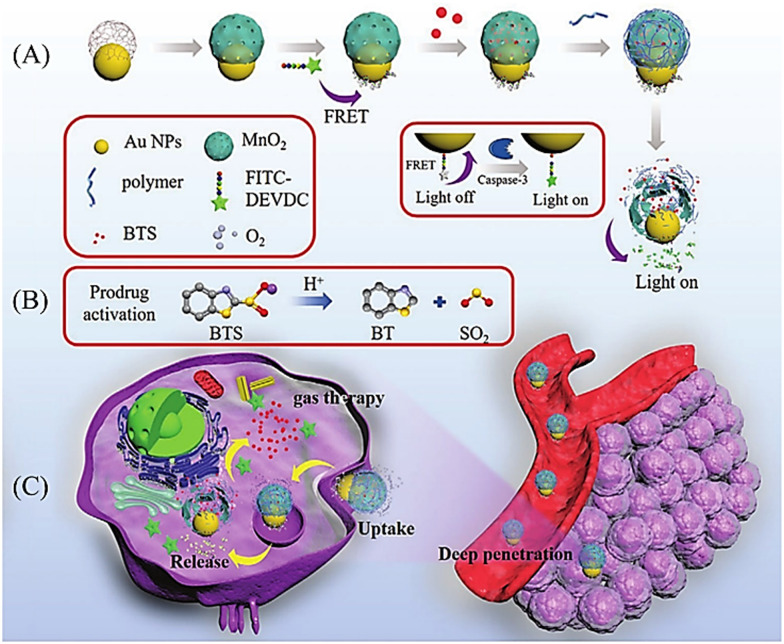
(A) Formation of Au@MnO_2_NPs nanoparticles. (B) and (C) Proposed decomposition mechanism of BTS releasing SO_2_ and self-reporting GT. Fluorescein isothiocyanate (FITC) is coupled to the surface of Au by caspase-3 responsive peptide (DEVEC). The fluorescence of FITC was quenched due to the FRET effect between Au NPs and FITC. However, caspase-3 is overexpressed in apoptotic tumor cells. As such DEVEC is cleaved and FITC fluorescence is restored to achieve “self-reporting” during the therapeutic process. Reproduced from ref. 212 with the permission of Wiley-VCH GmbH, copyright 2021.

GT nanoplatforms based on SO_2_ prodrug doping represent a significant development for effective cancer treatment. The Wang group developed nanoparticles (Au@MnO_2_NPs) loaded with SO_2_ prodrug (benzothiazole sulfinate, BTS) (Fig. 76(A)).^212^ The nanoplatform offers three unique advantages: (i) ultra-high drug loading due to its porous hollow structure; (ii) pH-dependent release and good water-solubility, and can be used for effective tumor accumulation and deep penetration; (iii) the combination of SO_2_ release and fluorescence response enables visualization and therapy of deep tumors. Under acidic pH conditions and in a high H_2_O_2_ environment, the MnO_2_ shell can catalyze the conversion of H_2_O_2_ to O_2_, and promotes the penetration of NPs into the tumor, improving the effect of GT. At the same time, MnO_2_ can be decomposed into Mn^2+^ in cells and release SO_2_ prodrug, benzothiazole sulfinate (BTS), for intracellular SO_2_ generation, resulting in oxidative stress damage and cell death. The overexpression of caspase-3 mediated by tumor cell apoptosis can lead to the release of free FITC due to the cleavage of the DEVEC peptide resulting in fluorescence enhancement, and self-reporting of the treatment process (Fig. 76(B) and (C)).

### 6.3. Thiol triggered SO_2_ release

#### 6.3.1. 2,4-Dinitrobenzenesulfonyl chloride

**Fig. 77:**
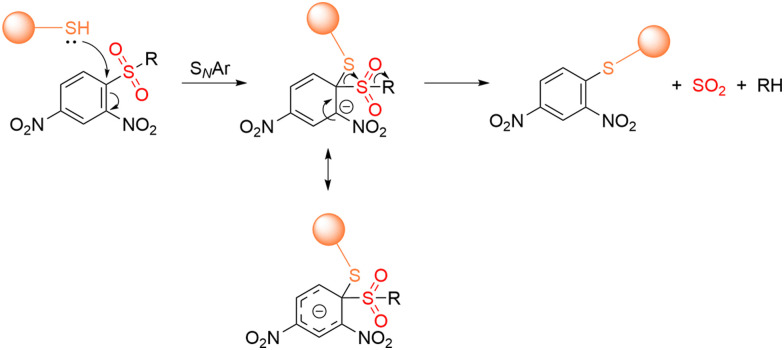
Under the trigger of GSH, 2,4-dinitrobenzenesulfonyl group releases SO_2_.

**Fig. 78:**
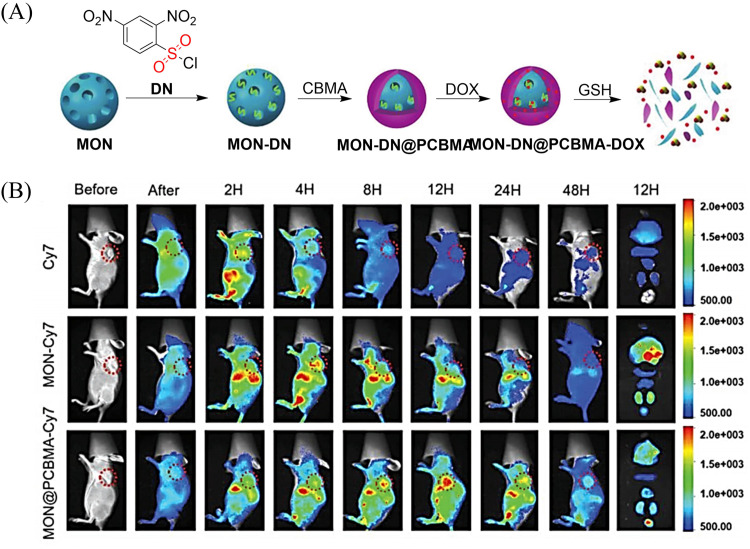
(A) Construction of a nano platform (MON-DN@PCBMA-DOX) and its SO_2_-release mechanism. (B) *In vivo* NIR-FI of tumor-bearing mice after injection of Cy7, MON-Cy7, and MON@PCBMA-Cy7 (1 mg mL^−1^, 100 μL). Reproduced from ref. 215 with the permission of Wiley-VCH Verlag GmbH & Co. KGaA, Weinheim, copyright 2020.

GSH is the most abundant intracellular reductive substance, and differences between normal and pathological levels provide an ideal cancer biomarker.^213^ In addition, GSH can specifically respond to 2,4-dinitrobenzenesulfonyl group, releases SO_2_ (Fig. 77).^214^ The Yang group developed a biodegradable material (MON-DN@PCBMA-DOX), both the MON core and PCBMA shell with disulfide bonds exhibit tumor microenvironment responsive biodegradation.^215^ MON-DN@PCBMA-DOX can be loaded with SO_2_ prodrug molecules (DN: 2,4-dinitrobenzenesulfonylchloride) and chemotherapeutics (DOX, doxorubicin) (Fig. 78(A)). In a GSH enriched tumor microenvironment, the S–S bond was broken, resulting in the release of SO_2_ and DOX, thereby realizing the synergistic treatment by GT and DOX. In addition, the generated SO_2_ molecules can sensitize cells to chemotherapy and overcome the multidrug resistance by downregulating the expression of *P*-glycoprotein (Fig. 78(B)).

#### 6.3.2. 2,4-Dinitrobenzenesulfonate

**Fig. 79:**
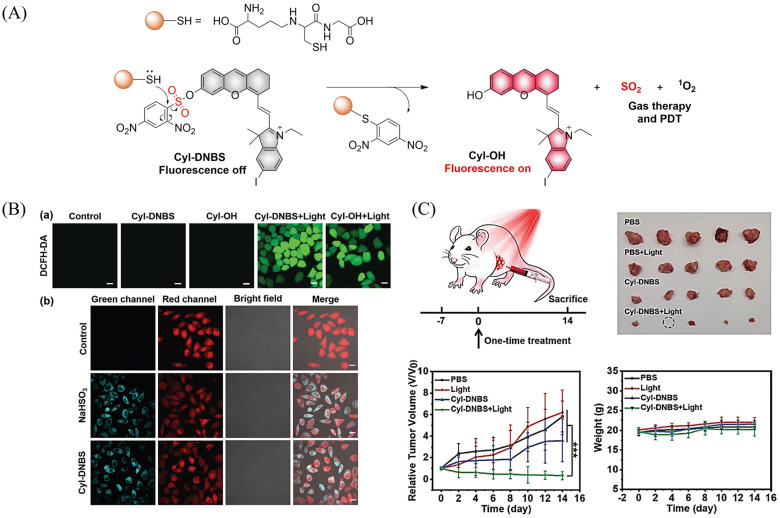
(A) The structure of Cyl-DNBS, the mechanism of SO_2_ release and its fluorescence change. (B) Cyl-DNBS generates ROS and SO_2_ fluorescence imaging in cells. (C) Effect of Cyl-DNBS on cancer mice. Parts (B) and (C) are reproduced from ref. 214 with the permission of Wiley-VCH GmbH, copyright 2021.

The Sun group linked 2,4-dinitrobenzenesulfonate (DNBS) with an iodine substituted hemicyanine scaffold (Cyl-OH) to prepare a GSH triggered SO_2_ PDT synergistic therapeutic donor (Cyl-DNBS) under red light irradiation (Fig. 79(A)).^214^ The donor exhibits good water solubility and positive charge which can be rapidly absorbed by cancer cells and localized at the mitochondria. In normal tissue, Cyl-DNBS is in the “off” state because the ICT process is locked, and the energy of the excited state is mostly released *via* non-radiative decay. However, in tumor tissue a high concentration of GSH triggers the generation of SO_2_ and simultaneous release of the active photosensitizer Cyl-OH. While, at the same time, the Cyl-DNBS was irradiated with 660 nm red light in the presence of O_2_. The photosensitizer (Cyl-OH) caused effective ISC and formation of a triplet state due to the “heavy atom effect” of iodine substitution, resulting in ^1^O_2_ which can be used for PDT treatment (Fig. 79(B) and (C)). This strategy realizes combined GT and PDT and improves the treatment efficiency toward cancer.

## 7. Reactive oxygen species

### 7.1. Singlet oxygen

**Fig. 80:**
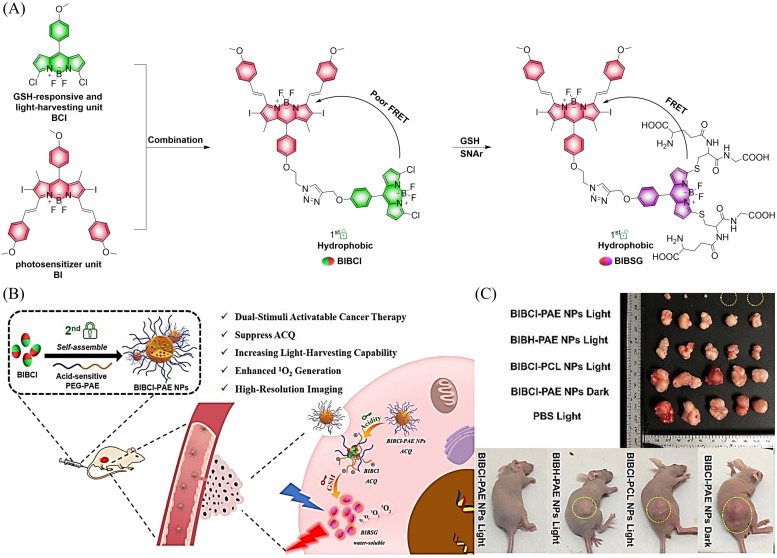
(A) The structure of BIBCl-PAE NPs, the mechanism of releasing ^1^O_2_ and its fluorescence changes. (B) and (C) Effect of PDT on tumors. Parts (B) and (C) are reproduced from ref. 219 with the permission of the Royal Society of Chemistry, copyright 2020.

PDT treatment mainly depends on the ^1^O_2_ concentration, which can lead to irreversible damage of cancer tissues.^216–218^ The Yang group reported a smart GSH/pH synergistically activated “dual lock-and-key” supramolecular photosensitizer BIBCl-PAE NPs (Fig. 80(A)).^219,220^ The authors encapsulate GSH-activated photosensitizer BIBCl (first lock-and-key) into a pH-responsive diblock copolymer (PEG-PAE) to construct BIBCl-PAE NPs (second lock-and-key) for enhanced PDT therapy. In normal tissues of neutral pH and low GSH, the hydrophobic nature of BIBCl and amphiphilic polymer PEG-PAE act as “double locks” to lock the photosensitizer in a tight aggregated state, which cannot sensitize oxygen to ^1^O_2_ due to aggregation-caused quenching. While in the tumor microenvironment, BIBCl is activated by a low pH environment and high GSH concentrations. The decomposition of micelles promotes the reaction between BIBCl and GSH, releases BIBSG and realizes efficient PDT (Fig. 80(B)). *In vitro* and *in vivo* experiments confirm that BIBCl-PAE NPs are effective in targeting and inhibiting carcinoma (Fig. 80(C)). In addition, using the FRET effect of BIBSG, enhanced ^1^O_2_ production and the visual-monitoring of PDT treatment were realized.

**Fig. 81:**
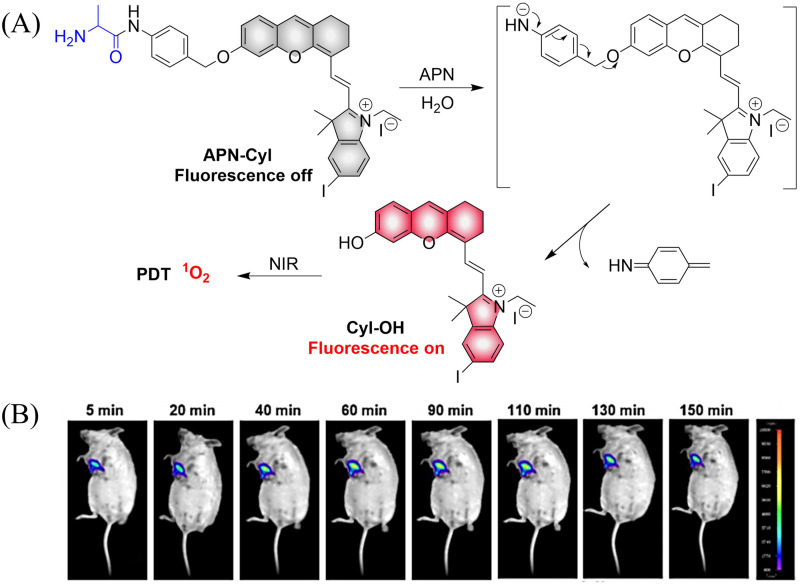
(A) Schematic illustration of APN-Cyl for APN imaging and cancer treatment. (B) Fluorescence imaging of endogenous APN in tumor Balb/c mice in 150 min. Reproduced from ref. 221 with the permission of Elsevier Ltd, copyright 2020.

Aminopeptidase N, APN/CD13 (APN) is overexpressed on the surface of cancer cells. The Peng group have developed a NIR photosensitizer (APN-Cyl) for tumor imaging and photodynamic therapy, which is specifically activated by APN (Fig. 81(A)).^221^ APN-Cyl could be activated by APN *via* hydrolysis of an alanine modified group to form Cyl-OH. The hydroxyl group in Cyl-OH has an ICT effect with the hemicyanine dye, which greatly improves the near-infrared fluorescence signals. In addition, Cyl-OH can specifically target mitochondria (positive charge in the structure) and generate a significant amount of ^1^O_2_ (due to the heavy iodine atoms) under NIR irradiation, thereby improving the efficacy of PDT and inducing apoptosis of cancer cells (Fig. 81(B)).

**Fig. 82:**
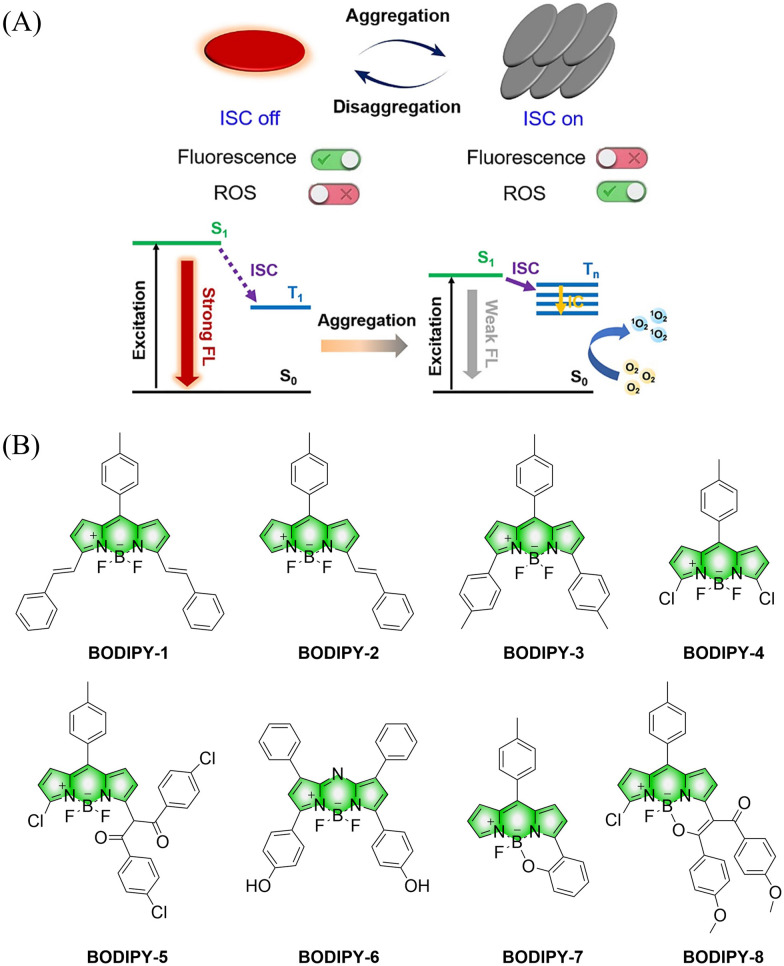
(A) The aggregated fluorophore is converted into photosensitizer (ISC on), and the fluorescence is quenched to produce ROS; the disaggregated photosensitizer is converted into a fluorophore (ISC off), and the fluorescence is restored without generating ROS. (B) BODIPY derivative structure. Reproduced from ref. 222 with the permission of the Chinese Chemical Society, copyright 2021.

Other donors with improved functions have also been reported. The Yang group have used the aggregation and disaggregation of fluorophores and photosensitizers (Fig. 82(A)).^222^ Heavy atoms are added to the fluorophore to enhance the spin orbit coupling (SOC) and to improve the ISC. This reversible switching is important for the development of a controllable PDT system. Compounds BODIPY1-8 exhibit fluorescence quenching in the aggregated state, leading to the generation of ROS. Interestingly, disaggregation can restore fluorescence and prevent ROS generation (Fig. 82(B)). If the ISC process (S_1_ → T_*n*_) is enhanced, the chromophore can act as a sensitizer to transfer the excitation energy of its triplet excited state to oxygen molecules to generate ^1^O_2_, thereby enhancing the PDT effect. This research provides a new strategy for designing heavy atom-free PSs and paves the way for the development of intelligent PDT systems.

### 7.2. Superoxides

**Fig. 83:**
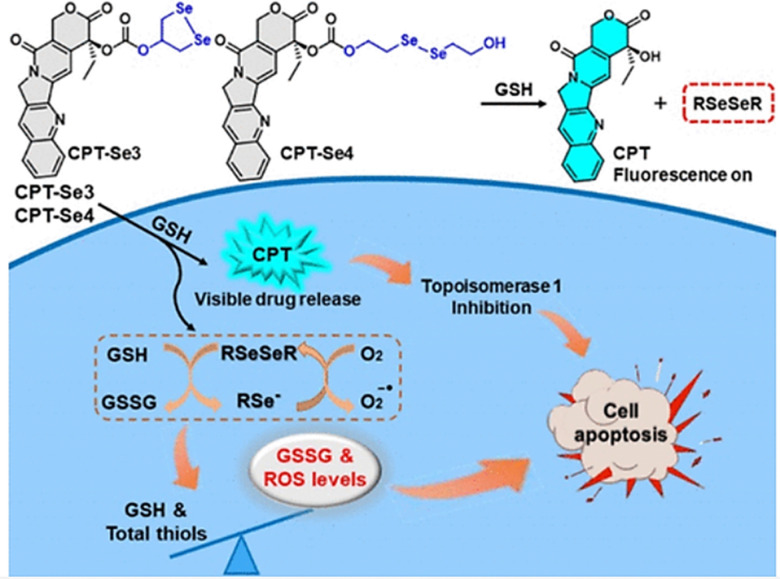
The structure of CPT-Se3 and CPT-Se4, the mechanism of CPT and O_2_˙^−^ release and the fluorescence changes. Reproduced from ref. 223 with the permission of the American Chemical Society, copyright 2021.

The Fang group introduced diselenated/disulfide units into camptothecin (CPT) and reported a series of seleno prodrugs CPT-Se3 and CPT-Se4 (the fluorescence of CPT was quenched by the diselenide/disulfide bond) (Fig. 83).^223^ Both selenium pro-drugs were activated by GSH, and the fluorescence of CPT was significantly enhanced (about 10-fold). The activation of the pro-drug is accompanied by the production of selenol intermediates, which catalyze the continuous conversion of GSH and O_2_ to oxidized glutathione and O_2_˙^−^. Thus, the level of ROS in cells is increased, and finally the apoptosis of cancer cells is induced. The authors confirmed that incorporating diselenide units into drugs may be a general strategy to improve drug efficacy. Furthermore, the quenching of CPT fluorescence by diselenide bonds suggests potential applications of diselenide bonds in the construction of sensors or therapeutic agents.

### 7.3. Hydroxyl radicals

**Fig. 84:**
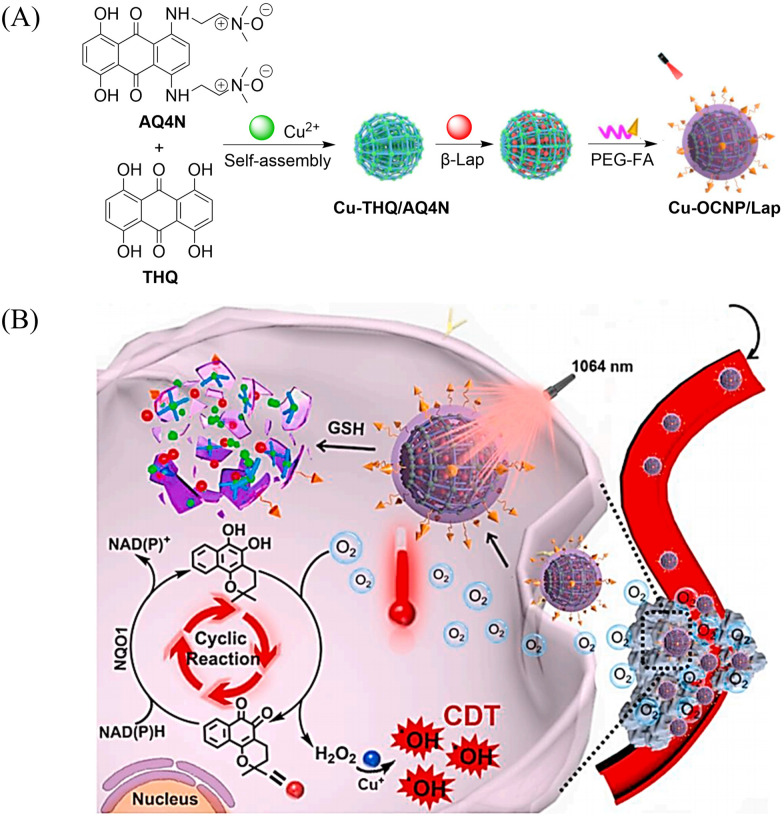
Schematic illustration of (A) synthesis of Cu–OCNP/Lap and (B) its intracellular delivery and NIR-II reinforced intracellular β-Lap cyclic reaction with abundant H_2_O_2_ supply to enhance CDT. Reproduced from ref. 226 with the permission of Elsevier Ltd, copyright 2021.

Chemodynamic therapy (CDT) can occur and induce apoptosis by converting H_2_O_2_ enriched in tumors into hydroxyl radicals (˙OH) with high cytotoxicity through the Fenton reaction.^224,225^ The Liu group have developed a self-assembled metal–organic coordination nanoparticle (Cu-OCNP/Lap) (Fig. 84(A)).^226^ Cu-OCNP/Lap exhibits good photothermal effect under 1064 nm light irradiation, the corresponding local temperature rise accelerates the blood flow and provides sufficient O_2_ at the hypoxic tumor site. The enhanced intracellular oxygen supply effectively reinforces the β-Lap redox cycling and results in abundant intracellular H_2_O_2_ accumulation, which facilitates the Cu^+^ Fenton-like reaction and effectively enhances CDT efficiency. In addition, significant amounts of GSH are consumed during the decomposition of the Cu-OCNP/Lap, which alleviates the antioxidant defense of the tumor microenvironment. Due to the PeT mechanism, Cu^2+^ in Cu-OCNP/Lap promotes the non-radiative transitions of AQ4N and THQ, thereby improving the photothermal effect. Initially, the fluorescence of Cu-OCNP/Lap is in a quenched state. Then when the GSH reduces the Cu^2+^ to Cu^+^, the fluorescence of AQ4N rapidly recovers enabling the visual monitoring of CDT treatment (Fig. 84(B)). The development of this donor provides a new concept for enhancing CDT treatment in a hypoxic environment.

## 8. Other gaseous transmitter donors

### 8.1. Photo controlled HCHO release

**Fig. 85:**
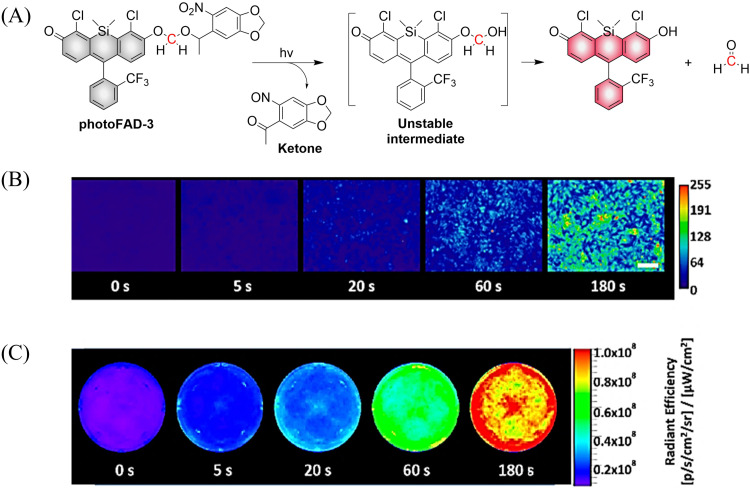
(A) Proposed decomposition mechanism of photoFAD-3 releasing FA. (B) Epifluorescence and (C) IVIS images of HEK293 cells stained with photoFAD-3 after 0, 5, 20, 60, and 180 s of photoactivation. Scale bar represents 100 μm. Parts (B) and (C) are reproduced from ref. 227 with the permission of the American Chemical Society, copyright 2020.

Despite numerous donors for the release of FA, the relationship between disease and formaldehyde concentration is still a major challenge owing to a lack of quantitative FA release strategies in biological systems. In order to understand the complex biological relationship of FA, the Chan group developed a photo-activated formaldehyde donor (photoFAD-3) (Fig. 85(A)).^227^ PhotoFAD-3 consists of the photon cleavable *o*-nitrobenzyl group and silicon–xanthene fluorophore linked by an acetal. Initially the fluorescence is quenched by PeT. However, under light irradiation, the *o*-nitrobenzyl group in photoFAD-3 is cleaved, and the fluorescence is enhanced about 139 times when FA is released, thus realizing the visual-monitoring of FA release. Moreover, a quantitative strategy to quantify the release of intracellular FA by cell lysate calibration was developed (Fig. 85(B) and (C)). Which marks the first example where the concentration of an intracellular analyte released from photoactivated donors could be quantified.

## 9. Conclusions and outlook

Exhibiting visible and controllable release of functional small molecules, FSMDs have enabled drug tracking, dose control, targeted delivery, spatiotemporal feedback, and precision medicine. This review focuses on the latest research strategies and applications of FSMDs: (i) the core structural design of FSMDs for the selective release of each type of signaling molecule (NO, CO, H_2_S, RSSH, SO_2_, ROS, H_2_Se, CO_2_ and FA, *etc.*) has been outlined, providing guidance in choosing appropriate chemical structures. (ii) The factors influencing the release of small molecules from FSMDs, including non-planar torsional conformation, the switch of electronic delocalization, molecule rearrangement, the effect of free radicals, molecular dynamic properties, *etc.* have been discussed, which highlights the underlying mechanisms of small molecule release coupled with fluorescence responses for FSMDs. (iii) FSMDs-based prodrugs, as well as their integration with gaseous therapy (GT), photodynamic therapy (PDT), photothermal therapy (PTT), or chemotherapy (CT), exhibit enhanced efficiency for drug screening and cancer treatment.

Although great progress in FSMDs research has been made, many challenges remain and opportunities for additional research remain.

(1) For *in vivo* applications. To date FSMDs have been mainly evaluated in cells, while *in vivo* applications remain mostly underexplored. Firstly, to address this challenge, FSMDs should be stable in a complex biological environment. We anticipate that developing suitable bioconjugates or integration with micelles should help alleviate these problems. Secondly, the effective depth of tissue penetration by the excitation and emission light are crucial for the effective use of FSMDs *in vivo*. Therefore, fluorophores with longer wavelengths or higher quantum yields are urgently required. Preferably, fluorophores in the NIR-II region should be used for the next generation of FSMDs.

(2) Practical efficiency. To achieve optimal properties for practical applications, the following aspects need be considered: firstly, targeted delivery and activation is essential for FSMDs. Though some classical recognition moieties have been used for the construction of FSMDs, the efficiency is dependent on the intracellular environment. Since the heterogeneous microenvironment of cells may enhance or hinder the performance of the FSMDs. Thus, introducing appropriate receptors to the molecular structure of FSMDs should be further explored. Secondly, the rapid triggering and release *in situ* requires an improved dynamic performance of FSMDs. So far, the dynamic evaluation of FSMDs especially in a biological environment remains limited. The rate of triggering of FSMDs, is essential since slow activation could result in inaccurate tracking. Thus, the rapid triggering of molecular rearrangement mechanisms is vital for practical applications in biological samples. Thirdly, the threshold to trigger FSMDs is essential to achieve the precise release of small molecules and real-time monitoring. Clearly, an appropriate threshold range that matches the specific biological environment should be optimized for triggering the FSMDs.

(3) Integration of multifunctionality. Cancer treatments can suffer drug resistance (chemotherapy), hypoxia (PDT), insufficient therapeutic effect (PTT), *etc.* Therefore, integration of FSMDs with these traditional techniques could provide a visible readout of the microenvironment, to overcome current drawbacks and improve efficiency. Small molecule intervention is of promise in the fight against cancer since it may help reverse the drug resistant microenvironment. For example, Near-infrared fluorescent probes for hydrogen sulfide: high-fidelity ferroptosis evaluation *in vivo* during stroke, and the synergistic effect of SO_2_ and DOX can effectively alleviate drug resistance in cancer chemotherapy. As such, multifunctional FSMDs could enable treatment coupled with visible evaluation, which may provide appropriate information to advance precision medicine.

## Abbreviations

AIE: Aggregation induced emission
APN: Aminopeptidase N, APN/CD13
aza-BODIPY: Aza-boron-dipyrromethene
BTS: Benzothiazole sulfinate
CA: Carbonic anhydrase
CBS: Cystathionine-β-synthase
CO: Carbon monoxide
CO_2_: Carbon dioxide
CORMs: Carbon monoxide-releasing molecules
COS: Carbonyl sulfide
CPT: Camptothecin
CSE: Cystathionine-γ-lyase
CT: Chemotherapy
DM: 1-(2,5-Dimethylthien-1,1-dioxide-3-yl)-2-(2,5-dimethylthien-3-yl)-hexafluorocyclopentene
DNBS: 2,4-Dinitrobenzenesulfonate
DOX: Doxorubicin
ESIPT: Excited-state intramolecular transfer
FA: Formaldehyde
FAEE: Ferulic acid ethyl ester
FHMA: 3-Formyl-4-hydroxybenzyl methacrylate
FITC: Fluorescein isothiocyanate
FRET: Fluorescence resonance energy transfer
FSMDs: Fluorescent small molecule donors
GSH: Glutathione
GT: Gaseous therapy
Hcy: Homocysteine
H_2_O_2_: Hydrogen peroxide
H_2_S: Hydrogen sulfide
H_2_Se: Hydrogen selenide
IC_50_: Half maximal inhibitory concentration
ICG: Indocyanine green
ICT: Intramolecular charge transfer
ISC: Intersystem crossing
MSCs: Mesenchymal stem cells
MRSA: Methicillin-resistant *S. aureus*
NCL: Native chemical ligation
NIR: Near infrared
NIR-II: Near-infrared-II
NO: Nitric oxide
NOPD: NO photo donors
˙OH: Hydroxyl radicals
OGD: Oxygen and glucose deprivation
OP: One-photon
PA: Photoacoustic tomography
PDT: Photodynamic therapy
PEG: Poly-ethylene glycol
PeT: Photoinduced electron transfer
pH: Scale used to specify the acidity or basicity of an aqueous solution
PTT: Photothermal therapy
ROS: Reactive oxygen species
SBTHA: *s*-Benzoyl thiohydroxylamine
SOC: Spin orbit coupling
SO_2_: Sulfur dioxide
TCO: *Trans*-cyclooctene
SMDs: Small molecule donors
TDLN: Tumor-draining lymph node
TICT: Twisted intramolecular charge transfer
TP: Two-photon
UV: Ultraviolet
2TC: Bithiophene

## Author contributions

Guang Chen, Jing Yu, Jie Xu, Chao Wang, Siyue Ma, Qing Miao, Linlin Wang, Chen Wang, and Zhe Sun wrote and edited the original proposal and draft. Luling Wu and Simon E. Lewis contributed to the scientific illustrations and editing of the manuscript. Xinrui Ji, Yanfeng Yue, Yuxia Liu created the outline and optimized the contents for the review paper. Bo Tang and Tony D. James conceived the topic and revised the manuscript. All authors contributed to the final checking of the manuscript.

## Conflicts of interest

There are no conflicts to declare.
